# Chaperone proteins protect against desmin fragment amyloid aggregation

**DOI:** 10.1016/j.bpj.2026.04.038

**Published:** 2026-05-05

**Authors:** Erin M. Mulhearn, Ariel M. Alperstein

## Main text

(Biophysical Journal *125*, 1996–2011; April 21, 2026)

In the originally published version of this article, Table 2 contained a formatting error that was introduced during typesetting. For the column “Concentration of fragment,” the value of 0.5 mg/mL (128 μM) should have applied to the first three rows of cells, not just the first row.

This error has been corrected in the article online, and Cell Press apologizes for any confusion this may have caused.

