# Supplementary material for: Chaperone proteins protect against desmin fragment amyloid aggregation
Source: Biophys J. 2026 Mar 23;125(8):1996–2011. doi: 10.1016/j.bpj.2026.03.038 (PMC13088209; doi:10.1016/j.bpj.2026.03.038)
Supplement: Supplementary file 1 [file mmc1.pdf]

Biophysical Journal, Volume *125*

Supplemental information

Chaperone proteins protect against desmin fragment amyloid aggregation

Erin M. Mulhearn and Ariel M. Alperstein

Supplemental Materials and Methods for

Chaperone proteins protect against desmin fragment amyloid aggregation

Erin M. Mulhearn[1] and Ariel M. Alperstein[1,*]

[1]Department of Chemistry and Biochemistry, University of Delaware, Newark Delaware
*Correspondence: amalpers@udel.edu

This Supplemental PDF file includes:

Figures

S1: Desmin fragment forms amyloid under acidic conditions
S2: SDS PAGE gel after crosslinking indicating purchased desmin fragment starts predominantly in monomeric form
S3: ThT fluorescence limit of detection for desmin fragment amyloid
S4: TEM data for all samples in main text Figure 1
S5: Additional TEM data supporting main text Figure 2E
S6: Additional TEM data supporting main text Figure 2F
S7: Additional TEM data supporting main text Figure 3B
S8: Additional TEM controls supporting main text Figure 3
S9: SDS PAGE gel after crosslinking potentially indicating active chaperones are in ≥12mer form
S10: Top scoring HDOCK models of desmin fragment + αB-crystallin with overlaid XL-MS observed cross-links
S11: Top scoring HDOCK models of desmin fragment + HSP27 with overlaid XL-MS observed cross-links
S12: XL-MS intra-links of chaperone proteins αB-crystallin and HSP27
S13: TEM data supporting main text Figure 5
S14: Bio-layer interferometry showing that chaperone αB-crystallin is capable of binding to both (A) desmin monomer and (B) desmin seed
S15: DLS data supporting main text Figure 5

Tables

S1: XL-MS observed cross-links with desmin fragment and αB-crystallin
S2: XL-MS observed cross-links with desmin fragment and HSP27

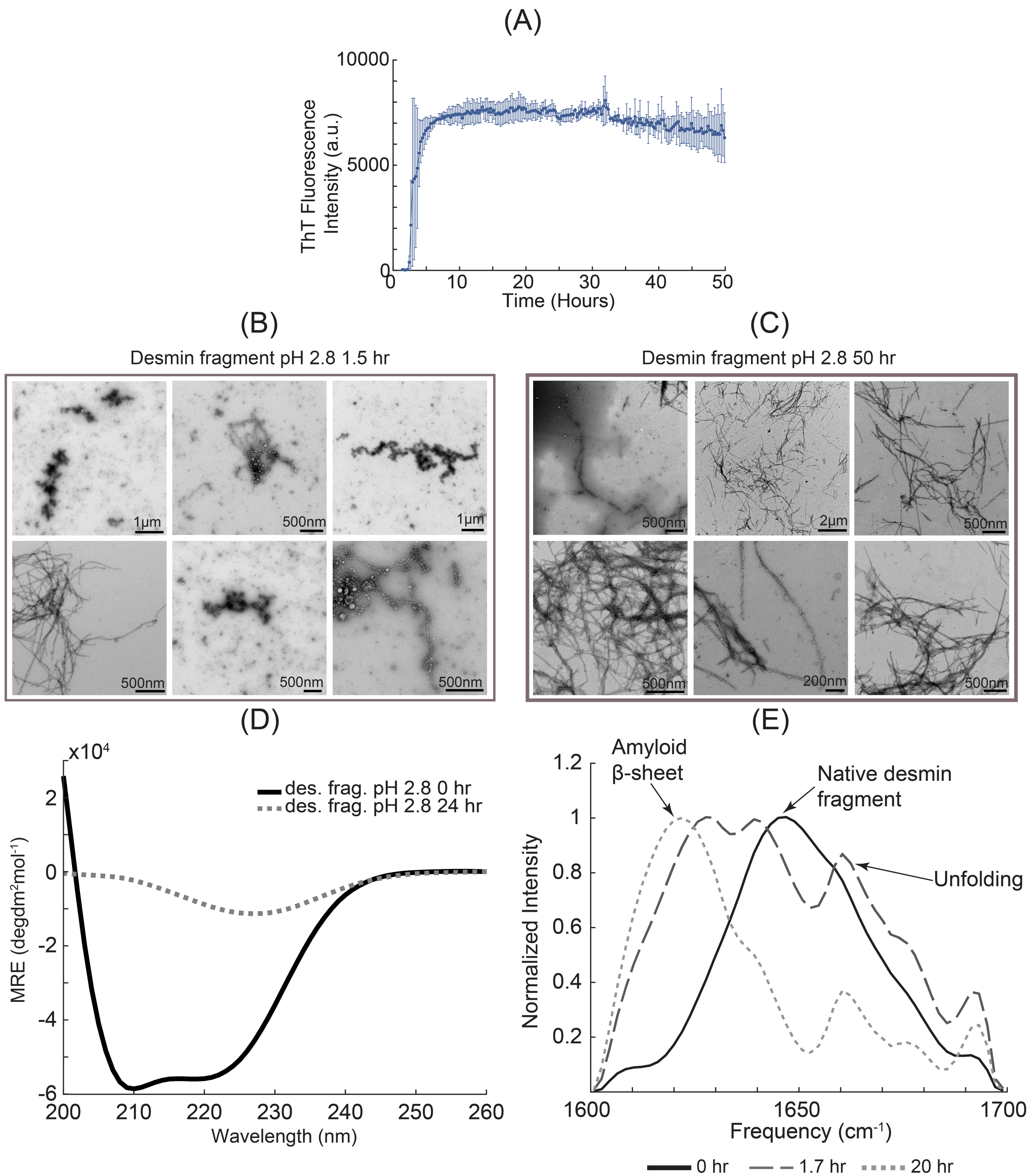

Figure S1: Desmin fragment forms amyloid under acidic conditions. (A) Desmin 256 μM at pH 2.8 amyloid kinetics as monitored by ThT fluorescence intensity. (B) TEM images were taken after 1.5 hour and (C) 50 hours into the aggregation process. (D) Circular dichroism spectra at time 0 (black) suggests desmin fragment after preparation has predominantly α-helical structure. After 24 hours into the aggregation process, the spectrum shows β-sheet structure, indicating aggregation. (E) FTIR spectra of desmin fragment pH 2.8. Color gradient (light to dark) shows amide I peak shift over time, forming amyloid.

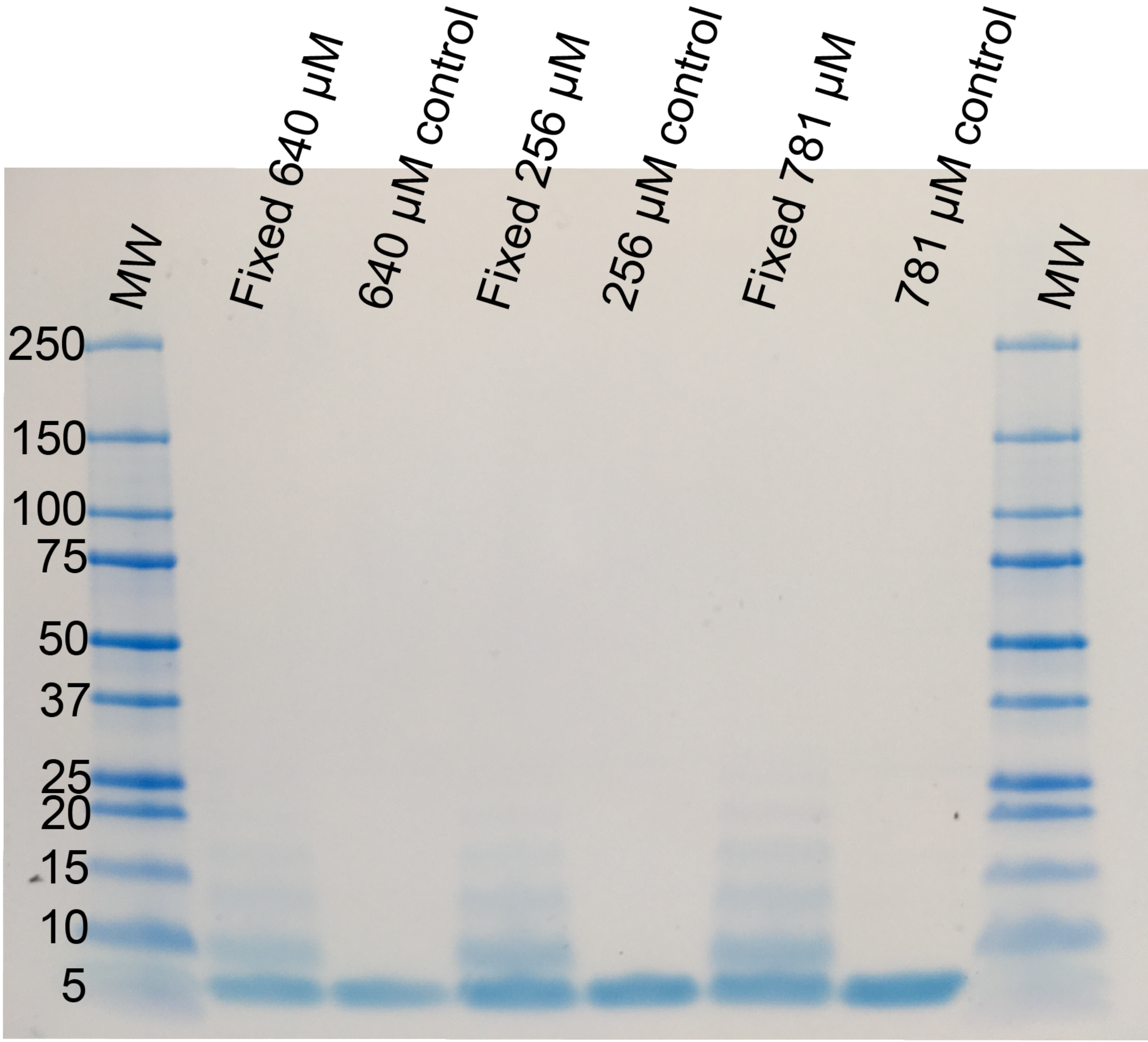

Figure S2: SDS PAGE gel after crosslinking indicating purchased desmin fragment starts predominantly in monomeric form. All fixed samples showed three faint higher molecular weight bands that represent transient oligomeric species but the majority of the samples exist as monomers. ImageJ calculations indicate at least 77% of desmin fragment is a monomer.

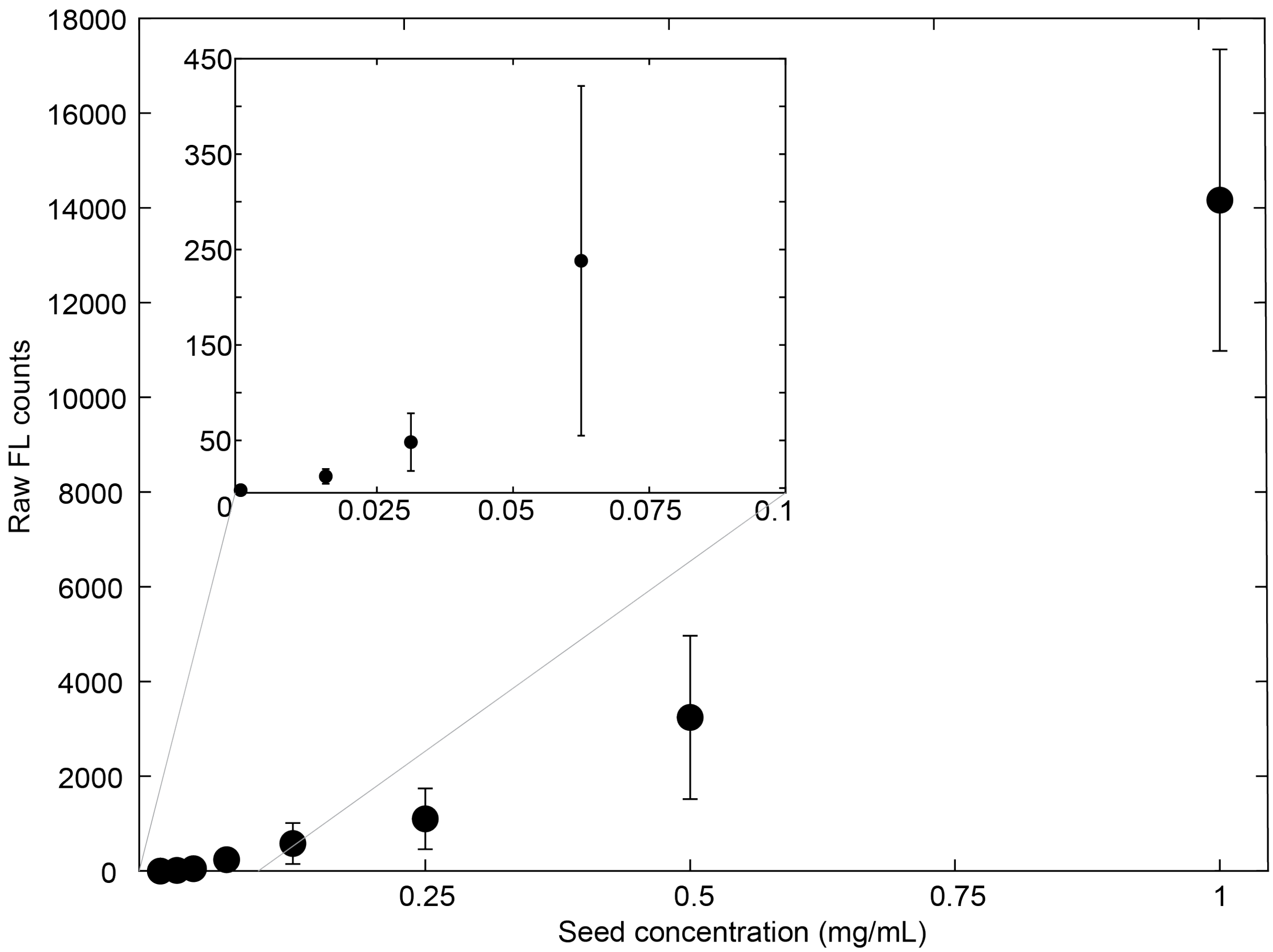

Figure S3: ThT fluorescence limit of detection for desmin fragment amyloid. ThT fluorescence intensity of desmin fragment seed at decreasing concentrations from 1 mg/mL (256 μM) to 0 mg/mL. The limit of detection for amyloid formation in our ThT assay is 0.06 mg/mL (15 μM). Triplicate raw data was averaged (N=3) and error bars are standard deviation.

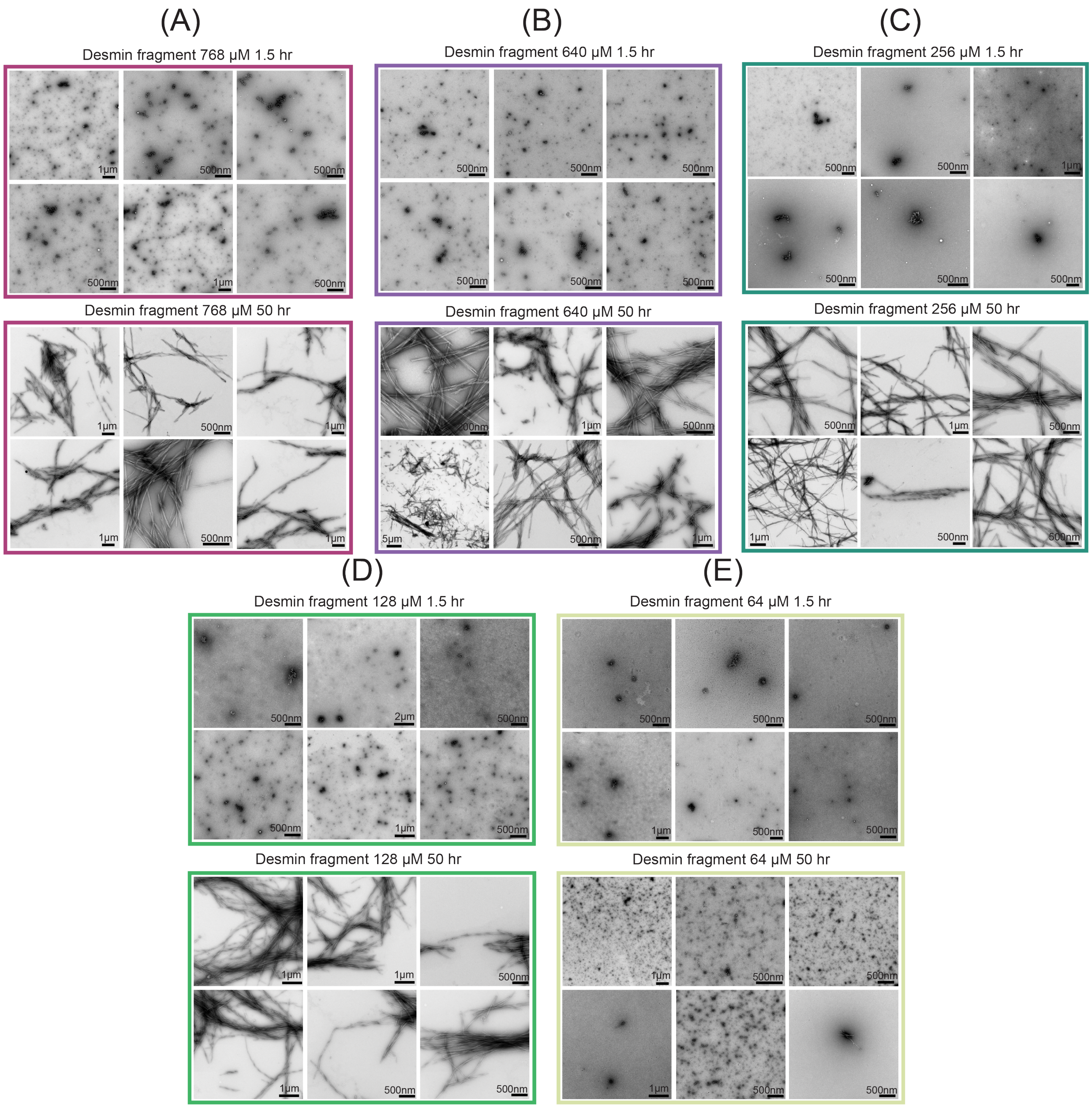

Figure S4: TEM data for all samples in main text Figure 1. (A-E) TEM images of desmin fragment at decreasing concentrations collected at time points 1.5 hours and 50 hours.

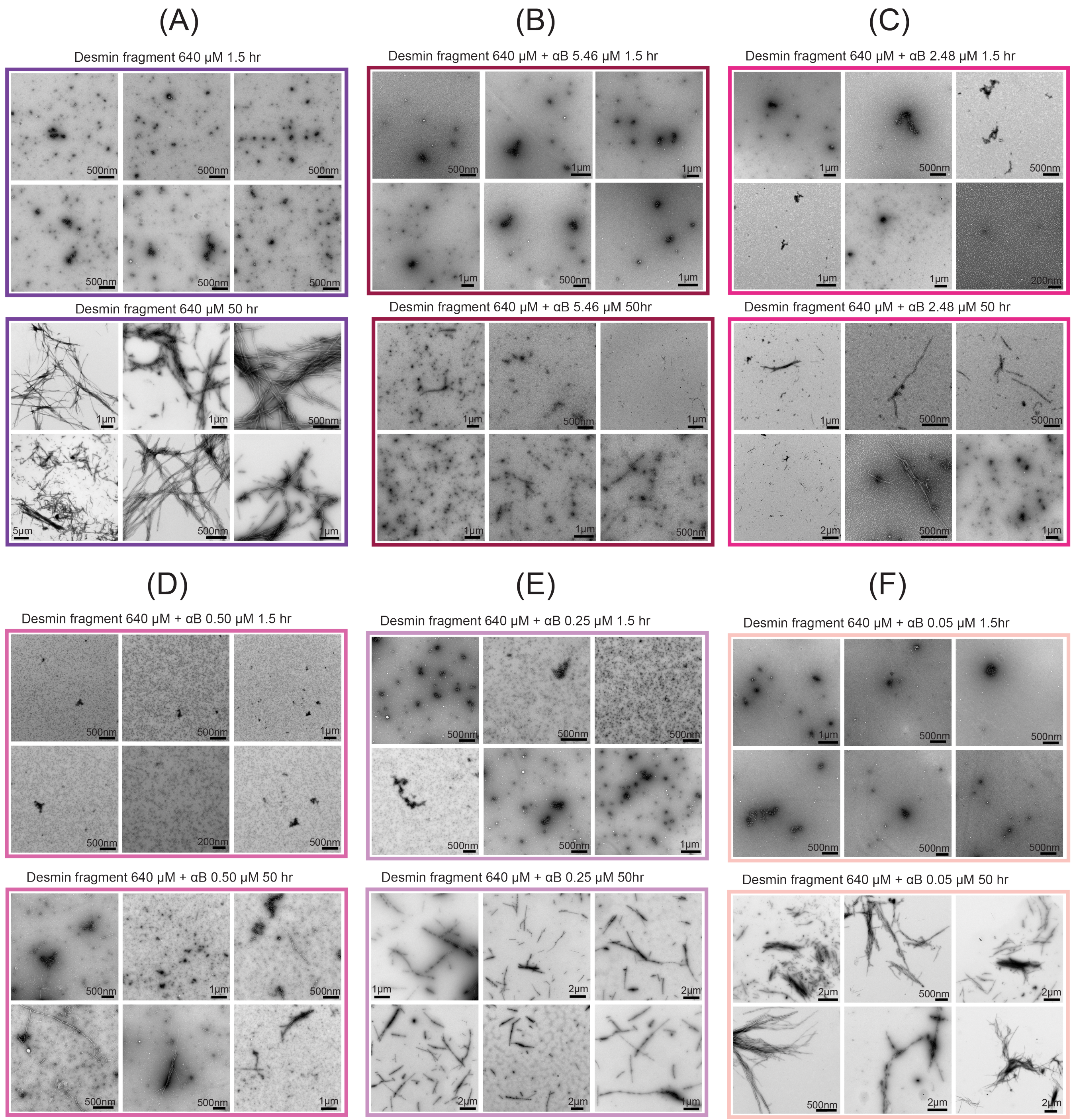

Figure S5: Additional TEM data supporting main text Figure 2E. (A-F) TEM images of desmin fragment at 640 μM and all concentrations of chaperone αB-crystallin (0.05 to 5.46 μM) at time points 1.5 hours and 50 hours.

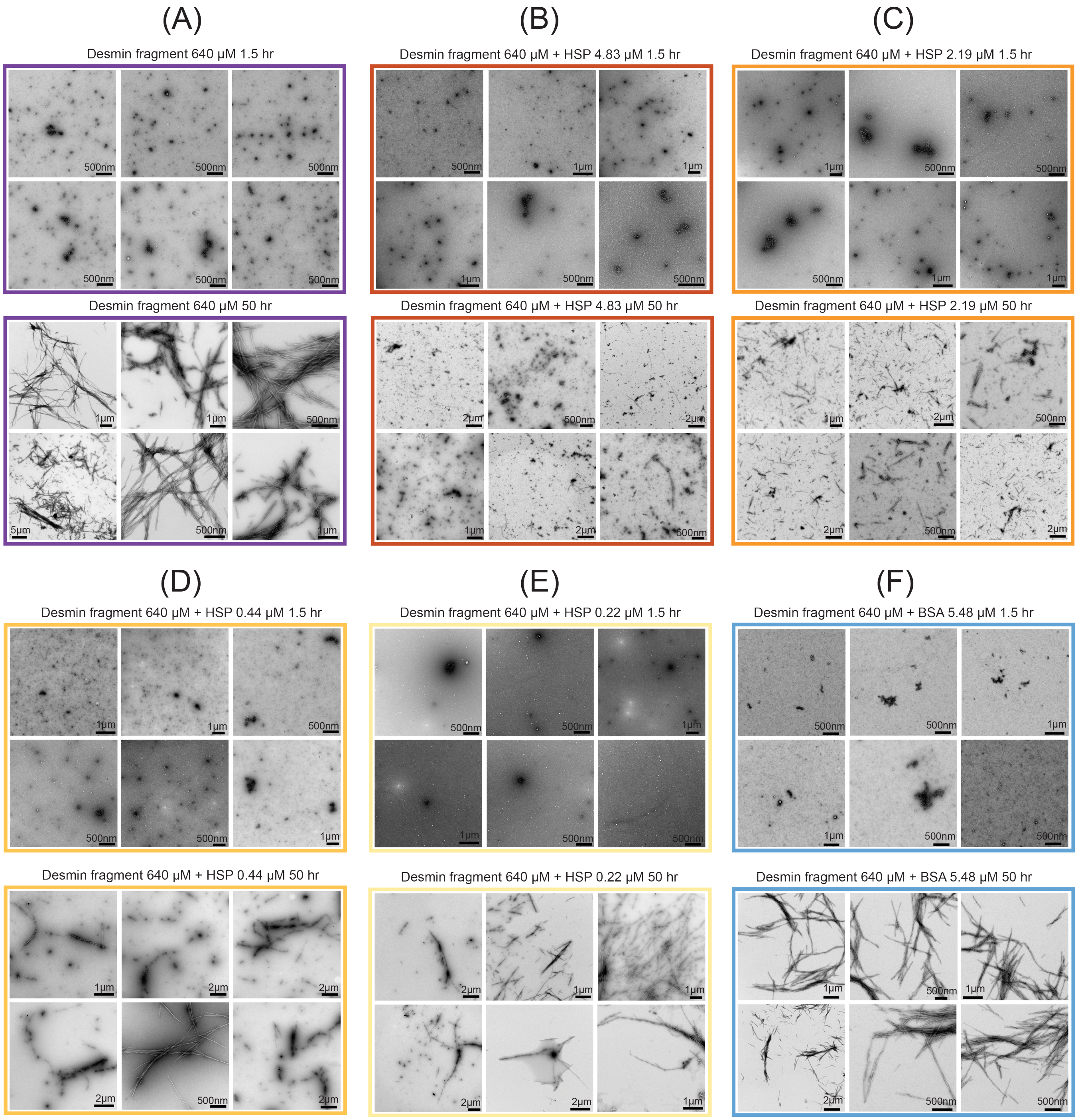

Figure S6: Additional TEM data supporting main text Figure 2F. (A-E) TEM images of desmin fragment at 640 μM and all concentrations of HSP27 (0.22 to 4.83 μM) at time points 1.5 hours and 50 hours. (F) Control sample of desmin with crowder BSA is also shown at time points 1.5 hours and 50 hours.

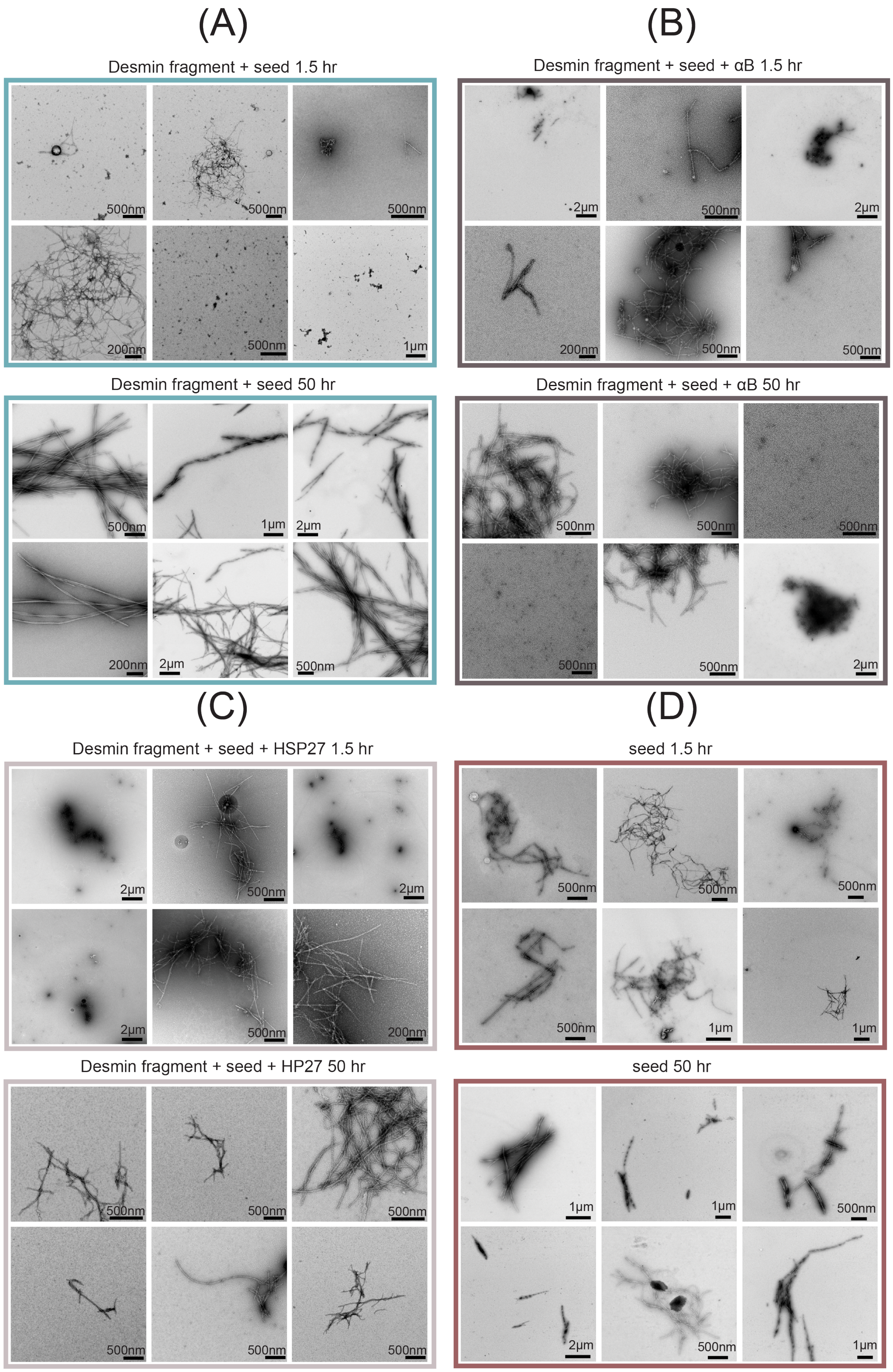

Figure S7: Additional TEM data supporting main text Figure 3B. (A-D) TEM images of desmin fragment + seed, desmin fragment + seed + αB, desmin fragment + seed + HSP27 and seed alone (control) at time points 1.5 hours and 50 hours. Concentrations were 128 μM desmin fragment monomer, 64 μM seed, 5.46 μM αB-crystallin, and 4.83 μM HSP27.

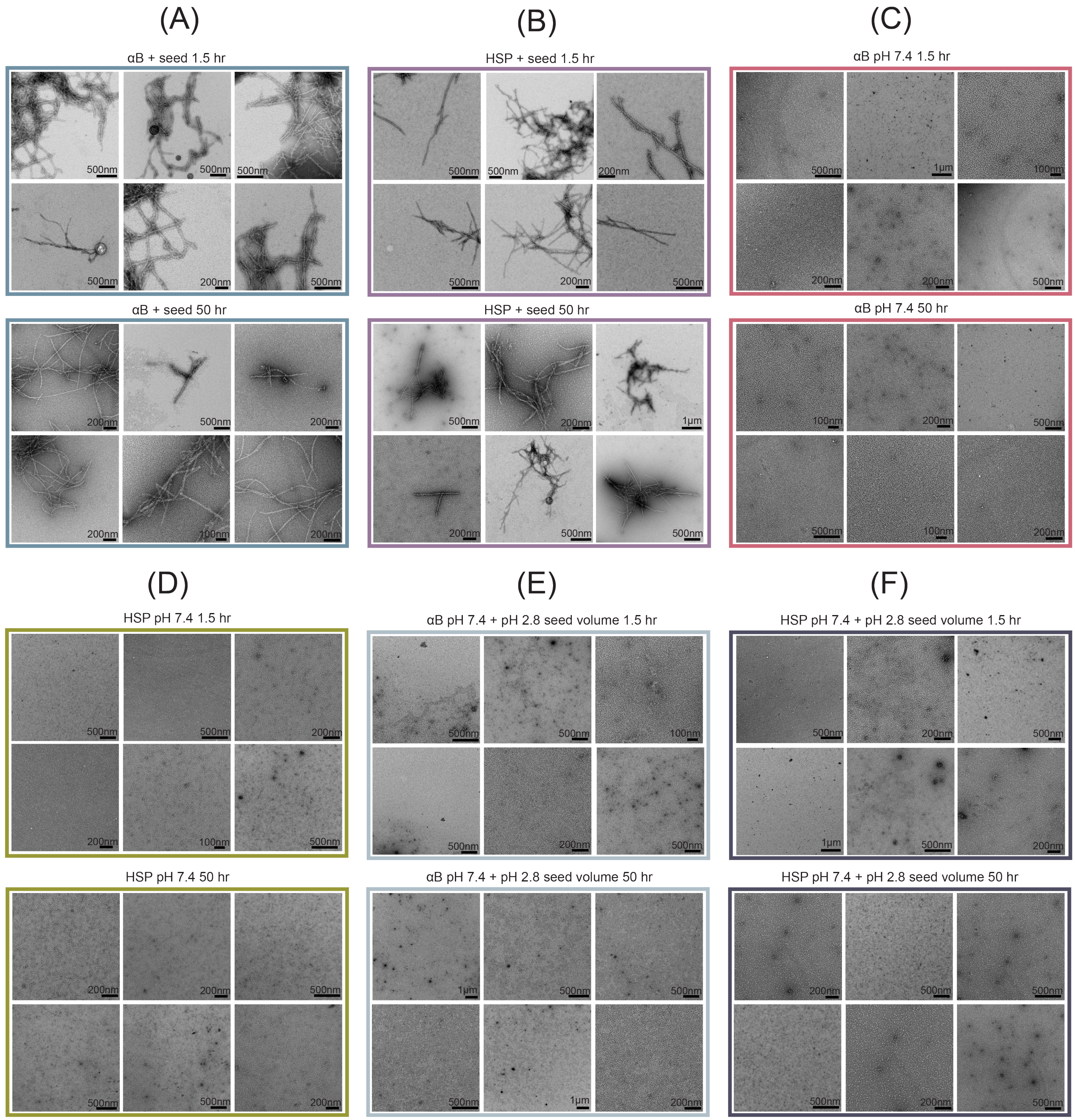

Figure S8: Additional TEM controls supporting main text Figure 3. (A-F) TEM images of chaperones + seed, chaperones at pH 7.4, and chaperones + pH 2.8 seed volume at time points 1.5 hours and 50 hours. Concentrations were 64 μM seed, 5.46 μM αB-crystallin, and 4.83 μM HSP27.

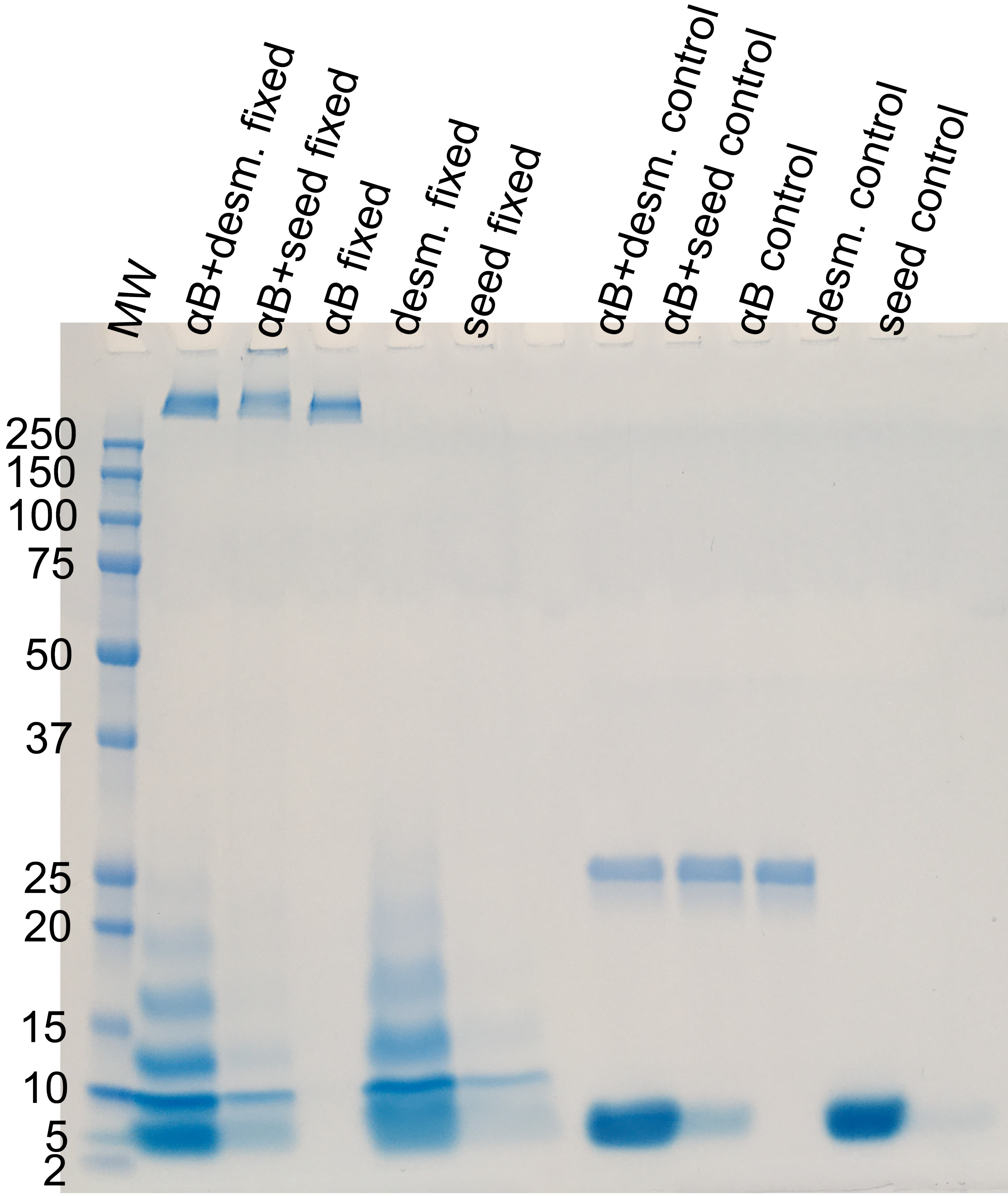

Figure S9: SDS PAGE gel after crosslinking potentially indicating active chaperones are in ≥12mer form. The fixed samples show that the chaperone does not show a band between 22 kDa and 250 kDa when combined with desmin fragment. If the chaperone was active as a dimer interacting with between 1 to 52 molecules of desmin fragment, or if active as a hexamer interacting with between 1 to 33 molecules of desmin fragment, or if active as a 12mer interacting with between 1 to 2 molecules of desmin, then we would see a band in this region.

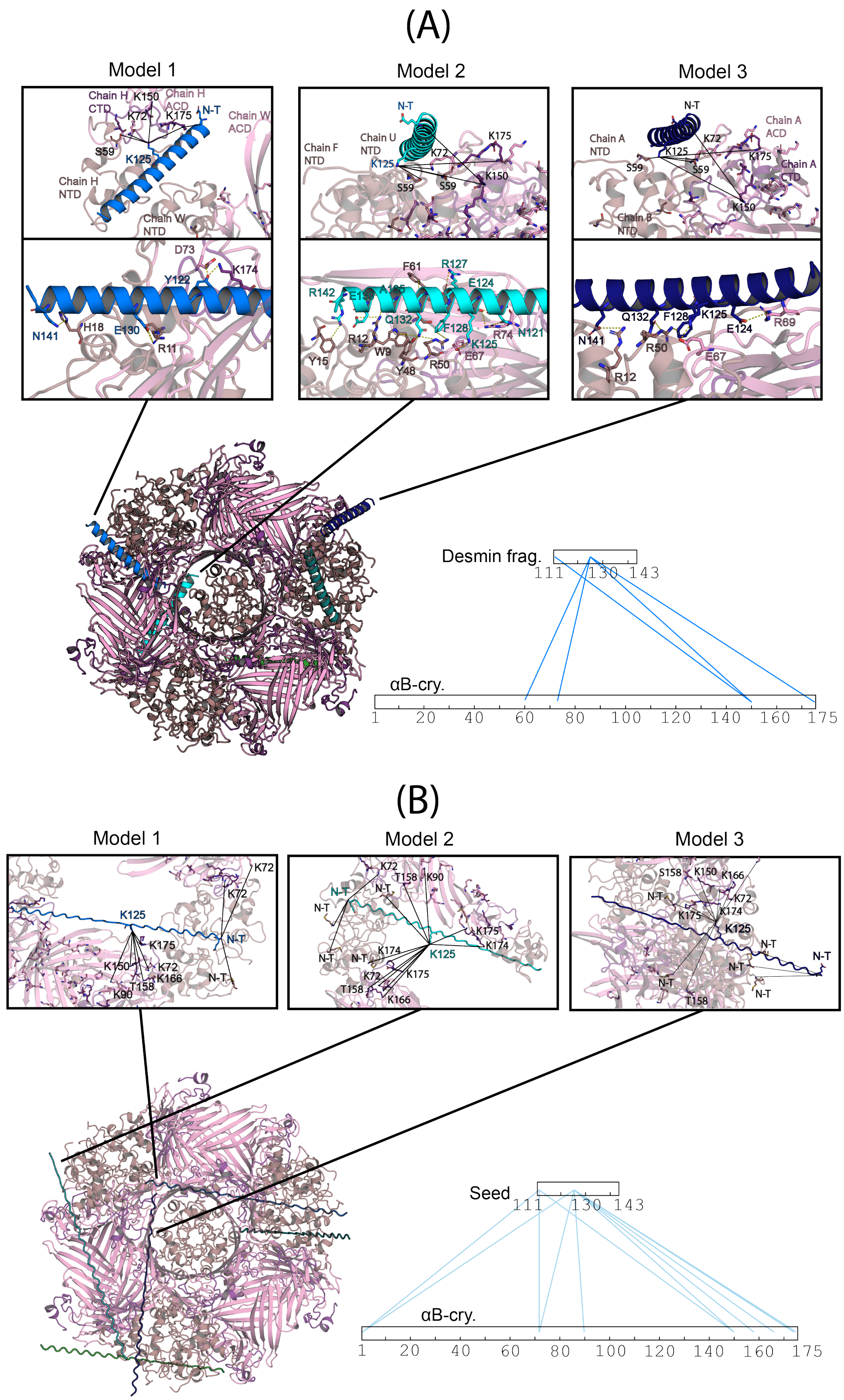

Figure S10: Top scoring Hdock models of desmin fragment + αB-crystallin with overlaid XL-MS observed cross-links. (A) Top 5 scoring Hdock models of desmin fragment monomer-αB-crystallin complex and (B) desmin fragment seed-αB-crystallin complex (model 1: blue, model 2: cyan, model 3: navy, model 4:dark teal, model 5: green). Top insets in A and B show the top three scoring HDOCK models with labels and lines indicating cross-linked residues from XL-MS. XL-MS cross-links shown between monomer and αB lines (dark blue, A bottom right) and seed and αB lines (light blue, B bottom right).

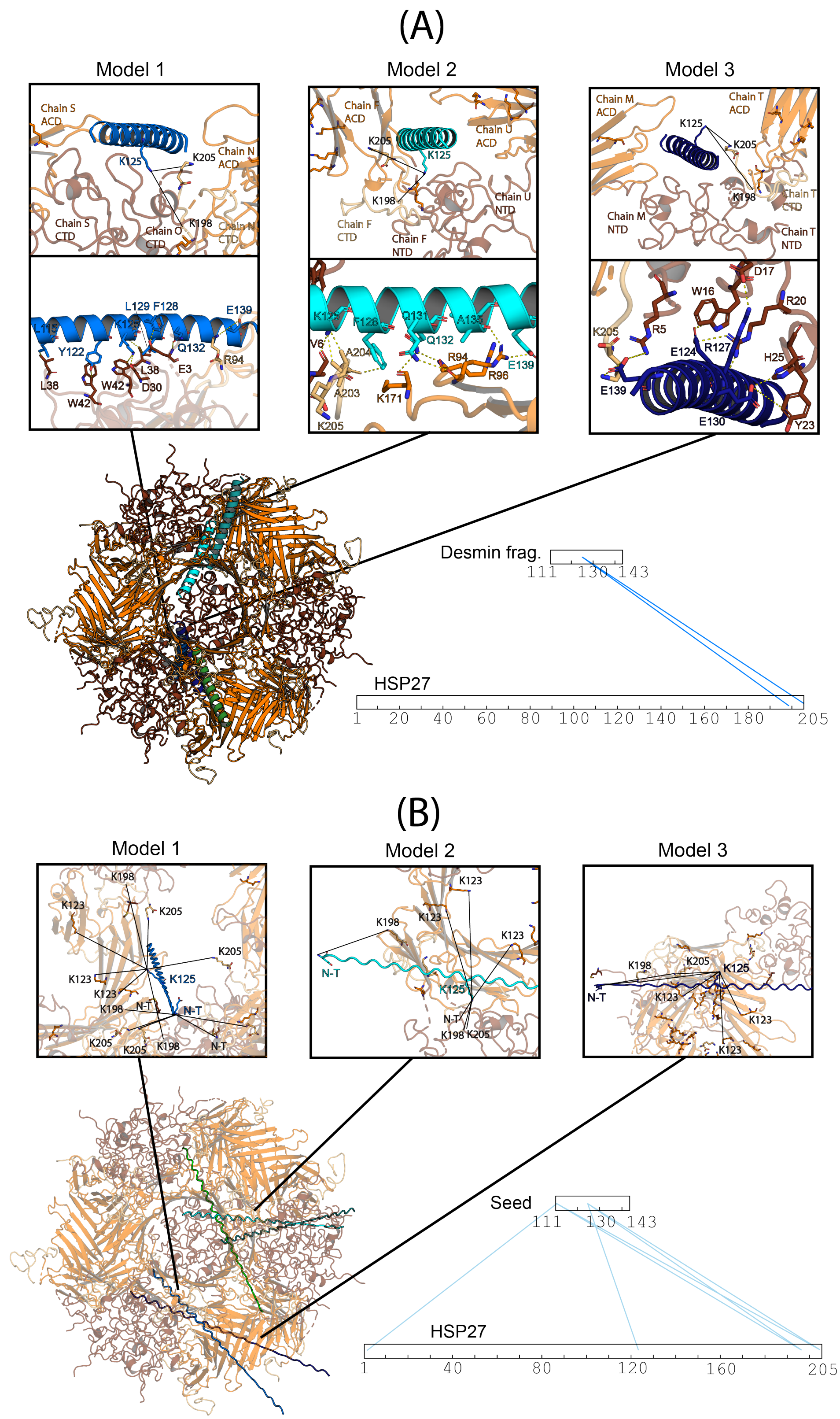

Figure S11: Top scoring Hdock models of desmin fragment monomer + HSP27 with overlaid XL-MS observed cross-links. (A) Top 5 scoring Hdock models of desmin fragment monomer-HSP27 complex and (B) desmin fragment seed-HSP27 complex (model 1: blue, model 2: cyan, model 3: navy, model 4:dark teal, model 5: green). Top insets in A and B show the top three scoring HDOCK models with labels and lines indicating cross-linked residues from XL-MS. XL-MS cross-links shown between monomer and HSP27 lines (dark blue, A bottom right) and seed and HSP27 lines (light blue, B bottom right).

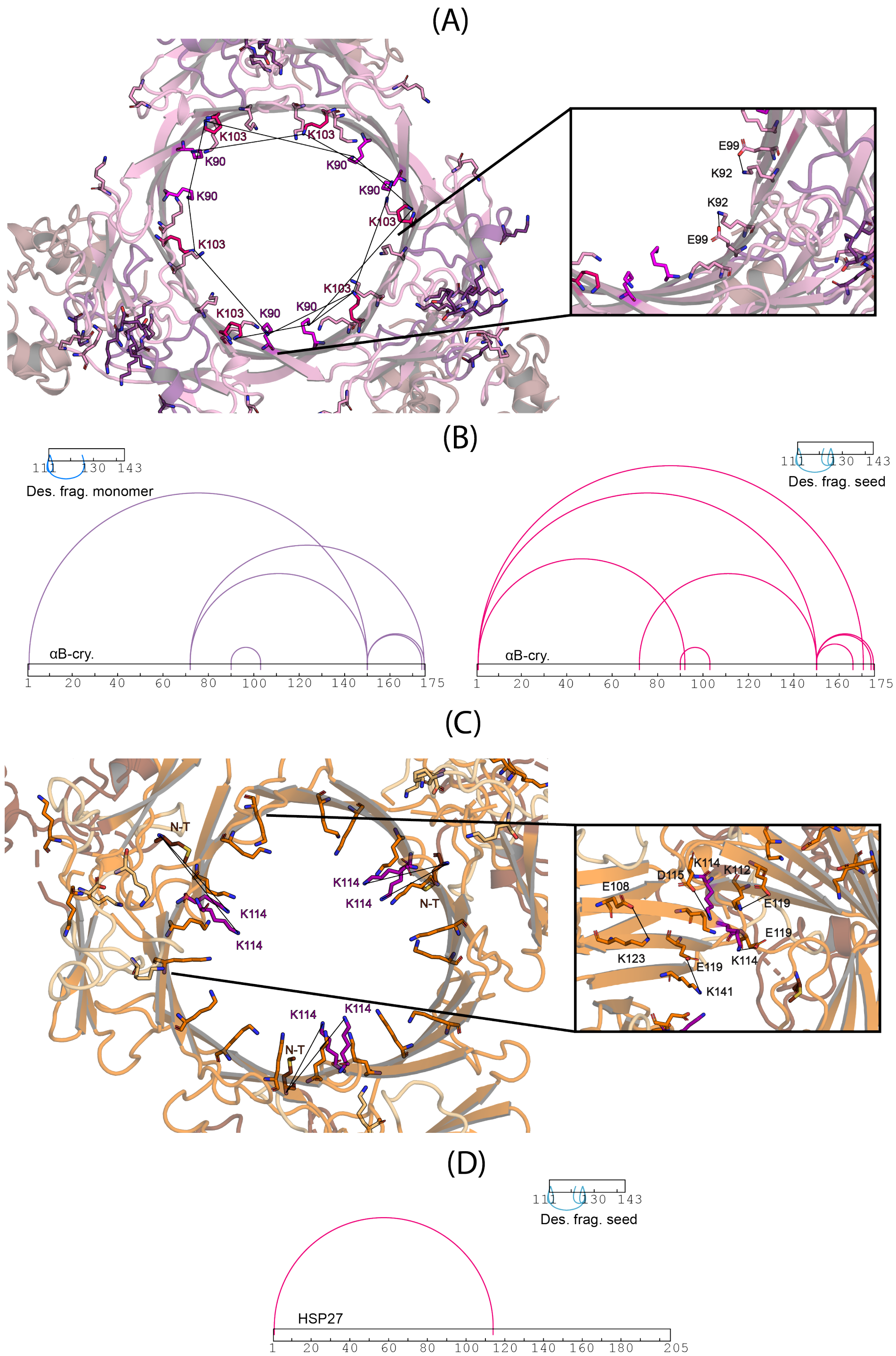

Figure S12: XL-MS intra-links of chaperone proteins αB-crystallin and HSP27. (A) αB-crystallin complex showing most of the lysines in the ACD point inward and are crosslinked to each other. Inset shows other lysines within the ACD form salt bridges to stabilize the dimer-dimer interface and are therefore inaccessible to interact with desmin fragment. (B) Intra-links (self-links) mapped onto αB-crystallin when incubated with desmin fragment monomer (left) or desmin fragment seed (right). Intra-links of desmin fragment monomer or seed are also shown. (C) HSP27 complex showing most of the lysines in the ACD point inward and are crosslinked to each other. Inset shows other lysines within the ACD form salt bridges to stabilize the dimer-dimer interface and are therefore inaccessible to interact with desmin fragment. (D) Intra-links (self-links) mapped onto HSP27 when incubated with desmin fragment seed, no intra-links were observed of HSP27 when incubated with desmin fragment monomer.

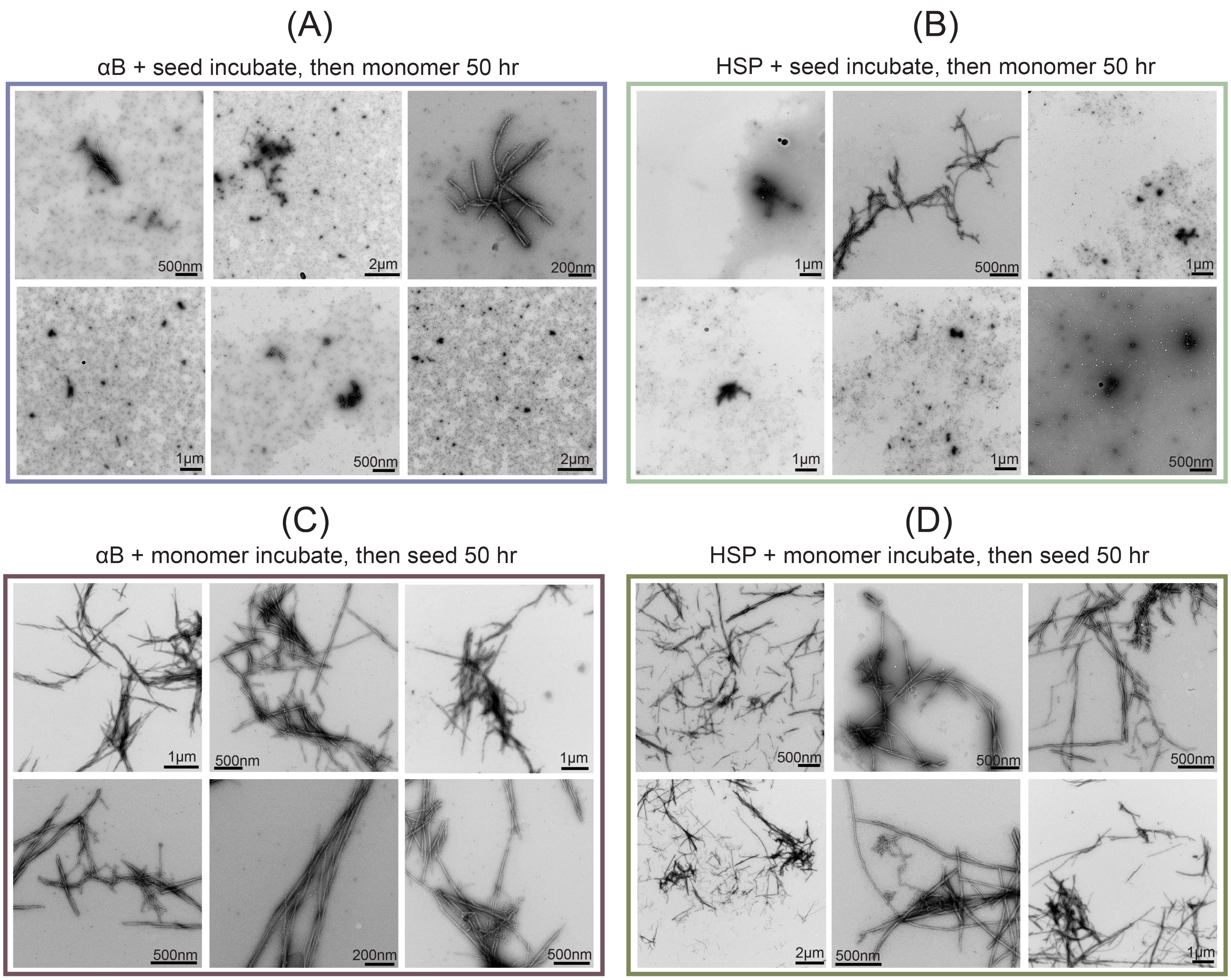

Figure S13: TEM data supporting main text Figure 5. (A-B) TEM images of chaperone incubated with seed first, and then monomeric desmin added. (C-D) TEM images of chaperone incubated with monomer first, then seed added. TEMs shown after 50 hours. Six representative images are shown for each condition.

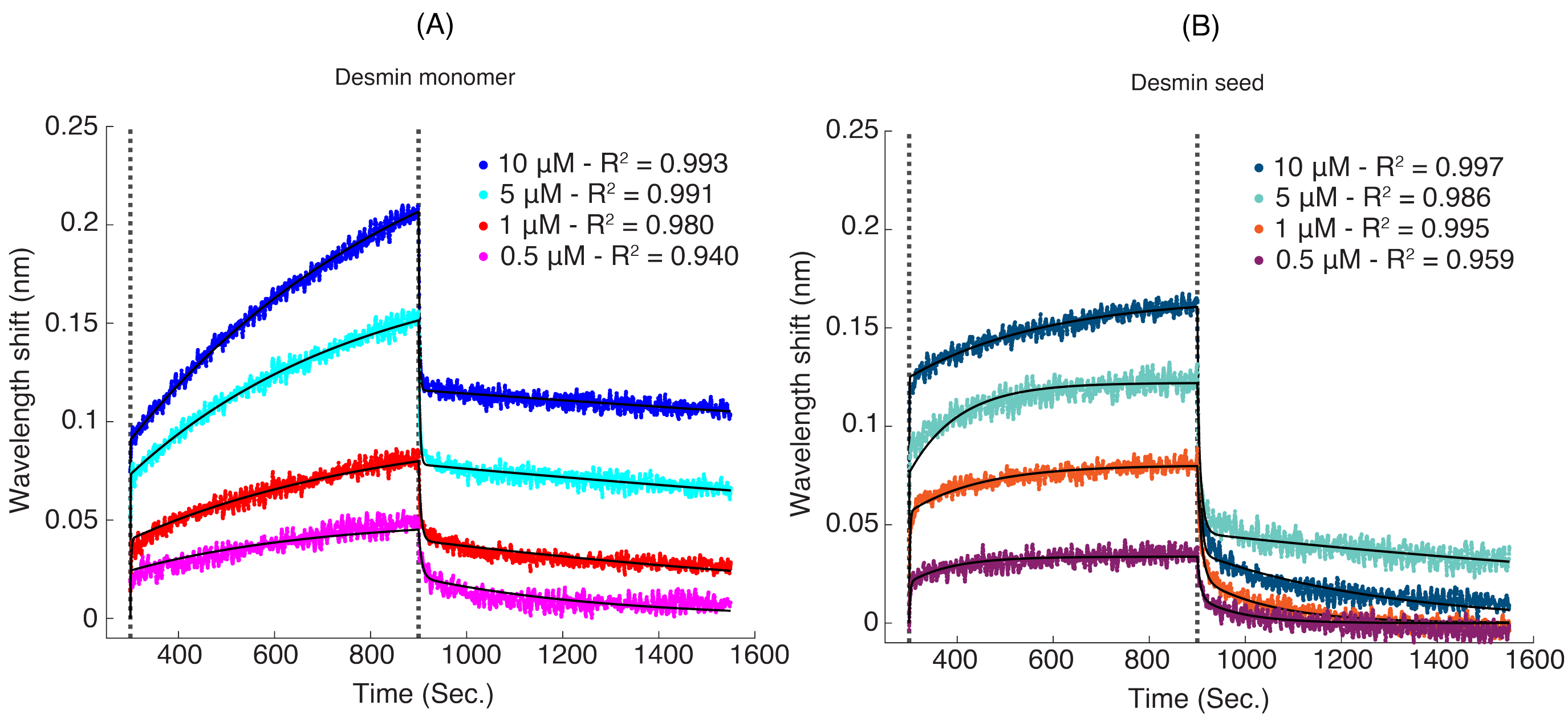

Figure S14: Bio-layer interferometry showing that chaperone αB-crystallin is capable of binding to both (A) desmin monomer and (B) desmin seed.

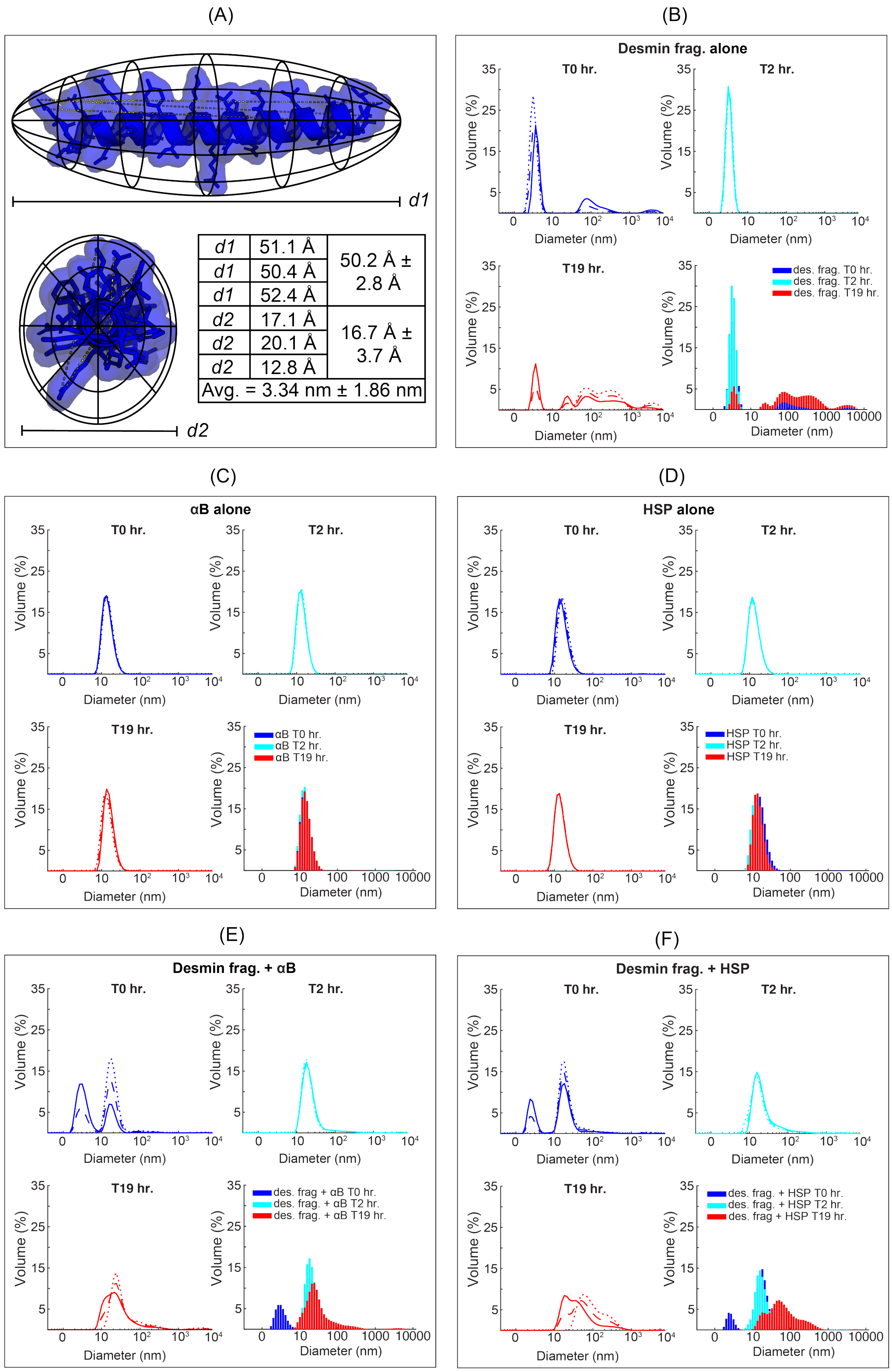

Figure S15: DLS data supporting main text Figure 5. (A) Pymol images of monomeric desmin fragment structure as predicted by AlphaFold showing the shape is best described by an ellipsoid with major (d1) and minor (d2) diameters. Averaged diameters are equal to a diameter of 3.34 nm ± 1.86 nm, similar to the observed hydrodynamic diameter of 3.4 nm. (B-F) Averaged intensity by volume plots of desmin fragment alone, αB-crystallin alone, HSP27 alone, αB-crystallin + desmin fragment, and HSP27 + desmin fragment at three different time points. Two independent preparations with triplicate dependent measurements were averaged. All measurements at each condition were then averaged (N=6 per condition).

Table S1: XL-MS observed cross-links with desmin fragment and αB-crystallin Observed cross-links between desmin fragment monomer or seed and αB-crystallin are shown. *Denotes where cross-links with a maximum XlinkX score below the cutoff threshold (>50) are included, since they were observed across other replicates.

Sample	Cross-linked fragments	Positions (Des. - Chap.)	Rep. number	Max XlinkX score
Des. frag. mono. + αB	FANYIE[K]VR-[K]QVSGPER	K125 - K150	1	88.68
			2	95.57
			3	70.98
			4	70.98
	FANYIE[K]VR-LE[K]DR	K125 - K72	1	83.00
			2	85.35
			3	N/A
			4	56.94
	FANYIE[K]VR-AP[S]WFDTGLSEMRLEK	K125 - S59	1	108.67
			2	N/A
			3	83.00
			4	N/A
	[+]ELQELNDR-[K]QVSGPER	N-term - K150	1	47.05*
			2	N/A
			3	72.62
			4	N/A
	FANYIE[K]VR-EEKPAVTAAPK[K]	K125 - K175	1	N/A
			2	N/A
			3	83.00
			4	68.55
Des. frag. seed + αB	[+]ELQELNDR-LE[K]DR	NTerm - K72	1	61.30
			2	N/A
			3	95.62
	FANYIE[K]VR-LE[K]DR	K125 - K72	1	87.66
			2	112.75
			3	107.79
	FANYIE[K]VR-[K]QVSGPER	K125 - K150	1	126.31
			2	125.48
			3	125.57
	[+]ELQELNDR-[K]QVSGPER	NTerm - K150	1	65.33
			2	79.03
			3	N/A
	FANYIE[K]VR-TIPITREEKPAVTAAPK[K]	K125 - K175	1	100.48
			2	74.21
			3	96.34
	[+]ELQELNDRFANYIEK-[+]MDIAIHHPWIR	NTerm - NTerm	1	65.80
			2	32.31*
			3	20.63*
	FANYIE[K]VR-HFSPEEL[K]VK	K125 - K90	1	142.43
			2	163.94
			3	105.98
	ELQELNDRFANYIE[K]VR-[+]MDIAIHHPWIR	K125 - NTerm	1	82.38
			2	71.74
			3	115.98
	FANYIE[K]VR-[T]IPITREEKPAVTAAPK	K125 - T158	1	N/A
			2	68.55
			3	82.38
	FANYIE[K]VR-TIPITREEKPAVTAAP[K]K	K125 - K174	1	96.46
			2	23.75*
			3	62.84

Table S2: XL-MS observed cross-links with desmin fragment and HSP27 Observed cross-links between desmin fragment monomer or seed and HSP27 are shown. *Denotes where cross-links with a maximum XlinkX score below the cutoff threshold (>50) are included, since they were observed across other replicates.

Sample	Cross-linked fragments	Positions (Des. - Chap.)	Rep. number	Max XlinkX score
Des. frag. mono. + HSP27	FANYIE[K]VR-AQLGGPEAA[K]SDETAAK	K125 - K198	1	55.43
			2	137.2
			3	N/A
	ELQELNDRFANYIE[K]VR-AQLGGPEAAKSDETAA[K]	K125 - K205	1	N/A
			2	124.43
			3	43.08*
Des. frag. seed + HSP27	FANYIE[K]VR-TKDGVVEITG[K]HEERQDEHGYISR	K125 - K123	1	136.48
			2	89.4
			3	118.78
	FANYIE[K]VR-AQLGGPEAA[K]SDETAAK	K125 - K198	1	127.22
			2	115.98
			3	147.92
	ELQELNDRFANYIE[K]VR-AQLGGPEAAKSDETAA[K]	K125 - K205	1	124.43
			2	108.67
			3	112.75
	[E]LQELNDRFANYIEK-[M]TER	NTerm - NTerm	1	176.33
			2	N/A
			3	191.86
	[+]ELQELNDR-AQLGGPEAAKSDETAA[K]	NTerm - K205	1	139.69
			2	N/A
			3	131.72
	[+]ELQELNDR-AQLGGPEAA[K]SDETAAK	NTerm - K198	1	107.73
			2	N/A
			3	35.11*
