# Supplementary material for: Chaperone proteins protect against desmin fragment amyloid aggregation
Source: Biophys J. 2026 Mar 23;125(8):1996–2011. doi: 10.1016/j.bpj.2026.03.038 (PMC13088209; doi:10.1016/j.bpj.2026.03.038)
Supplement: Supplementary file 2 [file mmc2.pdf]

Biophysical Journal
Article

Biophysical Society

# Chaperone proteins protect against desmin fragment amyloid aggregation

Erin M. Mulhearn[1] and Ariel M. Alperstein[1,*]
[1]Department of Chemistry and Biochemistry, University of Delaware, Newark, DE, USA

ABSTRACT In desmin-related cardiomyopathy, cellular stresses cause desmin to cleave and aggregate *in vivo*. Cleaved desmin fragments are amyloidogenic and induce misfolding, aggregation, and amyloid fibril formation of full-length wild-type desmin. Alongside this disease condition, cells overexpress $\alpha$B-crystallin and heat shock protein (HSP) 27 as cardioprotective chaperones. Chaperone proteins may refold, sequester, or disaggregate misfolded or aggregating client proteins. Previously, little was known about the protective influence of chaperone proteins on desmin fragment amyloid formation. Here, we studied the secondary structure changes, kinetics, and morphology of desmin fragment amyloid formation with and without chaperone proteins $\alpha$B-crystallin and HSP27. We found that both of these proteins prevent or delay amyloid formation in a concentration-dependent manner, but we did not observe amyloid disaggregation. We discovered better chaperone activity by $\alpha$B-crystallin than HSP27 toward desmin fragment, correlated to increased binding sites between $\alpha$B-crystallin and desmin fragment. Our interaction studies demonstrated that monomers or amorphous oligomers were likely reversibly captured by these chaperone proteins, whereas desmin fragment amyloid fibrils were likely irreversibly capped by chaperone proteins. Overall, $\alpha$B-crystallin and HSP27 recognized and interacted with both desmin fragment monomer and desmin fragment amyloid at distinct sites to prevent further amyloid formation.

SIGNIFICANCE This work represented a different approach to understanding the role of chaperones in modulating amyloid aggregation, offering potential insights into future strategies for understanding or treating desmin-related cardiomyopathies and other diseases linked to chaperone protein dysfunction. We studied chaperone inhibition of amyloid formation in the context of desmin fragment aggregation. We found that monomer+chaperone interactions appear to be reversible, and amyloid+chaperone interactions appear to be irreversible. While neither chaperone studied could disaggregate amyloids, we learned $\alpha$B-crystallin had better chaperone activity toward desmin fragment than HSP27. The knowledge we gained here about chaperones could influence future pharmacological chaperone development.

## INTRODUCTION

Heart disease has been the leading cause of death in the United States since 1921, accompanied by increasing rates of morbidity and mortality (1). Desmin, an intermediate filament protein integral to both cardiac and skeletal muscle, plays a critical role in cytoskeleton organization and maintenance of cardiomyocyte structure. Desmin impacts heart disease through cytotoxic aggregation and disruption of cellular organization (2).

*De novo* or inherited mutations of desmin cause cardiomyopathies by disrupting the desmin filament network and the accumulation of abnormal desmin in cardiomyocytes (3–8). Additionally, posttranslational modifications (9), proteolytic processes (10), and oxidative stress (11) cause desmin to cleave and aggregate. Recent work identified cleaved desmin fragments as amyloidogenic, capable of inducing misfolding and aggregation of full-length wild-type desmin into amyloid fibrils (12). Amyloid fibrils are misfolded proteins with a characteristic cross $\beta$-sheet structure (13) and are most often associated with neurodegenerative diseases such as Alzheimer's. However, amyloid fibrils also occur in other diseases and parts of the body (14). Amyloids are extremely thermodynamically stable and often resistant to traditional proteolysis (15), leading to insoluble plaque accumulation. While abundant literature focuses on Alzheimer's amyloidogenic A$\beta$, much less is known about desmin's propensity to form amyloids, leaving a significant gap in our understanding of this system and its influence on cardiomyopathy.

*Submitted November 10, 2025, and accepted for publication March 18, 2026.*

*Correspondence: amalpers@udel.edu

Editor: Bernd Reif.

https://doi.org/10.1016/j.bpj.2026.03.038

© 2026 The Author(s). Published by Elsevier Inc. on behalf of Biophysical Society.
This is an open access article under the CC BY license (http://creativecommons.org/licenses/by/4.0/).

Chaperone proteins can refold, sequester, or disaggregate misfolded proteins and inhibit amyloid aggregation (16–18). Chaperone proteins αB-crystallin and heat shock protein 27 (HSP27) constitute at least 3%–5% of cardiac proteins (19). In cardiomyopathies, cells overexpress these chaperones as a cardioprotective measure (20–22). Mutations in αB-crystallin, particularly the R120G missense mutation, result in aggregation of R120G αB-crystallin itself and an increased accumulation of aberrant desmin, demonstrating the crucial role chaperone proteins play in preventing desmin aggregation and associated cardiomyopathies (23–25). Although previous studies demonstrated the interactions of αB-crystallin with full-length desmin (26,27), no research had explored the chaperone-client interaction with pathogenic desmin fragments. Here, we explored how both αB-crystallin and HSP27 could inhibit desmin fragment amyloid aggregation.

HSP27 has high sequence similarity to αB-crystallin, high structural similarity, and belongs to the same small heat shock protein family. HSP27 contains the same three domains as αB-crystallin (N-terminal domain [NTD], C-terminal domain [CTD], and α-crystallin domain [ACD]). HSP27 and αB-crystallin are both ATP-independent chaperones that have β-sheets in the ACD domain that form a dimer interface in the larger oligomer complex, with the NTD and CTD consisting of largely unstructured random coil with some α-helices (28). The current literature model for αB-crystallin's prevention of amyloid formation includes capping of amyloid fibrils through interactions with the β-sheets in the ACD domain (29), although other literature suggests that ACD binding was uncorrelated with chaperone activity without NTD disruption (30). Other literature suggests that the NTD interacts with amorphous clients and that monomers do not interact with the chaperone (29,31–33). HSP27 potentially acts similarly to αB-crystallin (16,34); however, HSP27 is understudied comparatively, even though cells overexpress HSP27 alongside αB-crystallin in heart disease or under stress conditions.

In this study, we performed a rigorous examination of the aggregation kinetics, secondary structure, and morphology of desmin fragment both in the presence and the absence of these two chaperone proteins. While chaperone mutations have long been associated with desmin-related cardiomyopathy and cardiac amyloidosis (3), there are very few papers that look at cleaved desmin fragment without chaperone mutations or additional desmin modifications (such as desmin phosphorylation) (2,9).

By investigating the interactions between chaperone proteins and desmin fragment, we aimed to determine if both αB-crystallin and HSP27 could inhibit desmin amyloid fibril formation. We aimed to discover if both ATP-independent chaperones bind to desmin fragment monomer, amorphous oligomer, and/or amyloid seed, and if so, how much chaperone was required for preventative activity. We wanted to identify if binding sites between chaperone and client were the same as for other amyloidogenic species and what key differences might exist. In this work, we focused on desmin fragment behavior with and without the presence of cardioprotective chaperones αB-crystallin and HSP27. In recent years, the development of pharmacological chaperones, small molecules designed to mimic protein chaperone functions, emerged as a successful strategy for treating protein misfolding disorders (35). Our work could set the stage for using desmin fragment as a model controllable amyloid system for further probing chaperone and/or pharmacological chaperone activity.

## MATERIALS AND METHODS

### Desmin fragment

Lyophilized synthetic desmin fragment (desmin aa 111–143, ELQELNDRFANYIEKVRFLEQQNAALAAEVNRL) was purchased as an HCl salt with guaranteed TFA removal (Genscript, Piscataway, NJ) (12). For experiments at physiological pH, lyophilized desmin fragment was reconstituted to 781 μM in phosphate buffer (20 mM $Na_2HPO_4$, 100 mM NaCl adjusted to pH 7.4 with HCl) and then diluted to final experimental concentrations. For experiments at acidic pH, lyophilized desmin fragment was reconstituted to 448 μM in acidic phosphate buffer (20 mM $Na_2HPO_4$, 100 mM NaCl, adjusted to pH 2.8 with HCl). For seed samples, lyophilized desmin fragment was reconstituted to 256 μM in acidic phosphate buffer and incubated for >20 h at 37°C.

### Circular dichroism

Far-UV circular dichroism (CD) spectra were collected from 300 to 190 nm on a Jasco J-1500 CD spectrometer (Hachioji, Tokyo, Japan) at 37°C with a bandwidth and data interval of 1 nm. Measurements were performed in a demountable quartz cuvette (Starna, Ilford, England). The experimental pathlength (0.0098 cm) of the demountable cuvette was determined using a 0.02 M potassium chromate solution. Three dependent spectral scans were collected, and the data were averaged. Buffer signal was subtracted from all samples. Spectra were smoothed with Savitzky-Golay least-squares fitting in MATLAB. The data were acquired in millidegrees and converted to mean residue ellipticity ($\theta_{MRE}$) (Fig. S1).

### SDS-PAGE monomer assessment

To assess peptide starting monomer content, 256 μM, 640 μM, or 781 μM desmin fragment was cross-linked with 0.25% glutaraldehyde (Electron Microscopy Sciences, Hatfield, PA) for 15 min at 21°C. The reaction was quenched with 25 mM sodium borohydride (Thermo Scientific, Waltham, MA) for 15 min. Fixed samples were then diluted to 64 μM (from 256 μM and 640 μM) or 77 μM (from 781 μM) for clearer band visualization. SDS sample loading buffer (0.05 M Tris-HCl, 0.1 M DTT, 69 mM 2% [w/v] SDS, 1.5 mM bromophenol blue, 1 M glycerol) was added to samples and then heated for 8 min at 85°C. Samples were loaded onto a 4%–20% gradient gel (Bio-Rad, Hercules, CA) and run at 185 V. Gels were fixed with 100 mL of 40% EtOH, 10% acetic acid solution, and gel bands were visualized by Coomassie staining (Bio-Rad, Hercules, CA). ImageJ was used to calculate >77% of desmin fragment as monomeric (Fig. S2).

### Untagged chaperone protein preparation

αB-crystallin or HSP27 (Novus Biologicals, Centennial, CO) was dialyzed into phosphate buffer using 6k MWCO Pur-a-Lyzers (Sigma-Aldrich, St.

Louis, MO). Protein concentration after dialysis was determined by A280 using $\varepsilon(\alpha B) = 13{,}980\ M^{-1}cm^{-1}$ and $\varepsilon(HSP27) = 40{,}450\ M^{-1}cm^{-1}$. Chaperone proteins were aliquoted and stored at −20°C until use.

## Sample preparation for chaperone, seed, and fragment interaction assays

Chaperone proteins were added to 640 μM desmin fragment as listed below for the experiments in Fig. 2 (Table 1); assay volumes were 100 μL.

## ThT assays

Thioflavin T (ThT) assays were performed in a Tecan Spark plate reader (Männedorf, Switzerland) using 384-well black flat-bottom plates (Corning, Corning, NY) with clear polystyrene lids (Corning). Temperature was set to 37°C, and plates were shaken for 15 s every 15 min. The excitation and emission wavelengths were 420 and 485 nm, respectively. All samples contained 20 μM ThT (Thermo Scientific) dye. Buffer+ThT signal was subtracted from all sample spectra. The limit of detection was determined using decreasing concentrations of seed (Figure S3). T50 significance was determined using two-tailed *t* tests (MATLAB function ttest2).

## TEM imaging

Samples were collected directly from the plate before and after fluorescence measurements were performed. Samples were loaded onto carbon-formvar coated copper mesh grids (Electron microscopy) that were glow discharged for 30 s using a PELCO EasiGlow glow discharge unit (Ted Pella, Redding, CA). Samples were left to bind for 1 min and then washed three times with endotoxin free water. Samples were then fixed with 2% glutaraldehyde (Electron Microscopy Sciences) in 0.2 M sodium cacodylate buffer (Electron Microscopy Sciences) for 5 min and then washed three times with endotoxin free water. Grids were negatively stained with 2% uranyl acetate (Ted Pella). Images were acquired on a Talos L120C transmission electron microscope (Thermo Scientific) with an accelerating voltage of 120 kV. Magnification of TEM images ranged from 570,00× (100 nm) to 1,600× (5 μm) (Figs. S4, S5, S6, S7, S8, and S13).

## Fibril length and width analysis

The ImageJ line selection tool was used to measure fibril lengths and corresponding widths from six representative TEM images for each condition. Only fibrils with a length of >60 nm and a length to width ratio of >5 were measured to ensure characterization of fibrils and not amorphous oligomeric assemblies. Up to 10 fibrils per image were measured. If an image contained <10 fibrils, then <10 fibrils/image were measured (figure captions contain *N* measured at each condition for *N* at each condition). For data reporting, fibril lengths that extended beyond the field of view were reported as the measured value and noted. Length and width significance was determined using two-tailed *t* tests.

## FTIR

Samples were collected from the plate before or after fluorescence measurements were performed (separate well in the same plate that did not contain ThT). Samples were flash frozen with liquid nitrogen and lyophilized and then rehydrated with the same volume of $D_2O$ (Thermo Scientific). Fourier transform infrared (FTIR) spectra were collected on an ABB MB100 FTIR spectrometer (Zürich, Switzerland) using 76-μm spacers. All FTIR spectra were baseline corrected and smoothed using Savitzky-Golay least-squares fitting and normalized in MATLAB to their most intense peak in the amide I region.

## Tryptophan fluorescence assays

Intrinsic tryptophan fluorescence assays were performed in a Spark plate reader (Tecan) in 384-well black flat-bottom plates (Corning). The excitation and emission wavelengths were 280 nm and 300–450 nm respectively. Spectra were collected at time 0 h and again after 2 h at 37°C. Buffer signal was subtracted from all samples. Desmin fragment or seed signals were subtracted from the appropriate chaperone+client spectra. Spectra were smoothed with Savitzky-Golay least-squares fitting in MATLAB. Significance was determined using two-tailed *t* tests.

## SDS-PAGE chaperone assessment

To assess if the chaperone was active as a large oligomeric complex when interacting with monomer or seed desmin fragment, 5.46 μM αB-crystallin was incubated with or without 256 μM desmin fragment monomer or 64 μM seed for 2 h at 37°C. Samples were then cross-linked with 0.1% glutaraldehyde (Electron Microscopy Sciences) for 15 min at 21°C. The reaction was quenched with 50 mM sodium borohydride (Thermo Scientific) for 15 min. Gels were run following the same parameters as above (Fig. S9).

## Molecular docking

HDOCK was used to conduct molecular docking. The desmin fragment monomer structure used was based on a truncated AlphaFold model (residues 111–143) of the full-length desmin structure (AlphaFold: AF-P17661-F1). Since no high-resolution structure exists for desmin fragment amyloid, a crude model was generated in PyMOL by building the fragment sequence in a parallel β-sheet conformation. Desmin fragment monomer or amyloid was defined

**TABLE 1 Fragment and chaperone combinations**

| Chaperone protein | Concentration of chaperone (mg/mL) | Concentration of chaperone (μM) | Molar ratio (Desmin fragment:chaperone protein) |
|---|---|---|---|
| αB-crystallin | 0.11 | 5.46 | 117:1 |
| | 0.05 | 2.48 | 258:1 |
| | 0.01 | 0.50 | 1,280:1 |
| | 0.005 | 0.25 | 2,560:1 |
| | 0.001 | 0.05 | 12,800:1 |
| HSP27 | 0.11 | 4.83 | 132:1 |
| | 0.05 | 2.19 | 291:1 |
| | 0.01 | 0.44 | 1,455:1 |
| | 0.005 | 0.22 | 2,909:1 |

For seeded studies, seed and/or chaperone proteins were added to desmin fragments as listed below for the experiments in Figs. 3 and 5 (Table 2).

**TABLE 2 Fragment, seed, and chaperone combinations**

| Concentration of fragment | Concentration of seed | Concentration of chaperone | Molar ratio (Desmin fragment:seed:chaperone protein) |
|---|---|---|---|
| 0.5 mg/mL (128 $\mu$M) | 0.25 mg/mL (64 $\mu$M) | – | 2:1:0 |
| 0.5 mg/mL (128 $\mu$M) | 0.25 mg/mL (64 μM) | 0.11 mg/mL (5.46 $\mu$M) $\alpha$B-crystallin | 23:12:1 |
| 0.5 mg/mL (128 $\mu$M) | 0.25 mg/mL (64 μM) | 0.11 mg/mL (4.83 $\mu$M) HSP27 | 27:13:1 |
| – | 0.25 mg/mL (64 $\mu$M) | – | 0:1:0 |
| – | 0.25 mg/mL (64 μM) | 0.11 mg/mL (5.46 $\mu$M) $\alpha$B-crystallin | 0:12:1 |
| – | 0.25 mg/mL (64 μM) | 0.11 mg/mL (4.83 $\mu$M) HSP27 | 0:13:1 |
| – | – | 0.11 mg/mL (5.46 $\mu$M) $\alpha$B-crystallin | 0:0:1 |
| – | – | 0.11 mg/mL (4.83 $\mu$M) HSP27 | 0:0:1 |

as the ligand, and $\alpha$B-crystallin 24-mer (PDB: 2YGD) and HSP27 24-mer (PDB: 6DV5) were defined as the receptors. Docking was performed on the HDOCK server using the default parameters (Figs. S10 and S11).

## Cross-linking mass spectrometry

Desmin fragment seed was sonicated for 15 min prior to the experiment. All steps were performed at room temperature unless otherwise noted. Samples were incubated for 2 h, then DSSO (Sigma-Aldrich) in DMSO (freshly prepared) was added to a final concentration of 0.1 mM, and samples were incubated for 45 min. For control samples, the same volume of DMSO was added without DSSO. Tris-HCl buffer (pH 8.0, adjusted with NaOH) was added to a final concentration of 50 mM, and samples were incubated for 15 min. Cold acetone was added to a final concentration of 80% (v/v). Samples were incubated on ice for 1 h and then centrifuged for 10 min at 20k $\times$ $g$. The supernatant was discarded and the precipitate dried for 1 h. Samples were resuspended in 50 mM sodium bicarbonate buffer, 8 M urea, and then 50 mM triethylammonium bicarbonate buffer was added to lower the concentration of urea to 500 mM. DTT was added to a final concentration of 5 mM, and samples were incubated at 56°C for 20 min. Iodoacetamide was then added to a final concentration of 15 mM, and samples were incubated for 15 min in the dark. DTT was added again to a final concentration of 10 mM, and samples were incubated at 56°C for 20 min. Trypsin (Promega, Madison, WI) was added to samples at a ratio of 1:20 (w/w) and incubated at 37°C for 20 h. Formic acid was added to the samples at a final concentration of 1% (v/v). Samples were then desalted using Pierce C18 spin columns (Thermo Scientific), lyophilized, and stored at −20°C. All conditions were tested in independent triplicates.

For LC-MS/MS analysis, we utilized the Ultimate 3000 RSLCnano system and Eclipse Orbitrap mass spectrometer (Thermo Scientific). Peptides were first resuspended in LC solvent A (0.1% formic acid in water) and then loaded onto a trap column (PepMap100 C18, 300 $\mu$m $\times$ 2 mm, 5 $\mu$m; Thermo Scientific) followed by separation on an analytical column (PepMap100 C18, 50 cm length, 75 $\mu$m i.d., 3 $\mu$m; Thermo Scientific, Waltham, MA). For LC separation, a linear gradient flowing at 250 nL/min was applied from 1% solvent B (0.1% formic acid in acetonitrile) to 30% solvent B (0.1% formic acid in acetonitrile) over 100 min followed by a sharp gradient to 32% solvent B over 10 min. The column was washed with 85% solvent B for 5 min and equilibrated with solvent A for 15 min. For MS1 analysis, the detector type was set to Orbitrap under resolution 120,000; the scan range was m/z 375–1,600; the AGC target and maximum injection time mode were set to standard and Auto, respectively. For ddMS2 analysis, the isolation mode was Quadrupole, the activation type was CID, and the detector type was Orbitrap under resolution of 60,000. The AGC target was set to standard, and the maximum injection time was 118 ms. For targeted mass difference, DSSO and delta mass 31.97 Da were defined. For ddMS3 analysis, the activation type was HCD, and the detector type was Orbitrap with resolution of 15,000. The AGC target was 300%, and maximum injection time was 22 ms.

Raw files were analyzed using Proteome Discoverer 2.5 with XlinkK 2.5 node. A MS2-MS3 workflow was utilized with mass tolerance of 10 ppm (MS1), 20 ppm (MS2), and 0.5 Da (MS3). Digestion was set to trypsin (cleavage after K/R) with 4 missed cleavages allowed, and the minimum peptide length was 4 amino acids. Static modifications of carbamidomethylation were set for cysteines, and dynamic modifications of oxidation for methionine and DSSO for lysines, serines, threonines, and tyrosines were set. The XlinkX/PD Validator node was used for cross-linked peptide validation with a 1% false discovery rate. All cross-links were examined manually, and an XlinkX Score cutoff of 50 and a ΔXlinkX Score cutoff of 10 were considered as indicative of true cross-links (Tables S1 and S2; Figs. S10, S11, and S12).

## His-tagged $\alpha$B-crystallin expression and purification

Human $\alpha$B-crystallin gene was cloned into a pET28a(+) expression vector. The resulting $\alpha$B construct had an N terminus 6xHis tag and a TEV protease cleavage site and was transformed into *E. coli* strain BL21 (DE3). Colonies were transferred to LB medium (10 g/L NaCl, 10 g/L tryptone, 5 g/L yeast extract, and 50 $\mu$g/mL kanamycin) and incubated at 37°C, 250 rpm shaking until OD600 reached between 0.6 and 0.8 AU. Expression was induced with 2 mM IPTG and incubated at 18°C, 250 rpm overnight. Cells were harvested by centrifugation 6k rpm for 30 min at 4°C. Pellets were stored at −80°C. For purification, pellets were resuspended in lysis buffer (50 mM $Na_2HPO_4$, 150 mM NaCl, 5 mM imidazole, pH 7.7), and cells were lysed using a sonicator probe (Branson Ultrasonics, Brookfield, CT) on ice. Cells were centrifuged at 13k rpm for 45 min at 4°C. The rest of the purification was performed at 4°C. Ni-NTA resin slurry (50% slurry in 20% ethanol) was added into a gravity-flow column and was equilibrated with lysis buffer. Supernatant was added to equilibrated resin, incubated with shaking at 4°C for 1 h, and then allowed to flow through the column. The column was then washed with wash buffer (50 mM $Na_2HPO_4$, 500 mM NaCl, 50 mM imidazole, pH 7.7). $\alpha$B was eluded by a stepwise imidazole gradient (50 mM $Na_2HPO_4$, 150 mM NaCl, 50–500 mM imidazole pH 7.7), with $\alpha$B eluding at a concentration of 200 mM imidazole. $\alpha$B fractions were pooled and concentrated with 10-kDa MWCO Amicon centrifuge filters (MilliporeSigma, Burlington, MA). Concentrated samples were dialyzed into phosphate buffer using 2-kDa MWCO Slide-a-Lyzers (Thermo Scientific). Aliquots were kept at −80°C after flash-freezing with liquid nitrogen. $\alpha$B samples were >98% pure as judged by SDS-PAGE. The molecular weight of the protein was confirmed using intact protein MS. Morphology of his-tagged $\alpha$B was determined using TEM and dynamic light scattering (DLS) and found to be similar to wild-type (WT) $\alpha$B. Activity was assessed with a ThT assay and was also found to be similar to WT $\alpha$B.

## Bio-layer interferometry

Bio-layer interferometry (BLI) assays were performed on an Octet RH16 (Sartorius, Göttingen, Germany) in 384-well black flat-bottom plates (Greiner Bio-One, Monroe, NC). Anti-Penta-HIS (HIS1K) biosensors (Sartorius) were hydrated in 200 $\mu$L of phosphate buffer for >20 min before assays. All assays were performed at 25°C with the orbital shake speed of 1,000 rpm and a well

volume of 80 $\mu$L. Biosensors were first baselined for 60 s in phosphate buffer followed by loading of 100 nM his-tagged $\alpha$B-crystallin for 180 s. After loading, an additional baseline in phosphate buffer was performed to remove any unbound $\alpha$B-crystallin. The biosensors were then submerged in wells containing different concentrations of desmin fragment monomer or seed (10 $\mu$M, 5 $\mu$M, 1 $\mu$M, and 0.5 $\mu$M) for 600 s followed by 650 s of dissociation time in phosphate buffer. Biosensors were also dipped in wells containing just phosphate buffer to allow for single reference subtraction. Data was processed in Octet Studio. Buffer signal containing ligand, but no analyte, was subtracted from all samples, and y-axes were aligned to the averaged baseline signal from 55 to 60 s. An inter-step correction was applied at the end of the dissociation step to correct for buffer mismatch, and a default Savitzky-Golay filtering was applied to smooth the data. Data were best fit to a heterogeneous ligand model (Fig. S14).

## Dynamic light scattering

DLS data were collected at time 0 h, or after 2 h or 19 h of incubation at 37°C. DLS measurements were performed using a Zetasizer Nano ZS (Malvern Instruments, Worcestershire, UK) with a back-scattering angle of 173° and a fixed wavelength of 532 nm. Three dependent measurements (10 acquisitions of 10 s each) were collected for each independent preparation, and all sample conditions were prepared in duplicates ($n = 6$). Autocorrelation functions were converted into particle-size distributions using the general resolution algorithm provided with the Zetasizer Nano ZS software (Fig. S15).

## Estimation of concentration of HSP27 and $\alpha$B-crystallin in heart tissue

The unstressed heart contains 3%–5% $\alpha$B-crystallin out of the total protein soluble content (36) and less than 1% HSP27 (but enough for antibody staining) (37). Adult human hearts contain between 0.6 $\mu$g/mg to 2.6 $\mu$g/mg HSP27 per weight of proteins (38). The clinically accepted value of myocardial tissue density is 1.055 g/mL (39), and 20% of muscle tissue is protein (40). The mg/mL of chaperone proteins in healthy heart tissue was estimated as follows:

$$\frac{2.6\ \mu g\ HSP27\ (maximum)}{mg\ protein} * \frac{1.055\ g\ tissue}{1\ mL\ tissue} * 0.2\ (protein\ in\ tissue) * \frac{1000\ mg}{1\ g} = 0.55\ mg\ HSP27 \Big/ mL\ tissue \quad (1)$$

Knowing this is under 1% and estimating it is as much as 0.5%, we estimated that $\alpha$B-crystallin is $\sim$3 mg/mL in heart tissue. In this work, the maximum chaperone concentration is 0.11 mg/mL, or 5.46 $\mu$M, far below the tissue estimate.

## RESULTS

### Desmin fragment forms amyloid at physiological pH

Desmin fragment (desmin residues 111–143) was previously identified (12) as amyloidogenic at acidic pH (Figs. 1 *A*; S1). For this work, we focused on fragment behavior at physiological pH to mimic conditions more relevant to cardiomyopathy (41) after verifying that our synthetic fragment starts predominantly as monomeric (Fig. S2). Thioflavin T (ThT) fluorescence showed typical sigmoidal aggregation kinetics for 128 $\mu$M to 768 $\mu$M (Fig. 1 *B*). Fibrilization half-time (T50) increased with decreasing desmin concentration. We did not observe aggregation for the 64 $\mu$M sample. The amyloid limit of detection in our ThT assay was 15 $\mu$M (0.06 mg/mL, Fig. S3). For all kinetic experiments, we used pooled peptide lots that aggregated at the same timescale to account for synthetic peptide lot-to-lot variability (42).

To visualize aggregates, we measured transmission electron microscopy (TEM) images (Fig. 1 *C*, left). TEM images show, for 128 $\mu$M and above, all samples at 50 h contained amyloid fibrils. These fibrils exhibit diverse morphologies, including twisted and straight structures (Fig. S4). In contrast, we only observed amorphous aggregates for the 64-$\mu$M sample (Fig. 1 *C*, right). These TEM images confirm our ThT assay observations.

We used Fourier-transform infrared spectroscopy (FTIR) to identify desmin secondary structure before and after aggregation (Fig. 1 *D*). The FTIR spectra show the desmin fragment began in random coil structure (amide I vibrational mode peak at 1,645 $cm^{-1}$) across all concentrations measured. The fragment was likely in a mixture of $\alpha$-helix (Fig. S1) and random coil structure immediately after reconstitution. After 50 h, the 256-$\mu$M sample shifted to 1,624 $cm^{-1}$ (Fig. 1 *D*, dark green), indicating amyloid $\beta$-sheet secondary structure, with growth of a smaller peak at 1,670 $cm^{-1}$, indicating unfolding (43). The 64-$\mu$M sample remains centered at 1,645 $cm^{-1}$, predominantly remaining in its initial secondary structure, even in amorphous aggregates (Fig. 1 *D*, light green).

For the 128-$\mu$M sample, we initially expected a similar spectrum to the 256-$\mu$M sample after 50 h based on only the ThT and TEM data; however, we instead observed an amide I peak centered at 1,635 $cm^{-1}$, accompanied by an equally large 1,670-$cm^{-1}$ unfolding peak. These FTIR signatures indicate a mixture of both folded and unfolded secondary structures. The 1,635-$cm^{-1}$ peak indicates a less delocalized, more native-like $\beta$-sheet structure compared with the 1,624-$cm^{-1}$ amyloid $\beta$-sheet structure, and the broader peak indicates a larger mixture of aggregated secondary structures. As FTIR is a more specific technique for protein secondary structures than ThT assays or TEM imaging, it makes sense that we observed this difference with FTIR. Together, our ThT, TEM, and FTIR experiments identify a concentration-dependent transition in desmin fragment's aggregation behavior with high concentrations driving faster amyloid formation at physiological pH.

### Chaperone proteins, $\alpha$B-crystallin and HSP27, inhibit or delay desmin fragment amyloid formation

Chaperone proteins $\alpha$B-crystallin and HSP27 were shown to inhibit amyloid formation of neurodegenerative

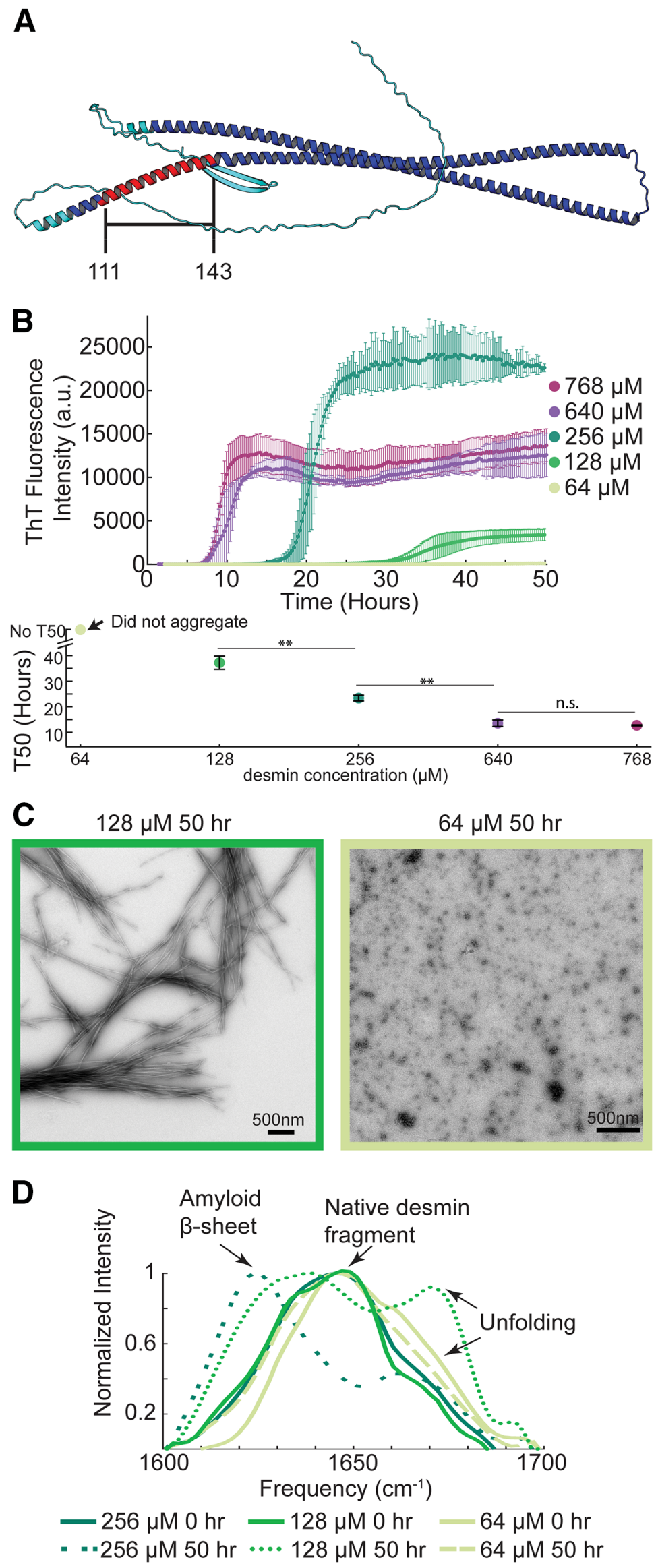


FIGURE 1 Desmin fragment amyloid can form at physiological pH and at 37°C. (*A*) Alphafold prediction of full-length desmin with fragment (AA 111–143) region highlighted in red (AlphaFold: AF-P17661-F1). N-terminal and C-terminal regions are colored in cyan with rod domain colored in blue. (*B*) ThT fluorescence of desmin fragment concentrations from 64 $\mu$M to 768 $\mu$M. Values averaged for T50s ($n = 3$) with 95% confidence interval shown. n.s.: not significantly different, **: $p < 0.005$. (*C*) TEM images of desmin at 128 $\mu$M and 64 $\mu$M after 50 h. Scale bars represent 500 nm. (*D*) Normalized FTIR spectra of desmin fragment at 256 $\mu$M, 128 $\mu$M, and 64 $\mu$M at time points 0 and 50 h.

disease-relevant A$\beta$ and $\alpha$-synuclein (16–18,44–46). Moreover, $\alpha$B-crystallin was shown to interact with full-length desmin (26,27,47). However, it was unknown if $\alpha$B-crystallin or HSP27 could suppress desmin fragment amyloid formation, nor to what extent. Thus to determine chaperone impact on desmin fragment amyloid formation, we used ThT, TEM, and FTIR assays of desmin fragment with and without chaperone present.

We observed complete desmin fragment amyloid inhibition for 0.05 to 5.46 $\mu$M $\alpha$B-crystallin (Fig. 2 *A*, dark pink, 1:117 chaperone:desmin fragment). At an extremely low concentration ratio of $\alpha$B-crystallin to desmin fragment (1:12,800), we did not observe complete inhibition, but instead delayed eventual amyloid formation Fig. 2 *A*, peach). While there are few literature examples of small chaperone to client ratios (31,48), here, we observed an exceptionally small ratio of chaperone present can still successfully delay amyloid formation.

To control for nonspecific interactions, we used a functionally unrelated protein in place of the chaperone. We performed this experiment to ensure that observed inhibition or delay in desmin fragment amyloid formation can be attributed to specific chaperone activity and not just to interactions of the fragment with the nonspecific protein instead of a fragment with another aggregating fragment. We incubated bovine serum albumin (BSA) with desmin fragment at the same molar ratio as chaperone (1:117) and then monitored fibril formation with ThT fluorescence (Fig. 2 *A*, light blue). Desmin fragment+BSA formed amyloid but with a significant T50 delay as compared with desmin alone, (T50 desmin fragment+BSA: 16.00 $\pm$ 1.04 h, T50 desmin fragment alone: 10.06 $\pm$ 1.24 h, Fig. 2 *A*, purple versus light blue). In contrast, we observed complete inhibition (no aggregation) in the presence of chaperone (1:117 $\alpha$B-crystallin to desmin fragment). It was only when the chaperone ratio decreases to 1:12,800 for $\alpha$B-crystallin that we started to see a delay in aggregation instead of complete inhibition. Because the delay in the presence of small amounts of chaperone was still significantly larger (slower T50) than the nonspecific interactions with BSA, specific chaperone interactions must influence and prevent desmin fragment aggregation.

Compared with $\alpha$B-crystallin, HSP27 inhibited aggregation at a narrower range (0.44–4.83 $\mu$M). The highest concentration of HSP27 (4.83 $\mu$M) fully inhibited desmin fragment amyloid formation (Fig. 2 *B*, dark orange, 1:132 chaperone:desmin fragment). HSP27 only delayed aggregation instead of complete inhibition at a higher concentration ratio than $\alpha$B-crystallin (1:2,909), but it was still significantly more effective as a specific chaperone than the nonspecific crowding interactions with BSA (Fig. 2 *B*, yellow versus light blue). Overall, these kinetic data suggest that both $\alpha$B-crystallin and HSP27 have specific protective activity toward desmin fragment and can delay amyloid aggregation at even extremely low molar ratios. However, based on concentration ratios and T50s, HSP27 demonstrates weaker chaperone activity than $\alpha$B-crystallin.

Next, we probed desmin fragment secondary structure using FTIR with and without $\alpha$B-crystallin or HSP27 present. At high concentrations of chaperone, we did not observe a shift of the 1,645 $cm^{-1}$ peak (Fig. 2. *C–D* purple versus dark pink and dark orange). For the high concentration HSP27 sample, we observe slight desmin

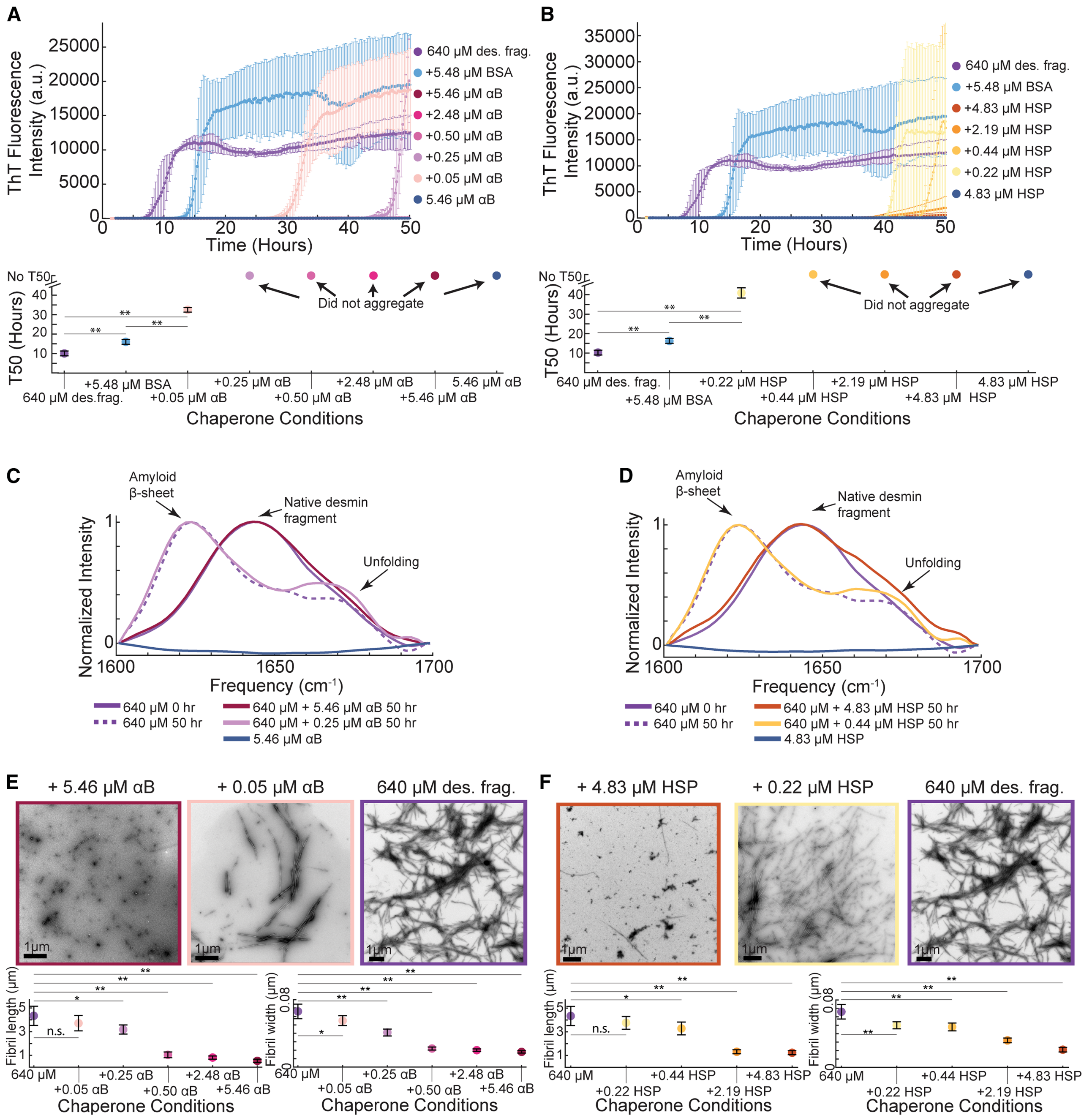


FIGURE 2 Chaperone proteins $\alpha$B-crystallin and HSP27 inhibit or delay desmin fragment amyloid formation at physiological pH and 37°C. (*A*) ThT fluorescence of $\alpha$B-crystallin from 0.05 to 5.46 $\mu$M in the presence of 640 $\mu$M desmin fragment. Control samples include desmin fragment+BSA and 5.46 $\mu$M $\alpha$B-crystallin alone. (*B*) ThT fluorescence of HSP27 from 0.22 to 4.83 $\mu$M in the presence of 640 $\mu$M desmin fragment with the same controls as in (*A*). For (*A*) and (*B*), values were averaged for T50s ($n = 3$) with 95% confidence interval shown. **: $p < 0.005$. Normalized FTIR of desmin fragment at 640 $\mu$M alone at 0 h and after 50 h or in the presence of 5.46 or 0.25 $\mu$M $\alpha$B-crystallin (*C*) or 4.83 or 0.44 $\mu$M HSP27 (*D*) after 50 h. TEM images of desmin fragment with $\alpha$B-crystallin (*E*) or HSP27 (*F*) after 50 h with average fibril length and width for each chaperone condition. For (*E*) and (*F*), scale bars represent 1 $\mu$m. 95% confidence interval shown. n.s.: not significantly different, *: $p < 0.05$, **: $p < 0.005$. $n = 60$ fibrils measured for length and width for all TEM images except +5.46 $\alpha$B, +2.48 $\alpha$B, +4.83 HSP, and +0.22 HSP ($n = 58$, 51, 55, and 59, respectively).

unfolding (increase in 1,670 $cm^{-1}$ intensity), which we do not observe in the presence of $\alpha$B-crystallin. At low concentrations of chaperone, for which we cannot calculate a T50, but ThT fluorescence increased, we observe a distinct shift to 1,624 $cm^{-1}$, indicating amyloid $\beta$-sheet secondary structure formation (Fig. 2, *C–D*, light purple and yellow).

To visualize aggregates in the presence of chaperones, we measured TEM images (Figs. 2, *E* and *F*; S5 and S6). At all chaperone concentrations, TEM images showed a change

from amorphous aggregates at 0 h to amyloid fibrils at 50 h, with an increase in fibril abundance with decreasing chaperone concentrations (Fig. 2, *E–F*). However, our ThT and FTIR measurements do not indicate fibril formation after 50 h in the presence of 5.46 $\mu$M $\alpha$B-crystallin or 4.83 $\mu$M HSP27. Thus, these fibrils identified by TEM are likely at too low of a concentration compared with the overall desmin fragment structures present to impact FTIR and ThT measurements (ThT fibril limit of detection determined to be 15 $\mu$M, 0.06 mg/mL, Fig. S3).

We measured fibril length and width in the TEM images. We only measured fibrils with a length >60 nm and a length to width ratio of >5 to ensure we characterize only fibrils and not amorphous oligomeric assemblies. For the concentrations that delayed but did not prevent aggregation (0.05 $\mu$M $\alpha$B, 0.22 $\mu$M HSP27), fibril length was not statistically different from desmin fragment alone. However, for the next lowest concentration (0.25 $\mu$M $\alpha$B, 0.44 $\mu$M HSP27), where fluorescence increased, but no T50 was determined and FTIR indicated amyloid $\beta$-sheets, fibril length was significantly shorter from desmin fragment alone. All other chaperone concentrations also resulted in significantly shorter fibril lengths (Fig. 2, *E–F*). In contrast, for fibril widths, we observed thinner fibrils at all concentrations of $\alpha$B-crystallin or HSP27 compared with desmin fragment alone (Fig. 2, *E–F*). Thinner fibrils suggest that while low chaperone concentrations do not prevent aggregation, both chaperones alter the aggregation pathway and influence the templating process during fibril assembly. Overall, as chaperone concentration decreased, fibril abundance, length, and width increased.

For the highest three concentrations, desmin fragment+HSP27 fibrils were significantly longer than matched conditions for desmin fragment+$\alpha$B-crystallin, and for the lowest three concentrations, desmin fragment+HSP27 fibrils were significantly wider than for desmin fragment+$\alpha$B (Fig. 2, *E–F*). This finding suggested $\alpha$B-crystallin was a more effective chaperone for desmin fragment than HSP27. While both are found in the heart, HSP27 at ~0.5 mg/mL and $\alpha$B at ~3 mg/mL (materials and methods), $\alpha$B-crystallin interacts with many clients, whereas HSP27 interacts primarily with actin (49,50). Therefore, structural and sequence differences between desmin and actin may explain why $\alpha$B-crystallin was a better chaperone than HSP27 for desmin fragment in our experiments. Collectively, our data indicated both chaperones acted protectively for the desmin fragment. Both $\alpha$B-crystallin and HSP27 act as molecular chaperones for desmin fragment, inhibiting or significantly delaying desmin fragment amyloid formation in a concentration-dependent manner.

## $\alpha$B-crystallin and HSP27 inhibit or delay seeded desmin fragment amyloid formation

Seeding experiments are common in the amyloid field because these experiments mimic conditions that allow aggregates to spread through formerly healthy tissue (51). Seeding experiments consist of adding seed, or fully formed amyloid fibril, into an unaggregated solution of the same peptide to induce aggregates to form with the same amyloid $\beta$-sheet secondary structure (also known as templating). Seed is often generated using acidic conditions rather than high concentration. Previously, desmin fragment seed was shown to induce full-length desmin protein to misfold and form amyloid (12).

Here, we showed adding seed to 128 $\mu$M desmin decreases T50 compared with desmin without seed (Fig. 3 *A*). In contrast, we observe no significant difference for the T50 between desmin and a control of desmin+pH 2.8 volume instead of seed (green versus beige, Fig. 3 *A*). Thus, the seed must template the desmin for amyloid aggregation. Next, we measured monomer+seed+chaperone to determine if $\alpha$B-crystallin or HSP27 could prevent seed from templating monomeric desmin fragment amyloid formation. ThT data indicate both chaperones can individually inhibit amyloid aggregation (Fig. 3 *A*). We compared this to control samples that do not show any aggregation (seed alone, seed+chaperones, chaperones+pH 2.8 volume) (Fig. 3 *A*).

The TEM images also showed that seed templates desmin fragment monomer (Figs. 3 *B*; S7). From 1.5 to 50 h, we measured lengths of fibrils for monomer+seed and seed alone. We observed no changes in isolated seed length over time, but significant increases in monomer+seed length (Fig. 3 *B*, bottom left). When comparing measured fibril widths, we observed an increase in isolated seed width and a larger increase in monomer+seed width over time (Fig. 3 *B*, bottom right). In contrast, for monomer+seed+-chaperone, we observed no change in the seed length or width over time when in the presence of either chaperone. The TEM images and measurements showed that only the initial seed structures present at time 1.5 h remain after 50 h in the presence of chaperone. Thus, the chaperones did not disaggregate the seed, but they interacted with seed to prevent width changes. The chaperones also interacted with monomer, since otherwise, monomer alone would aggregate under these conditions. TEM images of our control experiments verified that the chaperone+seed and chaperone+pH 2.8 volume samples do not show any aggregation (Fig. S8).

Next, we probed desmin fragment secondary structure changes in the presence of seed using FTIR. After 50 h of monomer+seed incubation, the main amide I mode shifted from native random coil structure (1,645 $cm^{-1}$) to amyloid $\beta$-sheet structure (1,620 $cm^{-1}$) and an unfolding peak appeared (1,670 $cm^{-1}$, Fig. 3 *C*). For monomer+seed+chaperone, the main peak did not shift from native random coil; however, we observed slight unfolding (increase in 1,670 $cm^{-1}$ intensity) for both chaperones. Collectively, our data indicated a protective effect of both chaperones on the desmin fragment in the presence of seed. Both

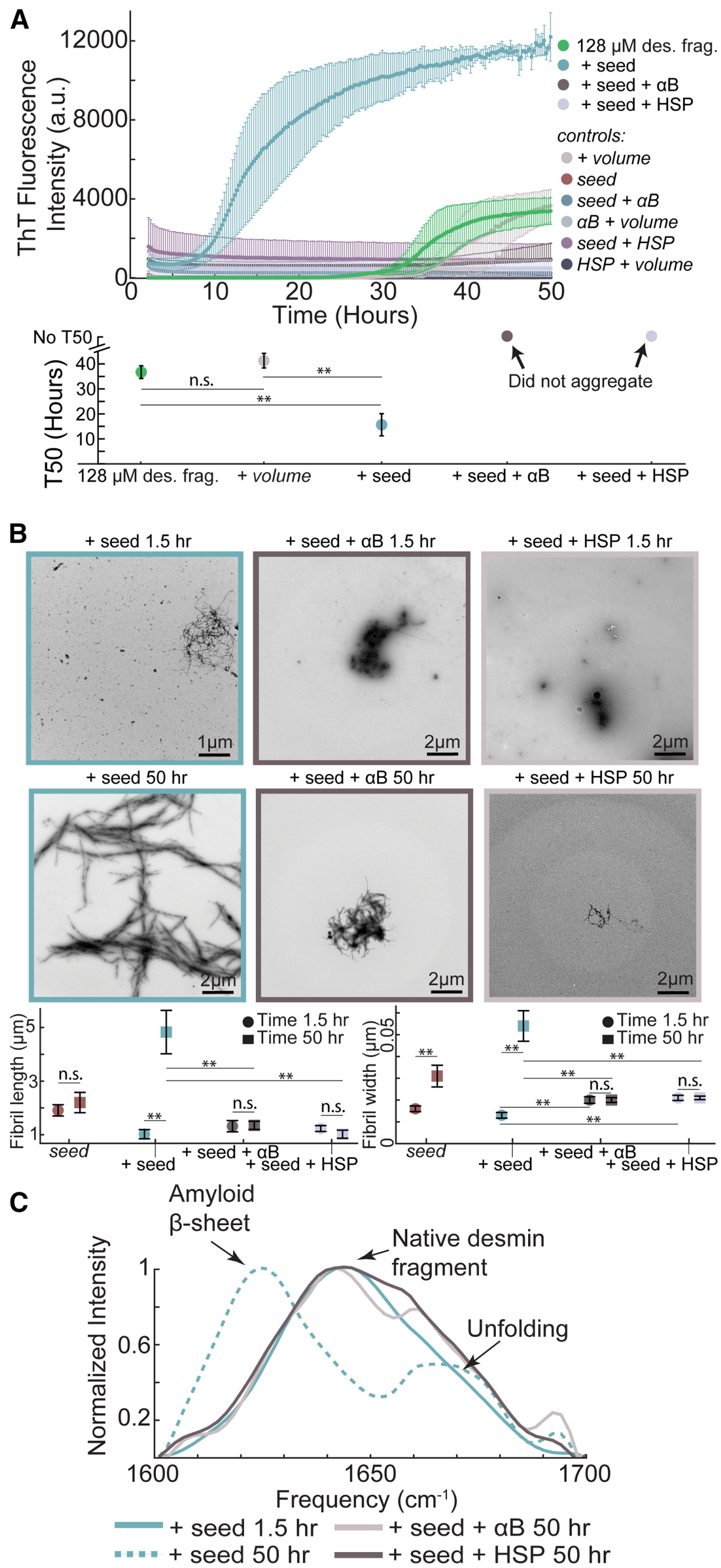


FIGURE 3 Chaperone proteins $\alpha$B-crystallin and HSP27 inhibit seeded desmin fragment amyloid formation. (*A*) ThT fluorescence of desmin fragment monomer, monomer+seed, and monomer+seed+chaperone at concentrations of 128 $\mu$M monomer, 64 $\mu$M seed, 5.46 $\mu$M $\alpha$B-crystallin, and 4.83 $\mu$M HSP27. Control samples included monomer+pH 2.8 volume, seed alone, seed+chaperone alone, and chaperone+pH 2.8 volume. Values averaged for T50s ($n = 3$) with 95% confidence interval shown. n.s.: not significantly different, **: $p < 0.005$. (*B*) TEM images were collected and fibrils measured after 1.5 h and 50 h. Scale bars represent 2 $\mu$m except for monomer+seed 1.5 h (1 $\mu$m). 95% confidence interval shown. n.s.: not significantly different, **: $p < 0.005$. The number of fibrils measured for length and width were: $n = 60, 28, 44, 60$ for 1.5 h seed, monomer+seed, monomer+seed+$\alpha$B, and monomer+seed+HSP, respectively. $n = 60, 58, 40, 56$ for 50 h seed, monomer+seed, monomer+seed+$\alpha$B, and monomer+seed+HSP, respectively. (*C*) Normalized FTIR spectra of desmin+seed and desmin+seed+chaperone after 50 h.

$\alpha$B-crystallin and HSP27 individually interacted with both seed and monomer to inhibit further amyloid formation.

## Chaperone binding sites differ for monomeric desmin fragment and amyloid fibril desmin fragment

After we determined from the above experiments that both chaperones could prevent desmin fragment amyloid aggregation, we next wanted to identify where in the peptide/chaperone complex key chaperone activity might occur. To better understand chaperone structural changes during protective activity, we measured changes in intrinsic fluorescence for the chaperone tryptophan residues. Desmin fragment does not contain any tryptophan; thus, intrinsic fluorescence monitored chaperone proteins themselves, and any changes to fluorescence emission could indicate chaperone interactions with desmin fragment, or they could indicate rearrangement of the chaperone oligomer upon interactions with desmin fragment. Other studies suggest that when $\alpha$B-crystallin was phosphorylated or in the presence of other clients like $\alpha$-synuclein fibrils, it could form smaller active species, such as dimers, hexamers, or 12-mers that would impact the intrinsic fluorescence of the chaperone's NTD (28,31,52).

When we combined desmin fragment monomer with chaperone, we initially observed a significant redshift for both monomer+$\alpha$B-crystallin and monomer+HSP27 compared with chaperone without client present (Fig. 4, *A–B*). A redshift indicates tryptophan's exposure to a more hydrophilic environment (53), and here, structural changes could indicate recognition and binding to the desmin fragment monomer. In contrast, for chaperone+seed (no monomer added), we do not observe peak shifts changes for either $\alpha$B-crystallin or HSP27. Similar to our ThT assays, here, we performed a control experiment to verify that adding pH 2.8 volume did not cause a shift (Fig. 4 *A–B*).

After 2 h of co-incubation, we remeasured intrinsic fluorescence. We observed no significant change in peak wavelength for chaperone alone or with pH 2.8 volume, indicating that both $\alpha$B-crystallin and HSP27 structures did not change without client present (Fig. 4 *A* and *B*). We observed no significant changes in wavelength for either chaperone+seed. However, we did observe a significant blueshift in intrinsic fluorescence of $\alpha$B-crystallin+monomer at 2 h, indicating further arrangement and a potential change in interactions between chaperone and monomer. A blueshift indicates tryptophan burial to a more hydrophobic environment (53). We also observed this blueshift for HSP27+monomer at 2 h, but while significantly different

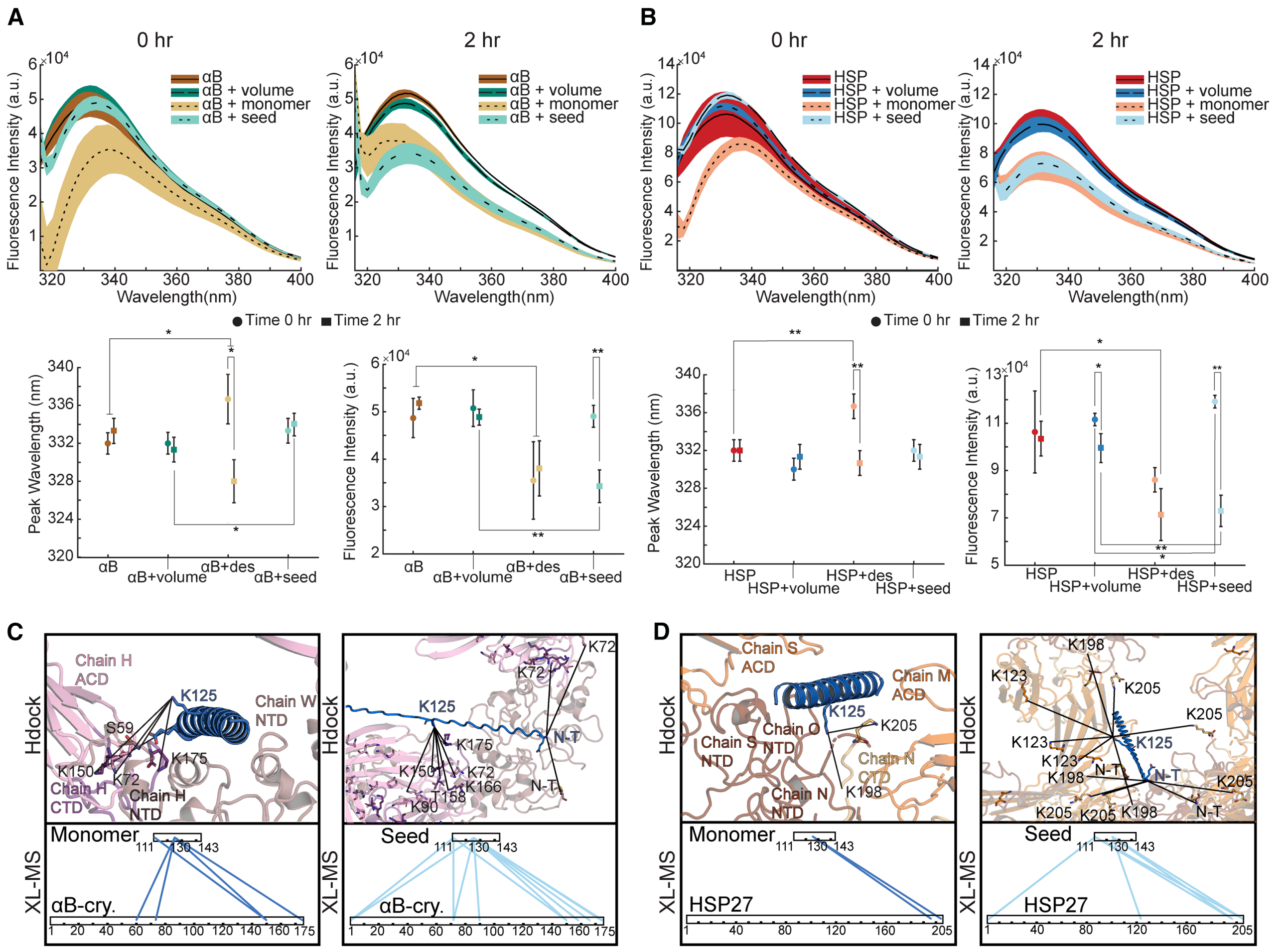


FIGURE 4 Chaperone proteins αB-crystallin and HSP27 interact differently with desmin fragment monomer and desmin fragment seed. (*A*) Interactions of desmin fragment with αB-crystallin or (B) HSP27 monitored by intrinsic tryptophan fluorescence. Average fluorescent spectra ($n = 3$) (black lines) with standard deviation (colored shading) for chaperone alone or with monomer, seed, or pH 2.8 volume control, measured immediately after preparation (0 h) or 2 h of incubation. Peak wavelength and maximum fluorescence intensity compared at 0 h and after 2 h of incubation. Values averaged for T50s ($n = 3$) with 95% confidence interval are shown. *: $p < 0.05$, **: $p < 0.005$, remaining not significantly different (not shown). (*C*) Top-scoring HDOCK model of desmin fragment monomer or seed bound to αB-crystallin (NTD: brown, ACD: pink, CTD: purple) with XL-MS identified cross-links overlaid as lines onto predicted complexes. Graphic of XL-MS identified cross-linked residues between monomer or seed and αB-crystallin. (*D*) Top-scoring HDOCK model of desmin fragment monomer or seed bound to HSP27 (NTD: red-brown, ACD: orange, CTD: tan) with XL-MS identified cross-links overlaid as lines onto predicted complexes. Graphic of XL-MS identified cross-linked residues between monomer or seed and HSP27. For (*A*)–(*D*), samples contained 5.46 μM αB-crystallin, 4.83 μM HSP27, 256 μM desmin fragment monomer, and/or 128 μM seed.

from HSP27+monomer at 0 h, the peak wavelength of HSP27+monomer at 2 h was not significantly different from HSP27 alone at 2 h.

αB-crystallin contains two tryptophan residues, both located in its NTD, while HSP27 contains six tryptophan residues, five of which are located in the NTD. The wavelength shifts we observed for each chaperone+monomer suggest that tryptophan residues directly interacted with desmin fragment monomer, or that tryptophan residues indicated chaperone oligomeric rearrangement when exposed to monomer. We performed a cross-linking gel study to investigate the active interacting form of the chaperones. Our cross-linking gel suggested that αB-crystallin could potentially interact with desmin monomer as a ≥12-mer, although smaller active oligomer assemblies of chaperone with large amounts of monomer cannot be entirely ruled out (Fig. S9). Observed wavelength shifts could imply that the chaperone+monomer interactions occurred at or in close proximity to the chaperone NTDs. This observation was supported by previous literature indicating that αB-crystallin's NTD interacted protectively with amorphous protein aggregates (29), and NTDs of both αB-crystallin and HSP27 play a role in delaying apoC-II amyloid nucleation (18). Alternatively, the shifts could imply a change in chaperone oligomerization at the NTD oligomer interface in the presence of monomer only, distinct from chaperone in the presence of seed (28,31).

Alongside wavelength shifts, we also monitored changes to fluorescence intensity, as changes in intensity could also indicate structural rearrangement. HSP27+pH 2.8 volume intensity changed from 0 to 2 h, which could arise from

subtle structural rearrangement of HSP27 or increase in oligomerization for unphosphorylated chaperones near 36°C (54,55), but both HSP27+pH 2.8 volume intensities were within the error of the HSP27 alone samples. We observed a significant decrease in intensity for both chaperones+seed from 0 to 2 h, indicating binding of the chaperone to seed over time. Chaperone binding to seed could decrease intensity by placing tryptophan near desmin seed amino acids that naturally quench tryptophan's fluorescence (56). Because we observed a decrease but no wavelength shift, seed-chaperone binding may not result in structural rearrangements that alter tryptophan residues' polar environment. Overall, we observed different fluorescence changes for seed versus monomer. Our intrinsic fluorescence data could indicate desmin monomer binds to different chaperone sites than seed, or it could alternatively indicate that the oligomeric state of the chaperone was different when binding to desmin monomer versus seed.

Next, to identify specific residues that could mediate chaperone and desmin fragment interactions, we modeled monomer+chaperone complexes using the HDOCK protein docking server (57). The top-scoring Hdocking models predicted desmin fragment monomer binding not at the canonical ACD dimer interface of the chaperone, but at the junction where NTDs from two chaperone chains belonging to adjacent hexameric rings converge (Fig. 4, C and *D*, top left; Figures S10 and S11). This positioning indicated a potential inter-ring interaction site that could contribute to monomer client recognition and binding. While no experimentally determined or predicted desmin fragment amyloid structure was available, we could estimate docking predictions with a forced amyloid $\beta$-sheet structure for desmin fragment seed (Fig. 4 *C* and *D*, top right; Figs. S10 and S11). Due to this forced amyloid structural estimate, these docking predictions should not inform direct comparisons for binding sites for monomer versus seed without additional experiments.

To experimentally validate chaperone binding sites identified by Hdocking for desmin fragment monomer, and to experimentally identify binding sites for desmin fragment seed, we performed cross-linking mass spectrometry (XL-MS) with MS-cleavable disuccinimidyl sulfoxide (DSSO) linker. This chemical cross-linker contains two NHS ester groups that bind to primary amine groups, linking lysine residues. The DSSO spacer arm measures 10.1 Å and can cross-link lysine residues whose C$\alpha$ atoms are 7–25 Å apart (58). While DSSO literature mainly focuses on cross-links between lysines (primary amines), DSSO can also cross-link hydroxyl side chains and the N-terminal amine (59). Using XL-MS, we determined all major cross-links between chaperone and monomer or chaperone and seed (Tables S1 and S2). We showed these cross-links graphically as lines (bottom panels) and also overlaid these lines in the Hdock model image (top panels, Figs. 4 *C* and *D*; S10 and S11). Observed cross-links indicated binding between the chaperone and monomer or chaperone and seed.

Desmin fragment has one lysine at K15 (full-length K125). Each $\alpha$B-crystallin has 10 lysines: one in the NTD, five in the ACD, and four in the CTD. XL-MS results indicated $\alpha$B-crystallin S59, K72, K150, and K175 were cross-linked to monomer K15/K125 (Table S1). We also observed $\alpha$B-crystallin K150 was cross-linked to the N-terminal amine of desmin fragment monomer. When we compared the XL-MS results to the Hdocking binding model, all the observed cross-linked $\alpha$B-crystallin lysines and serines were within 25 Å of monomeric desmin fragment K125 or N-terminal amine (Fig. 4 *C*). Thus, the HDOCK model for monomer+$\alpha$B-crystallin agreed with the XL-MS data results (Fig. S10). Note that XL-MS data did not identify chaperone oligomerization or secondary structure of desmin fragment; XL-MS only measured which cross-links were present. Along with Hdocking, we used CD, FTIR, and gel cross-linking to inform the structural cartoons that contain distances that agreed with XL-MS data (Figs. S1;3 *C*; S9).

For the seed, XL-MS results indicated $\alpha$B-crystallin K72, K90, K150, T158, K166, K174, and K175 were cross-linked to seed K15/K125 (Table S1). The N terminus of $\alpha$B-crystallin was cross-linked to both the N terminus and K15/K125 of desmin fragment seed. Compared with monomer, the interactions between seed and $\alpha$B-crystallin included overall more cross-link locations ($\alpha$B-crystallin K90, T158, K166, K174, N terminus), but we do not observe the monomer interaction at $\alpha$B-crystallin S59 for the seed. Literature suggests that amyloid structures bind at the chaperone's ACD dimer interface (16,29), and we observed two seed interactions at the ACD dimer interface. The majority of cross-links we observed between chaperone and seed were surprisingly at the N terminus or in the CTD, instead of in the ACD. The absence of more cross-links within the ACD core was potentially due to lysines oriented inward toward the central pore of large chaperone oligomers, potentially sterically inaccessible to DSSO if seed binds instead on the solvent-exposed surface. We did not observe any seed interactions with $\alpha$B-crystallin K103, but we did observe cross-links between K90 of one $\alpha$B-crystallin and K103 of another (Fig. S12).

We also performed XL-MS for monomer or seed and HSP27 interactions. HSP27 has 7 lysines per chain: none in the NTD, five in the ACD, and two in the CTD. XL-MS results indicated HSP27 K198 and K205 were cross-linked to monomer K15/K125 (Fig. 4 *D*; Table S2). Similar to $\alpha$B-crystallin, HSP27 XL-MS results agreed with the HDOCK model (<25 Å, Fig. S11). Unlike $\alpha$B-crystallin, we did not observe monomer cross-links with the ACD of HSP27, or monomer N terminus cross-links. For the seed, XL-MS indicated HSP27 K123, K198, and K205 were cross-linked to seed K15/K125, and HSP27 N terminus, K198, and K205 were cross-linked to seed N terminus. Compared with monomer, the interactions between seed and HSP27 included overall more cross-link locations, including interactions at one location in the ACD core.

We did not observe any seed interactions with K114, but we did observe cross-links between K144 of one HSP27 and the N terminus of another (Fig. S12). Overall, desmin had fewer cross-links with HSP27 as both a monomer or a seed than desmin with $\alpha$B-crystallin. Due to the fact that desmin fragment amyloid inhibition required more HSP27 than $\alpha$B-crystallin and that HSP27 resulted in larger fibril lengths and widths (Fig. 2), we learned HSP27 had decreased chaperone activity for desmin as compared with $\alpha$B-crystallin. The XL-MS data reporting fewer cross-links between desmin fragment and HSP27 indicated one reason for this decreased activity.

## Chaperones may interact with desmin fragment monomer reversibly and desmin fragment amyloid irreversibly

Because both tryptophan intrinsic fluorescence and XL-MS indicated that both chaperones had different interactions with monomer versus seed, we designed an assay to further probe these binding differences. We incubated chaperone with only monomer for 2 h and then added seed, or we incubated the chaperone with only seed for 2 h and then added monomer before performing ThT assays. When we incubated chaperones with seed first and then added desmin monomer, we did not observe any aggregation in the presence of either $\alpha$B-crystallin or HSP27 (Fig. 5 *A*). The TEM images showed only the initial seed and amorphous structures (Fig. S13). Because we did not observe amyloid fibril formation, we hypothesized that chaperones cap seed fibrils to prevent any seed templating monomeric desmin aggregation. This capping occurred at fibril ends (seeds do not grow longer) but also at fibril sides (seed width does not increase over time, Fig. 3 *B*; we observed chaperone along the fibril sides in TEM images). However, if the chaperones were only to interact with seed, then this remaining concentration of monomer (128 $\mu$M) would aggregate alone in solution. Thus, the seed-bound chaperone must have binding sites open for monomer or amorphous oligomer interactions, or enough chaperone remained unbound to seed and available to interact with monomer. We additionally confirmed that $\alpha$B-crystallin binds to both monomer and seed, through a BLI experiment (Fig. S14). Because the seed fibrils vary in size and the larger client contains the tag for the BLI sensor, we could not determine an accurate Kd, but we could interpret the results qualitatively to show that $\alpha$B-crystallin binds to both monomer and seed in a distinct manner.

In contrast, when we incubated chaperones with monomer first and then added seed, we observed aggregation. The $\alpha$B-crystallin+monomer, then seed, resulted in a T50 that was significantly slower than monomer+seed, but significantly faster than monomer alone (T50 $\alpha$B+monomer, then seed: 27.44 $\pm$ 1.38 h versus T50 monomer+seed: 15.34 $\pm$ 4.34 h, $p < 0.05$, or versus T50 monomer alone: 35.91 $\pm$ 2.49 h, $p < 0.05$, Figs. 3 *A* and 5 *A*). Thus, the $\alpha$B-crystallin retained some preventative chaperone activity against seed even when pre-incubated with monomer. In contrast, the HSP27+monomer, then seed resulted in a T50 that was statistically identical to monomer+seed (T50 HSP+monomer, then seed: 18.13 $\pm$ 1.49 h versus T50 monomer+seed: 15.34 $\pm$ 4.34 h, ttest2 not significantly different, Figs. 3 *A* and 5 *A*). The HSP27, when pre-incubated with monomer, did not retain any preventative chaperone activity against seed templating. However, we also knew that HSP27 could completely inhibit aggregation if seed and monomer were added to chaperone at the same time or if just monomer interacted with chaperone. Thus, along with concentration relationships, intrinsic fluorescence, HDOCK models, and XL-MS, we identified that HSP27 interacted differently with desmin fragment than $\alpha$B-crystallin, and it was a weaker chaperone for this client.

We performed DLS experiments on monomer alone, chaperone alone, or monomer+chaperone at 0 h, 2 h, and 19 h (Figs. 5 *B*; S15). Molecular weight estimates from the DLS data suggested that at 0 h, desmin fragment existed predominantly as a mixture of monomers and dimers. The sample also contained a minor population of larger oligomers (Fig. S15), consistent with our glutaraldehyde cross-linking experiments (Fig. S2). At 2 h, we only observed the monomer/dimer peak and not the large oligomeric peak. Taking this fact along with our TEM images of 640 $\mu$M desmin at 1.5 h showing no fibrils (Fig. S4), the desmin may have formed a low concentration of very long fibril species (not circular enough for DLS detection, below ThT detection limit), or the amorphous desmin oligomers may have disaggregated. Note that at 2 h, the monomer/dimer desmin peak was the predominant species present. At 19 h, we detected a broad distribution of hydrodynamic diameters for desmin, indicative of aggregation and corresponding to the ThT aggregation curve plateau (Fig. 1 *B*).

In contrast, DLS of chaperone+monomer at 0 h showed a bimodal distribution corresponding to both the desmin monomer/dimer peak and the chaperone oligomer peak (Figs. 5 *B*; S15). The hydrodynamic diameter of the both the $\alpha$B-crystallin and HSP27 peak increased in the presence of desmin at 0 h, indicating immediate binding. This immediate diameter increase was unexpected, as previous literature found no increase for $\alpha$B-crystallin plus monomeric $\alpha$-synuclein (31). Our cross-linking gel study suggested that the active interacting form of $\alpha$B-crystallin with desmin fragment was potentially $\geq$12-mer, although smaller active oligomer assemblies of chaperone could not be ruled out (Fig. S9). At 2 h, no desmin monomer/dimer peak remained in the presence of either chaperone, and the chaperone complex increased in size. This finding was unexpected, as previous DLS data from literature showed that monomeric A$\beta$ remained unbound to $\alpha$B-crystallin at a ratio of 1:50 A$\beta$ to chaperone after 4 days of aggregation (29). Our observed DLS diameter increase for chaperone+desmin fragment at 2 h suggested that all desmin interacted with chaperone at this point, either as amorphous

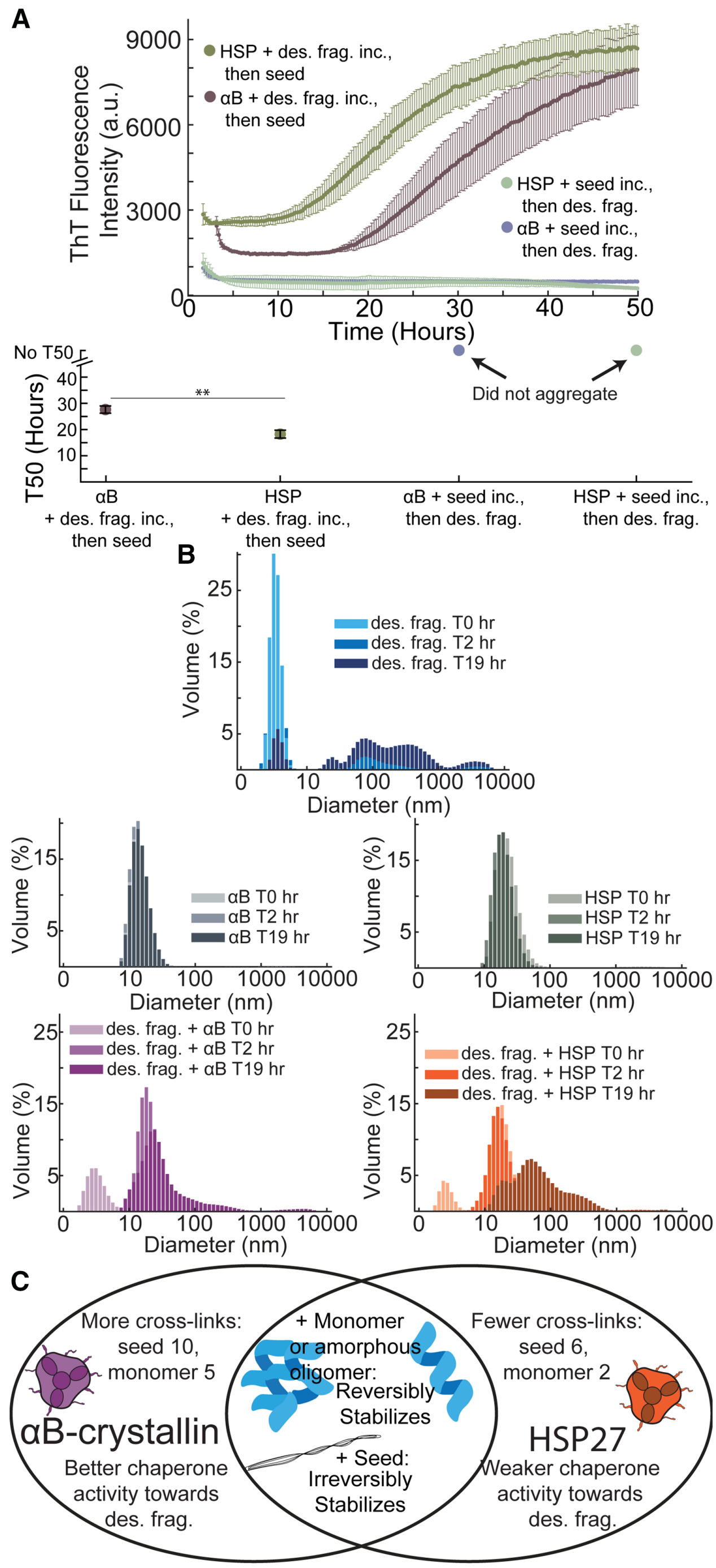


FIGURE 5 Chaperone proteins $\alpha$B-crystallin and HSP27 may interact reversibly with monomeric desmin fragment and irreversibly with desmin fragment amyloid. (*A*) ThT fluorescence of chaperone+seed incubated for 2 h prior to adding desmin fragment monomer compared with chaperone+monomer incubated for 2 h prior to adding seed for 128 $\mu$M monomer, 64 $\mu$M seed, 5.46 $\mu$M $\alpha$B-crystallin, and/or 4.83 $\mu$M HSP27. Values were averaged for T50s ($n = 3$) with 95% confidence interval shown. **: $p < 0.005$. (*B*) Averaged DLS intensity by volume plots of desmin fragment monomer (640 $\mu$M), chaperone alone (5.46 $\mu$M $\alpha$B-crystallin or 4.83 $\mu$M HSP27), or chaperone+monomer for 0 h, 2 h, and 19 h ($n = 6$). (*C*) A summary comparison of how chaperone proteins $\alpha$B-crystallin and HSP27 interacted with desmin fragment monomer or amorphous oligomer and amyloid seed.

oligomers or monomer, since both sizes were present for desmin alone in solution at 2 h (and desmin alone in solution at 2 h was predominantly monomer/dimer). Due to the high concentration of desmin compared with chaperone, the chaperones likely sequestered desmin as amorphous aggregates (32).

Our pre-incubation experiment indicated that the chaperone could potentially be saturated with monomer/amorphous oligomers that reversibly interact with chaperone. In the chaperone+monomer, then seed experiment, the chaperones had no sites left available to interact with late incoming seed, but they allowed monomer to be released and aggregate with seed in solution. The HSP27+monomer binding interaction appeared weaker than the $\alpha$B+monomer interaction because the HSP27+monomer, then seed had no T50 difference from monomer+seed alone, suggesting that monomer reversibly released from HSP27 faster. Our DLS data also supported this difference, since larger chaperone+desmin complexes were observed at 19 h for HSP27 compared with $\alpha$B-crystallin (Fig. 5 *B*). For the chaperone+seed, then monomer, the chaperones capped the ends and sides of the seed and still interacted with monomer. One explanation for this behavior was that only a small amount of chaperone was needed to fully interact with seed, leaving unbound chaperone available to interact with monomer. Another explanation was that chaperone interacting with seed contains other sites that could simultaneously interact with monomer, such as binding at S59 identified on $\alpha$B-crystallin by XL-MS that was only observed for monomer and not seed (Fig. 4 *C*). Our data suggested that the chaperone+seed interactions are irreversible, since we did not observe seed length or width changing overtime in the presence of chaperone (Fig. 3 *B*).

Overall, both chaperones acted protectively toward desmin fragment, preventing monomer and seed from additional amyloid fibril formation (Fig. 5 *C*). Our experiments identified better chaperone activity from $\alpha$B-crystallin toward desmin fragment, likely due to increased binding sites (cross-links) observed as compared with HSP27 (Fig. 5 *C*). The experiments in this work support that the inhibitory effect of these chaperones at substoichiometric quantities arises from a combination of factors: macromolecular crowding, sequestration of desmin fragment into insoluble amorphous aggregates, potentially irreversible capping of seed ends and sides, and potentially reversible, transient interactions between chaperones and desmin fragment oligomers that reduce the availability of aggregation-competent species.

## DISCUSSION

In this work, we demonstrated that cardiomyopathy-relevant desmin fragment can form amyloid fibrils under neutral pH, acidic conditions, or seeding conditions. Because both of the chaperones $\alpha$B-crystallin and HSP27 are overexpressed in heart disease, we examined how each of these chaperones interacted with desmin fragment. Unlike nonspecific control interactions (BSA), both chaperones actively prevented

amyloid formation. We found that even extremely small ratios of chaperone to monomeric client prevented amyloid formation, with kinetic delays observed as low as 1:12,800 for $\alpha$B-crystallin and 1:2,909 for HSP27. The physiologically relevant concentration of $\alpha$B-crystallin in cardiac tissue is 3 mg/mL and 0.5 mg/mL for HSP27, with overexpression above this in disease states (calculation in materials and methods) (20,22). Thus, in a healthy heart, both chaperones exist at a much greater concentration than those studied here (0.11 mg/mL [5.46 $\mu$M $\alpha$B or 4.83 $\mu$M HSP] and below). However, our experiments focus on a controlled system, as compared with the many different types of proteins present in the heart and the many different roles chaperones must play in tissue. Therefore, the low-concentration behavior we monitored applies to heart disease states when chaperones are already overwhelmed. Our work here showed that both $\alpha$B-crystallin and HSP27 can interact with desmin fragments and reduce overall amyloid formation, thus acting protectively to prevent desmin-related cardiomyopathy.

When examining seed induced amyloid fibril formation, we found that both $\alpha$B-crystallin and HSP27 prevented seed from templating monomeric desmin. The initially added seed structures continued to persist at 50 h, indicating both chaperones did not function as disaggregases in our experiments. The chaperones did prevent the seeds from increasing in length and width over time. Given that $\alpha$B-crystallin and HSP27 inhibited or delayed fibril formation but did not remodel existing fibrils, their activity was more consistent with a holdase function (60). Unlike what we expected from the literature (29,31), we observed an immediate hydrodynamic increase using DLS for chaperones in the presence of monomer. Over time, despite the prevention of amyloid formation, we observed the complete disappearance of the monomer peak. We learned from our XL-MS experiments that $\alpha$B-crystallin and HSP27 bind to both desmin fragment monomer and seed, with fewer binding sites for monomer than seed, and fewer binding sites for HSP27 than $\alpha$B-crystallin for both monomer and seed. Beyond expected interactions of seed with the ACD of each chaperone (16,29), we also observed interactions of seed with the N terminus and CTD of each chaperone.

Based on our pre-incubation ThT experiment, we found that chaperones appeared to interact irreversibly with mature amyloid seed and reversibly with monomeric desmin fragment. While chaperones completely prevented amyloid formation when both seed and monomer were simultaneously combined, the initial pre-incubation of chaperone with monomer resulted in complete amyloid formation after seed addition. This experiment provided supporting evidence for one of the ways amyloid diseases might propagate: chaperones overwhelmed by transient interactions with monomeric client cannot switch to seed/amyloid client to prevent templating. We also found supporting evidence through this experiment of HSP27's weaker chaperone activity: under these stressed conditions, while $\alpha$B-crystallin could delay amyloid formation, HSP27 did not impact aggregation T50 at all. A larger proportional concentration was needed for the weaker HSP27 chaperone activity compared with $\alpha$B-crystallin. In the heart, $\alpha$B-crystallin was shown to interact with many clients, and particularly with desmin, but HSP27, although still found at high heart concentrations and capable of interacting with desmin, was shown to interact predominantly with actin (49,50). Therefore, structural and sequence differences between desmin and actin may explain fewer binding sites observed for HSP27 and may explain why $\alpha$B-crystallin was a better chaperone for desmin fragment than HSP27 in these experiments.

Throughout this study, we gained insights into how two different chaperones prevent amyloid fibril formation. Amyloid fibrils as well as aggregation intermediates are generally understood to be toxic to cells. Thus with our work, we aim to aid the treatment of misfolding and aggregation diseases by improving our understanding of peptide-chaperone interactions. Our insights into peptide-chaperone interactions promotes the future employment of chaperones for future treatment or prevention of aggregation diseases. Pharmacological chaperones, or small molecules that mimic protein chaperone functions, can successfully treat protein misfolding diseases (35). Artificial chaperones constructed from designed materials can mimic the activity of natural chaperone proteins, offering promising therapeutic approaches to a variety of diseases (61,62). However, much must first be understood about individual client-chaperone contacts for further pharmacological chaperone development. Our work advances the understanding of how chaperone proteins modulate desmin fragment amyloid aggregation, paving the way for developing pharmacological or artificial chaperones as potential treatments for desmin-related cardiomyopathies and other diseases associated with chaperone protein dysfunction. Overall, our studies shed light on the ability of chaperone proteins, $\alpha$B-crystallin and HSP27, to adaptively engage with multiple conformations of the same client protein, mediating potentially reversible interactions with monomer or amorphous aggregates through fewer binding sites and irreversible amyloid binding through additional binding sites to prevent further amyloid aggregation.

## DATA AND CODE AVAILABILITY

The associated research dataset has been deposited to UD Dataverse, the University of Delaware Institutional Data Repository, under the title "Chaperone proteins protect against desmin fragment amyloid aggregation" and is publicly available as of the article publication date.

## ACKNOWLEDGMENTS

E.M.M. was supported by the University of Delaware Chemistry Biology Interface (T32-GM133395) and then the National Science Foundation Graduate Research Fellowship under grant no. 1940700.

This work was supported by NIH COBRE Pilot Project grants (2P20GM113125 and P30GM159572) and then by a NIH COBRE Research Project grant (P20GM156674).

Access to the TEM was supported by the NIH (P20GM103446). Microscopy access was supported by grants from the NIH-NIGMS (P20GM103446), the NIGMS (P20GM139760), and the State of Delaware. Microscopy equipment at the University of Delaware was acquired with support of the Unidel Foundation.

We acknowledge support from the National Institute of General Medical Sciences under award number P20GM104316 for the mass spectrometry instruments, and Dr. Yanbao Yu for assistance with MS analysis.

We thank Youmna Moawad and Dr. Jeffrey Mugridge for their invaluable assistance with optimization of recombinant protein expression and purification.

The content is solely the responsibility of the authors and does not necessarily represent the official views of the National Institutes of Health or of the National Science Foundation.

## AUTHOR CONTRIBUTIONS

A.M.A. and E.M.M. designed and performed research, E.M.M. analyzed the data, and A.M.A. and E.M.M. wrote the paper.

## DECLARATION OF INTERESTS

The authors declare no competing interests.

## SUPPORTING MATERIAL

Supporting material can be found online at https://doi.org/10.1016/j.bpj.2026.03.038.

## REFERENCES

1. Martin, S. S., A. W. Aday, …, American Heart Association Council on Epidemiology and Prevention Statistics Committee and Stroke Statistics Subcommittee. 2024. 2024 Heart Disease and Stroke Statistics: A Report of US and Global Data From the American Heart Association. *Circulation.* 149:e347–e913. https://doi.org/10.1161/CIR.0000000000001209.

2. Agnetti, G., V. L. Halperin, …, J. E. Van Eyk. 2014. Desmin modifications associate with amyloid-like oligomers deposition in heart failure. *Cardiovasc. Res.* 102:24–34. https://doi.org/10.1093/cvr/cvu003.

3. McLendon, P. M., and J. Robbins. 2011. Desmin-related cardiomyopathy: an unfolding story. *Am J Physiol-Heart C.* 301:H1220–H1228. https://doi.org/10.1152/ajpheart.00601.2011.

4. Sanbe, A., H. Osinska, …, J. Robbins. 2004. Desmin-related cardiomyopathy in transgenic mice: A cardiac amyloidosis. *Proc. Natl. Acad. Sci. USA.* 101:10132–10136. https://doi.org/10.1073/pnas.0401900101.

5. Bär, H., N. Mücke, …, H. Herrmann. 2005. Severe muscle disease-causing desmin mutations interfere with *in vitro* filament assembly at distinct stages. *Proc. Natl. Acad. Sci. USA.* 102:15099–15104. https://doi.org/10.1073/pnas.0504568102.

6. Herrmann, H., E. Cabet, …, R. Schröder. 2020. Dual Functional States of R406W-Desmin Assembly Complexes Cause Cardiomyopathy With Severe Intercalated Disc Derangement in Humans and in Knock-In Mice. *Circulation.* 142:2155–2171. https://doi.org/10.1161/CIRCULATIONAHA.120.050218.

7. Agnetti, G., H. Herrmann, and S. Cohen. 2022. New roles for desmin in the maintenance of muscle homeostasis. *FEBS J.* 289:2755–2770. https://doi.org/10.1111/febs.15864.

8. Even, C., G. Abramovici, …, F. Briki. 2017. Mutation in the Core Structure of Desmin Intermediate Filaments Affects Myoblast Elasticity. *Biophys. J.* 113:627–636. https://doi.org/10.1016/j.bpj.2017.06.020.

9. Rainer, P. P., P. Dong, …, G. Agnetti. 2018. Desmin Phosphorylation Triggers Preamyloid Oligomers Formation and Myocyte Dysfunction in Acquired Heart Failure. *Circ. Res.* 122:e75–e83. https://doi.org/10.1161/CIRCRESAHA.117.312082.

10. Chen, F., R. Chang, …, V. L. Cryns. 2003. Caspase Proteolysis of Desmin Produces a Dominant-negative Inhibitor of Intermediate Filaments and Promotes Apoptosis. *J. Biol. Chem.* 278:6848–6853. https://doi.org/10.1074/jbc.M212021200.

11. Moneo-Corcuera, D., Á. Viedma-Poyatos, …, D. Pérez-Sala. 2023. Desmin Reorganization by Stimuli Inducing Oxidative Stress and Electrophiles: Role of Its Single Cysteine Residue. *Antioxidants.* 12:1703. https://doi.org/10.3390/antiox12091703.

12. Kedia, N., K. Arhzaouy, …, J. Bieschke. 2019. Desmin forms toxic, seeding-competent amyloid aggregates that persist in muscle fibers. *Proc. Natl. Acad. Sci. USA.* 116:16835–16840. https://doi.org/10.1073/pnas.1908263116.

13. Sunde, M., L. C. Serpell, …, C. C. F. Blake. 1997. Common Core Structure of Amyloid Fibrils by Synchrotron X-ray Diffraction. *J. Mol. Biol.* 273:729–739. https://doi.org/10.1006/jmbi.1997.1348.

14. Dobson, C. M. 2017. The Amyloid Phenomenon and Its Links with Human Disease. *CSH Perspect Biol.* 9:a023648. https://doi.org/10.1101/cshperspect.a023648.

15. Knauer, M. F., B. Soreghan, …, C. G. Glabe. 1992. Intracellular accumulation and resistance to degradation of the Alzheimer amyloid A4/beta protein. *Proc. Natl. Acad. Sci. USA.* 89:7437–7441. https://doi.org/10.1073/pnas.89.16.7437.

16. Hochberg, G. K. A., H. Ecroyd, …, A. Laganowsky. 2014. The structured core domain of αB-crystallin can prevent amyloid fibrillation and associated toxicity. *Proc. Natl. Acad. Sci. USA.* 111:E1562–E1570. https://doi.org/10.1073/pnas.1322673111.

17. Stepanenko, O. V., M. I. Sulatsky, …, A. I. Sulatskaya. 2020. Alpha-B-Crystallin Effect on Mature Amyloid Fibrils: Different Degradation Mechanisms and Changes in Cytotoxicity. *Int. J. Mol. Sci.* 21:7659. https://doi.org/10.3390/ijms21207659.

18. Selig, E. E., C. O. Zlatic, …, M. D. Griffin. 2020. N- and C-terminal regions of αB-crystallin and Hsp27 mediate inhibition of amyloid nucleation, fibril binding, and fibril disaggregation. *J. Biol. Chem.* 295:9838–9854. https://doi.org/10.1074/jbc.RA120.012748.

19. Horwitz, J. 2000. The function of alpha-crystallin in vision. *Semin. Cell Dev. Biol.* 11:53–60. https://doi.org/10.1006/scdb.1999.0351.

20. Wang, X., R. Klevitsky, …, J. Robbins. 2003. αB-Crystallin Modulates Protein Aggregation of Abnormal Desmin. *Circ. Res.* 93:998–1005. https://doi.org/10.1161/01.RES.0000102401.77712.

21. Ito, H., K. Kamei, …, K. Kato. 2003. Hsp27 suppresses the formation of inclusion bodies induced by expression of R120GαB-crystallin, a cause of desmin-related myopathy. *Cell. Mol. Life Sci.* 60:1217–1223. https://doi.org/10.1007/s00018-003-3024-9.

22. Inaguma, Y., K. Hasegawa, …, K. Kato. 1995. Induction of the Synthesis of hsp27 and αB Crystallin in Tissues of Heat-Stressed Rats and Its Suppression by Ethanol or an α1-Adrenergic Antagonist1. *J. Biochem.* 117:1238–1243. https://doi.org/10.1093/oxfordjournals.jbchem.a124850.

23. Bova, M. P., O. Yaron, …, J. Horwitz. 1999. Mutation R120G in αB-crystallin, which is linked to a desmin-related myopathy, results in an irregular structure and defective chaperone-like function. *Proc. Natl. Acad. Sci. USA.* 96:6137–6142. https://doi.org/10.1073/pnas.96.11.6137.

24. Wang, X., H. Osinska, …, J. Robbins. 2001. Expression of R120G–αB-Crystallin Causes Aberrant Desmin and αB-Crystallin Aggregation and Cardiomyopathy in Mice. *Circ. Res.* 89:84–91. https://doi.org/10.1161/hh1301.092688.

25. Alperstein, A. M., K. S. Molnar, …, U. P. Andley. 2021. Analysis of amyloid-like secondary structure in the Cryab-R120G knock-in mouse model of hereditary cataracts by two-dimensional infrared spectroscopy. *PLoS One.* 16:e0257098. https://doi.org/10.1371/journal.pone.0257098.

26. Elliott, J. L., M. Der Perng, …, R. A. Quinlan. 2013. The specificity of the interaction between α B-crystallin and desmin filaments and its impact on filament aggregation and cell viability. *Philos T Roy Soc B.* 368:20120375. https://doi.org/10.1098/rstb.2012.0375.

27. Sharma, S., G. M. Conover, …, R. A. Quinlan. 2017. αB-crystallin is a sensor for assembly intermediates and for the subunit topology of desmin intermediate filaments. *Cell Stress Chaperon.* 22:613–626. https://doi.org/10.1007/s12192-017-0788-7.

28. Peters, C., M. Haslbeck, and J. Buchner. 2024. Catchers of folding gone awry: a tale of small heat shock proteins. *Trends Biochem. Sci.* 49:1063–1078. https://doi.org/10.1016/j.tibs.2024.08.003.

29. Mainz, A., J. Peschek, …, B. Reif. 2015. The chaperone αB-crystallin uses different interfaces to capture an amorphous and an amyloid client. *Nat. Struct. Mol. Biol.* 22:898–905. https://doi.org/10.1038/nsmb.3108.

30. Baughman, H. E. R., T.-H. T. Pham, …, R. E. Klevit. 2020. Release of a disordered domain enhances HspB1 chaperone activity toward tau. *Proc. Natl. Acad. Sci. USA.* 117:2923–2929. https://doi.org/10.1073/pnas.1915099117.

31. Scheidt, T., J. A. Carozza, …, T. P. J. Knowles. 2021. The binding of the small heat-shock protein αB-crystallin to fibrils of α-synuclein is driven by entropic forces. *Proc. Natl. Acad. Sci. USA.* 118:e2108790118. https://doi.org/10.1073/pnas.2108790118.

32. Pippal, B., P. Chaudhuri, …, N. Jain. 2023. Discerning Modulation of α-Synuclein Amyloid Assembly by α-Crystallin. *ACS Chem. Neurosci.* 14:1659–1671. https://doi.org/10.1021/acschemneuro.3c00052.

33. Woods, C. N., M. K. Janowska, …, R. E. Klevit. 2025. Activation mechanism of small heat shock protein HSPB5 revealed by disease-associated mutants. *Proc. Natl. Acad. Sci. USA.* 122:e2425061122. https://doi.org/10.1073/pnas.2425061122.

34. Cox, D., and H. Ecroyd. 2017. The small heat shock proteins αB-crystallin (HSPB5) and Hsp27 (HSPB1) inhibit the intracellular aggregation of α-synuclein. *Cell Stress Chaperon.* 22:589–600. https://doi.org/10.1007/s12192-017-0785-x.

35. Pampalone, G., S. Grottelli, …, B. Cellini. 2021. Role of misfolding in rare enzymatic deficits and use of pharmacological chaperones as therapeutic approach. *Front. Biosci.* 26:1627–1642. https://doi.org/10.52586/5056.

36. Longoni, S., S. Lattonen, …, M. Chiesi. 1990. Cardiac alpha-crystallin. *Mol. Cell. Biochem.* 97:113–120. https://doi.org/10.1007/BF00221052.

37. Latchman, D. S. 2001. Heat shock proteins and cardiac protection. *Cardiovasc. Res.* 51:637–646. https://doi.org/10.1016/S0008-6363(01)00354-6.

38. Lutsch, G., R. Vetter, …, R. Benndorf. 1997. Abundance and Location of the Small Heat Shock Proteins HSP25 and αB-Crystallin in Rat and Human Heart. *Circulation.* 96:3466–3476. https://doi.org/10.1161/01.CIR.96.10.3466.

39. Gheorghe, A. G., A. Fuchs, …, J. Banner. 2019. Cardiac left ventricular myocardial tissue density, evaluated by computed tomography and autopsy. *BMC Med. Imaging.* 19:29. https://doi.org/10.1186/s12880-019-0326-4.

40. Xiong, Y. 2004. Muscle proteins. *In* Proteins in Food Processing. R. Yada, ed Woodhead Publishing, Cambridge, UK, pp. 100–122. https://doi.org/10.1533/9781855738379.1.100.

41. Schroeder, M. A., P. Swietach, …, D. J. Tyler. 2010. Measuring intracellular pH in the heart using hyperpolarized carbon dioxide and bicarbonate: a $^{13}C$ and $^{13}P$ magnetic resonance spectroscopy study. *Cardiovasc. Res.* 86:82–91. https://doi.org/10.1093/cvr/cvp396.

42. May, P. C., B. D. Gitter, …, P. M. Robison. 1992. β-amyloid peptide in vitro toxicity: Lot-to-lot variability. *Neurobiol. Aging.* 13:605–607. https://doi.org/10.1016/0197-4580(92)90064-5.

43. Barth, A. 2007. Infrared spectroscopy of proteins. *BBA - Bioenergetics.* 1767:1073–1101. https://doi.org/10.1016/j.bbabio.2007.06.004.

44. Shammas, S. L., C. A. Waudby, …, S. Meehan. 2011. Binding of the Molecular Chaperone αB-Crystallin to Aβ Amyloid Fibrils Inhibits Fibril Elongation. *Biophys. J.* 101:1681–1689. https://doi.org/10.1016/j.bpj.2011.07.056.

45. Ecroyd, H., and J. A. Carver. 2009. Crystallin proteins and amyloid fibrils. *Cell. Mol. Life Sci.* 66:62–81. https://doi.org/10.1007/s00018-008-8327-4.

46. Waudby, C. A., T. P. J. Knowles, …, S. Meehan. 2010. The Interaction of αB-Crystallin with Mature α-Synuclein Amyloid Fibrils Inhibits Their Elongation. *Biophys. J.* 98:843–851. https://doi.org/10.1016/j.bpj.2009.10.056.

47. Houck, S. A., A. Landsbury, …, R. A. Quinlan. 2011. Multiple Sites in αB-Crystallin Modulate Its Interactions with Desmin Filaments Assembled In Vitro. *PLoS One.* 6:e25859. https://doi.org/10.1371/journal.pone.0025859.

48. Selig, E. E., R. J. Lynn, …, M. D. Griffin. 2022. The Monomeric α-Crystallin Domain of the Small Heat-shock Proteins αB-crystallin and Hsp27 Binds Amyloid Fibril Ends. *J Mol Bio.* 434:167711. https://doi.org/10.1016/j.jmb.2022.167711.

49. Doshi, B. M., L. E. Hightower, and J. Lee. 2009. The role of Hsp27 and actin in the regulation of movement in human cancer cells responding to heat shock. *Cell Stress Chaperon.* 14:445–457. https://doi.org/10.1007/s12192-008-0098-1.

50. Paulsen, G., F. Lauritzen, …, T. Raastad. 2009. Subcellular movement and expression of HSP27, αB -crystallin, and HSP70 after two bouts of eccentric exercise in humans. *J. Appl. Physiol.* 107:570–582. https://doi.org/10.1152/japplphysiol.00209.2009.

51. Hu, X., S. L. Crick, …, J.-M. Lee. 2009. Amyloid seeds formed by cellular uptake, concentration, and aggregation of the amyloid-beta peptide. *Proc. Natl. Acad. Sci. USA.* 106:20324–20329. https://doi.org/10.1073/pnas.0911281106.

52. Peschek, J., N. Braun, …, J. Buchner. 2013. Regulated structural transitions unleash the chaperone activity of αB-crystallin. *Proc. Natl. Acad. Sci. USA.* 110:E3780–E3789. https://doi.org/10.1073/pnas.1308898110.

53. Duy, C., and J. Fitter. 2006. How Aggregation and Conformational Scrambling of Unfolded States Govern Fluorescence Emission Spectra. *Biophys. J.* 90:3704–3711. https://doi.org/10.1529/biophysj.105.078980.

54. Sha, E., M. Nakamura, …, M. Yohda. 2019. Functional and structural characterization of HspB1/Hsp27 from Chinese hamster ovary cells. *FEBS Open Bio.* 9:1826–1834. https://doi.org/10.1002/2211-5463.12726.

55. Lelj-Garolla, B., and A. G. Mauk. 2006. Self-association and Chaperone Activity of Hsp27 Are Thermally Activated. *J. Biol. Chem.* 281:8169–8174. https://doi.org/10.1074/jbc.M512553200.

56. Yammine, A., J. Gao, and A. H. Kwan. 2019. Tryptophan Fluorescence Quenching Assays for Measuring Protein-ligand Binding Affinities: Principles and a Practical Guide. *Bio. Protoc.* 9:e3253. https://doi.org/10.21769/BioProtoc.3253.

57. Yan, Y., H. Tao, …, S.-Y. Huang. 2020. The HDOCK server for integrated protein–protein docking. *Nat. Protoc.* 15:1829–1852. https://doi.org/10.1038/s41596-020-0312-x.

58. Liu, F., P. Lössl, …, A. J. R. Heck. 2017. Optimized fragmentation schemes and data analysis strategies for proteome-wide cross-link identification. *Nat. Commun.* 8:15473. https://doi.org/10.1038/ncomms15473.

59. Bartolec, T. K., X. Vázquez-Campos, …, J. K. K. Low. 2023. Cross-linking mass spectrometry discovers, evaluates, and corroborates structures and protein–protein interactions in the human cell. *Proc. Natl. Acad. Sci. USA.* 120:e2219418120. https://doi.org/10.1073/pnas.2219418120.

60. Rutledge, B. S., W.-Y. Choy, and M. L. Duennwald. 2022. Folding or holding?-Hsp70 and Hsp90 chaperoning of misfolded proteins in neurodegenerative disease. *J. Biol. Chem.* 298:101905. https://doi.org/10.1016/j.jbc.2022.101905.

61. March, Z. M., K. L. Mack, and J. Shorter. 2019. AAA+ Protein-Based Technologies to Counter Neurodegenerative Disease. *Biophys. J.* 116:1380–1385. https://doi.org/10.1016/j.bpj.2019.03.007.

62. Li, Y., D. Yin, …, Y. Lv. 2024. Engineered polymer nanoparticles as artificial chaperones facilitating the selective refolding of denatured enzymes. *Proc. Natl. Acad. Sci. USA.* 121:e2403049121. https://doi.org/10.1073/pnas.2403049121.

**Biophysical Journal, Volume *125***

## Supplemental information

# Chaperone proteins protect against desmin fragment amyloid aggregation

**Erin M. Mulhearn and Ariel M. Alperstein**

## Supplemental Materials and Methods for

# Chaperone proteins protect against desmin fragment amyloid aggregation

Erin M. Mulhearn[1] and Ariel M. Alperstein[1,*]

[1]Department of Chemistry and Biochemistry, University of Delaware, Newark Delaware
*Correspondence: amalpers@udel.edu

This Supplemental PDF file includes:

**Figures**

S1: Desmin fragment forms amyloid under acidic conditions
S2: SDS PAGE gel after crosslinking indicating purchased desmin fragment starts predominantly in monomeric form
S3: ThT fluorescence limit of detection for desmin fragment amyloid
S4: TEM data for all samples in main text Figure 1
S5: Additional TEM data supporting main text Figure 2E
S6: Additional TEM data supporting main text Figure 2F
S7: Additional TEM data supporting main text Figure 3B
S8: Additional TEM controls supporting main text Figure 3
S9: SDS PAGE gel after crosslinking potentially indicating active chaperones are in ≥12mer form
S10: Top scoring HDOCK models of desmin fragment + $\alpha$B-crystallin with overlaid XL-MS observed cross-links
S11: Top scoring HDOCK models of desmin fragment + HSP27 with overlaid XL-MS observed cross-links
S12: XL-MS intra-links of chaperone proteins $\alpha$B-crystallin and HSP27
S13: TEM data supporting main text Figure 5
S14: Bio-layer interferometry showing that chaperone $\alpha$B-crystallin is capable of binding to both (A) desmin monomer and (B) desmin seed
S15: DLS data supporting main text Figure 5

**Tables**

S1: XL-MS observed cross-links with desmin fragment and $\alpha$B-crystallin
S2: XL-MS observed cross-links with desmin fragment and HSP27

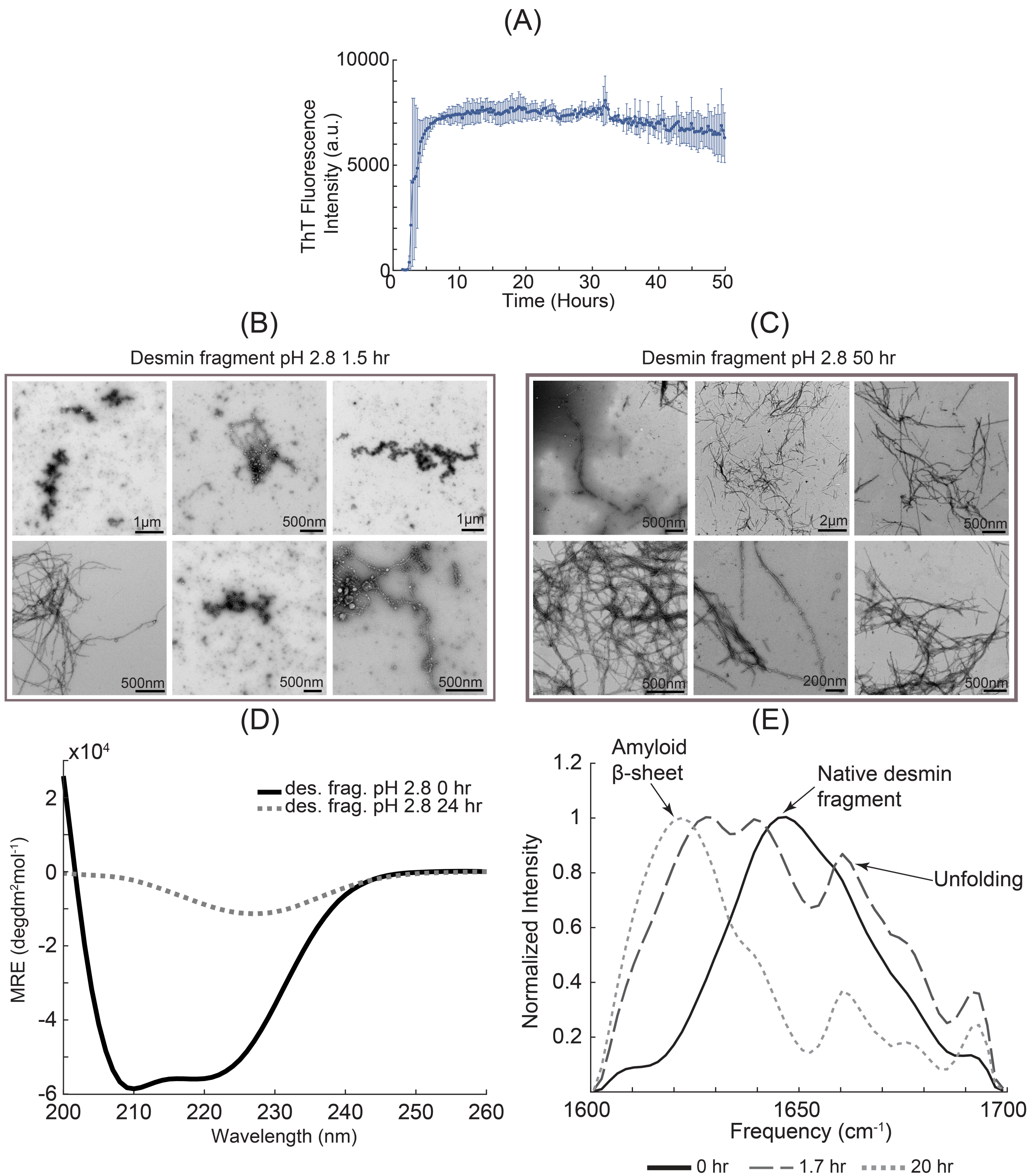


**Figure S1: Desmin fragment forms amyloid under acidic conditions.** (A) Desmin 256 $\mu$M at pH 2.8 amyloid kinetics as monitored by ThT fluorescence intensity. (B) TEM images were taken after 1.5 hour and (C) 50 hours into the aggregation process. (D) Circular dichroism spectra at time 0 (black) suggests desmin fragment after preparation has predominantly $\alpha$-helical structure. After 24 hours into the aggregation process, the spectrum shows $\beta$-sheet structure, indicating aggregation. (E) FTIR spectra of desmin fragment pH 2.8. Color gradient (light to dark) shows amide I peak shift over time, forming amyloid.

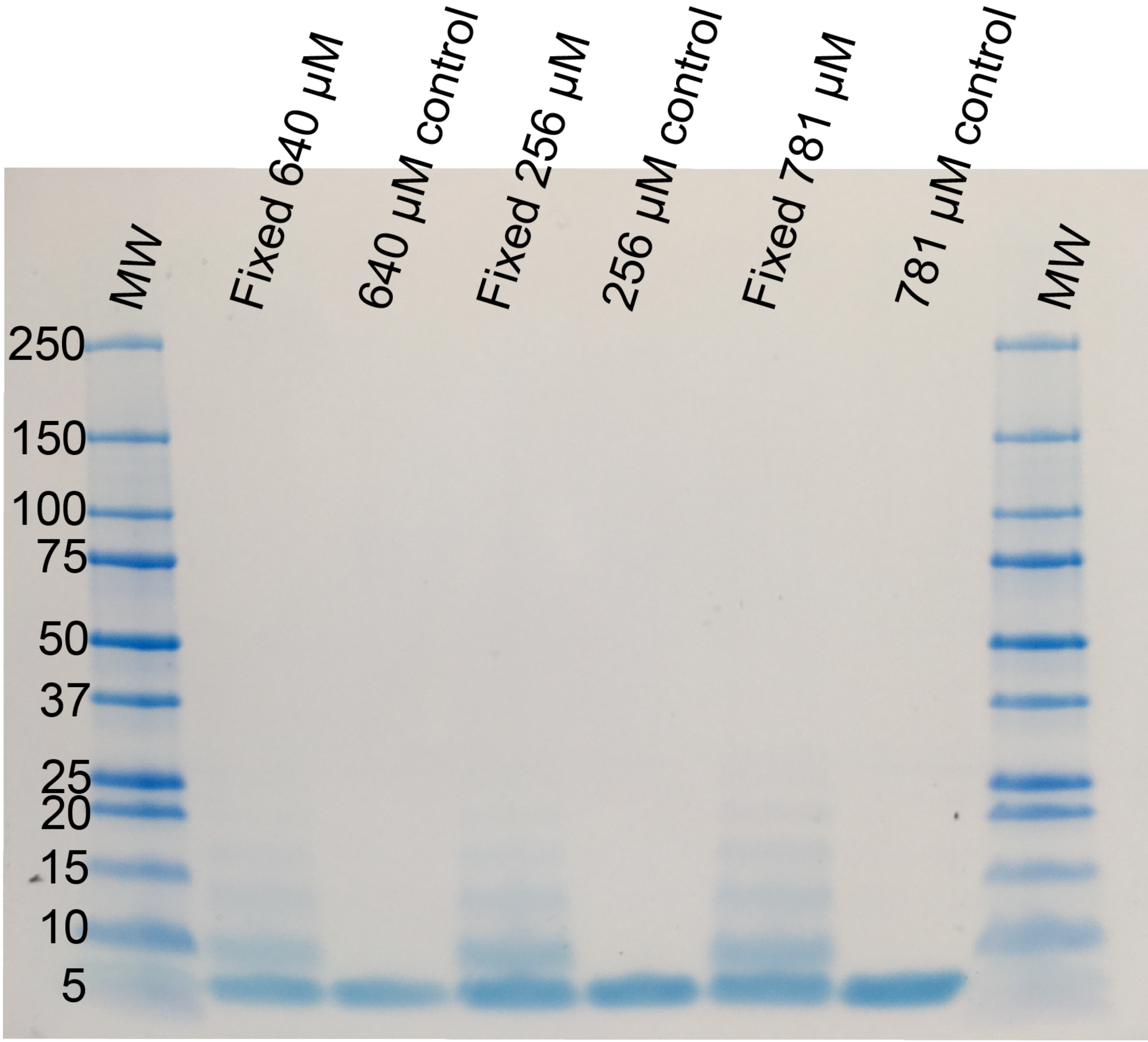


**Figure S2: SDS PAGE gel after crosslinking indicating purchased desmin fragment starts predominantly in monomeric form.** All fixed samples showed three faint higher molecular weight bands that represent transient oligomeric species but the majority of the samples exist as monomers. ImageJ calculations indicate at least 77% of desmin fragment is a monomer.

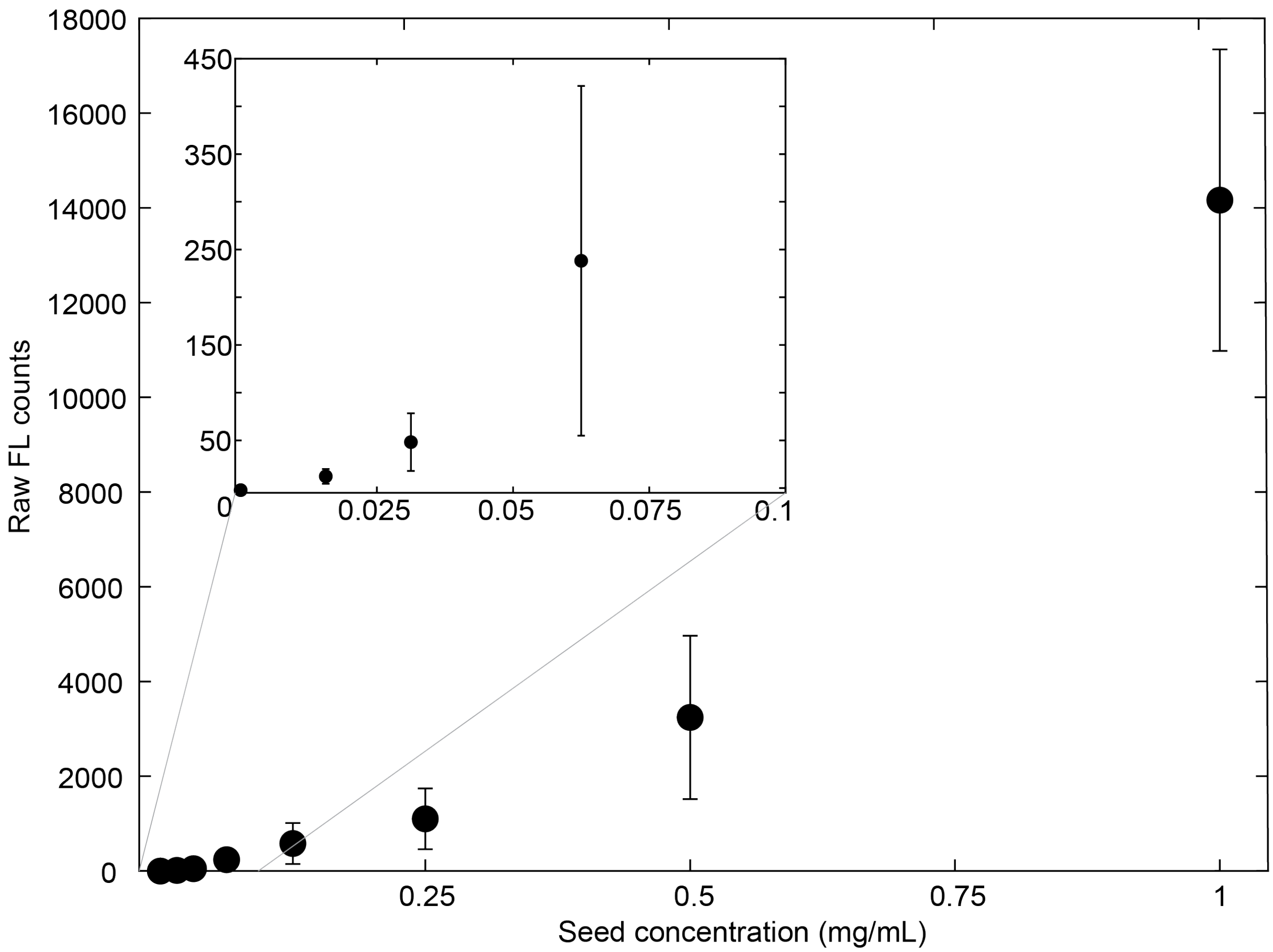


**Figure S3: ThT fluorescence limit of detection for desmin fragment amyloid.** ThT fluorescence intensity of desmin fragment seed at decreasing concentrations from 1 mg/mL (256 $\mu$M) to 0 mg/mL. The limit of detection for amyloid formation in our ThT assay is 0.06 mg/mL (15 $\mu$M). Triplicate raw data was averaged (N=3) and error bars are standard deviation.

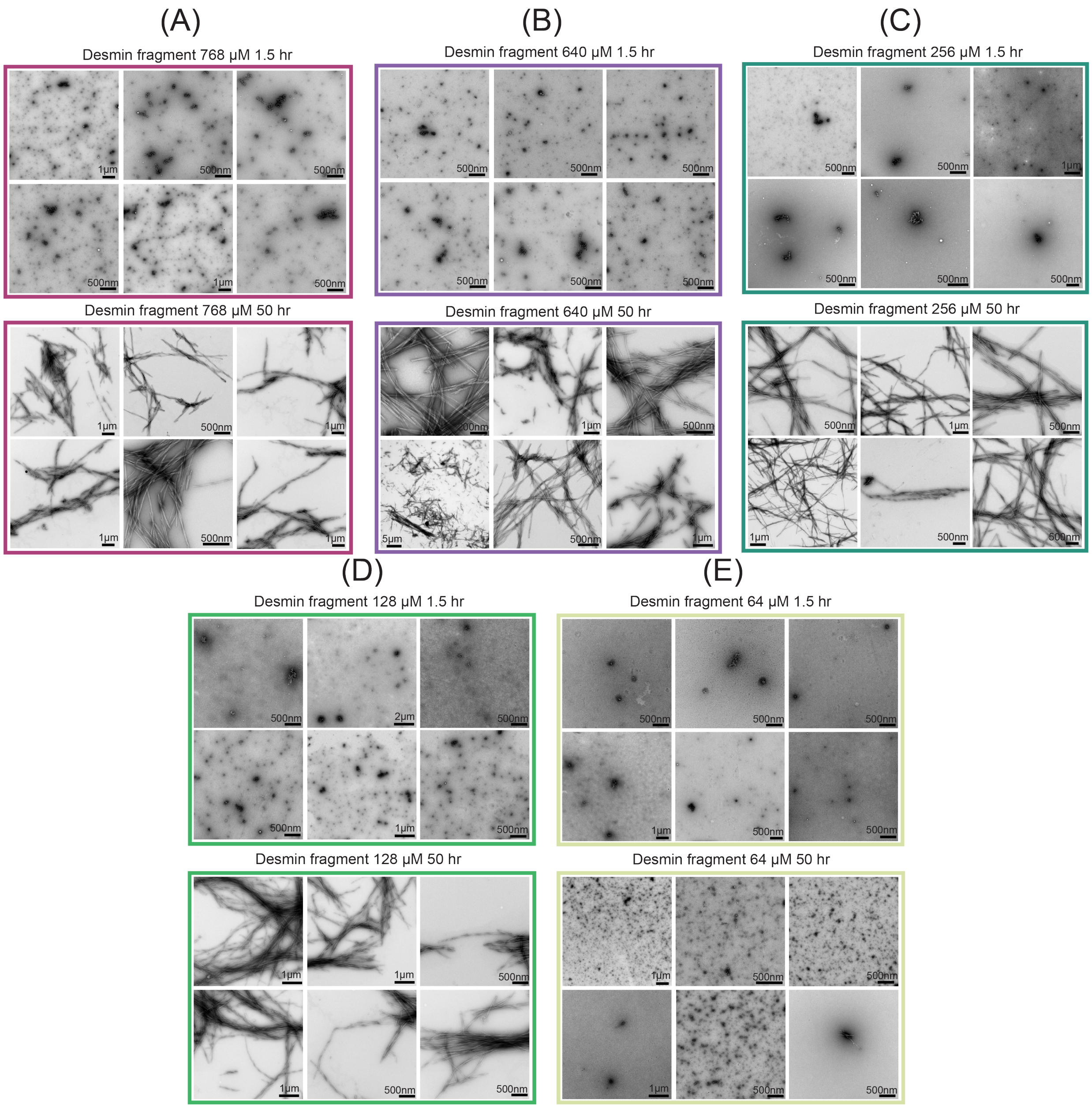


**Figure S4: TEM data for all samples in main text Figure 1.** (A-E) TEM images of desmin fragment at decreasing concentrations collected at time points 1.5 hours and 50 hours.

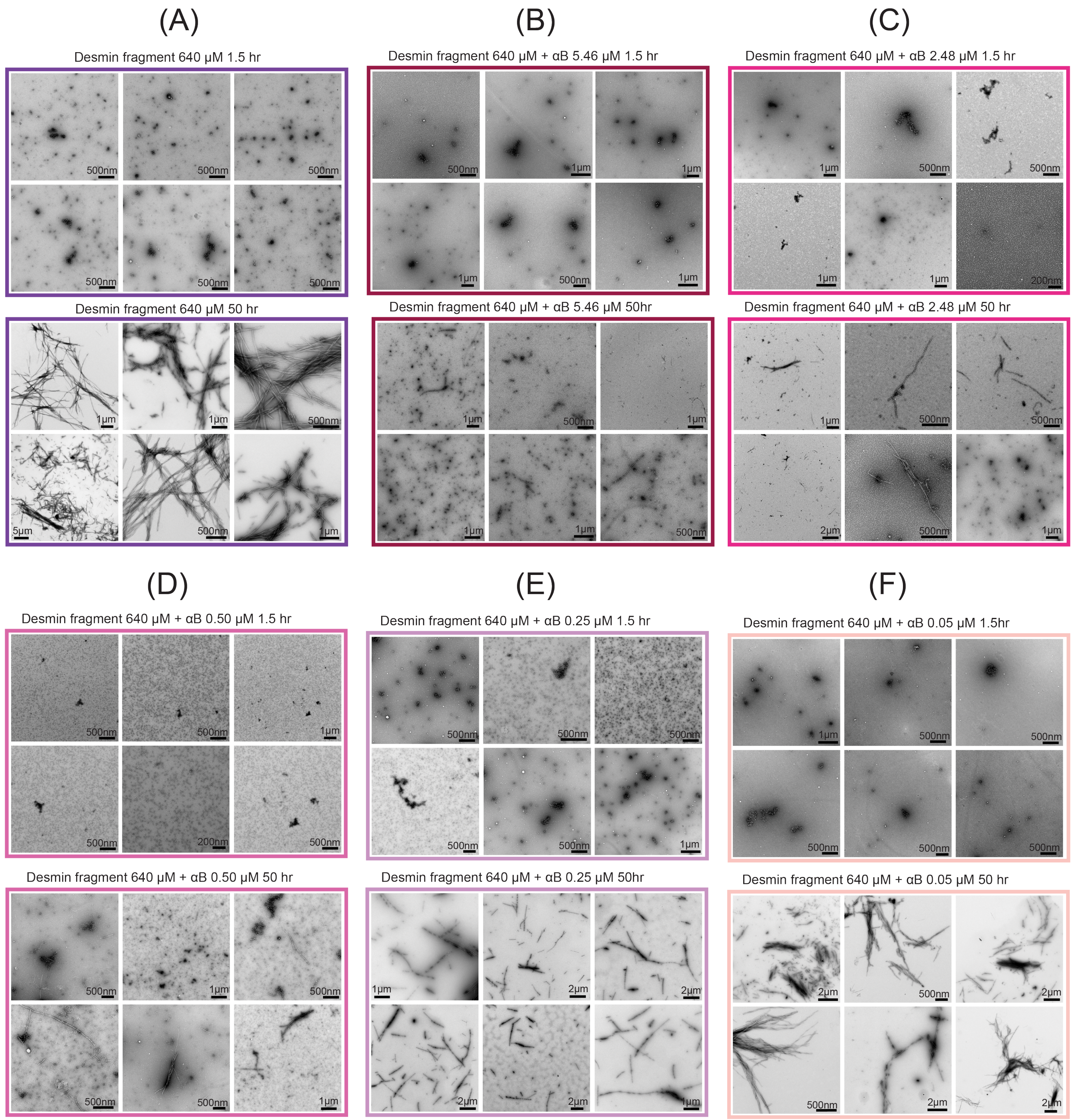


**Figure S5: Additional TEM data supporting main text Figure 2E.** (A-F) TEM images of desmin fragment at 640 $\mu$M and all concentrations of chaperone $\alpha$B-crystallin (0.05 to 5.46 $\mu$M) at time points 1.5 hours and 50 hours.

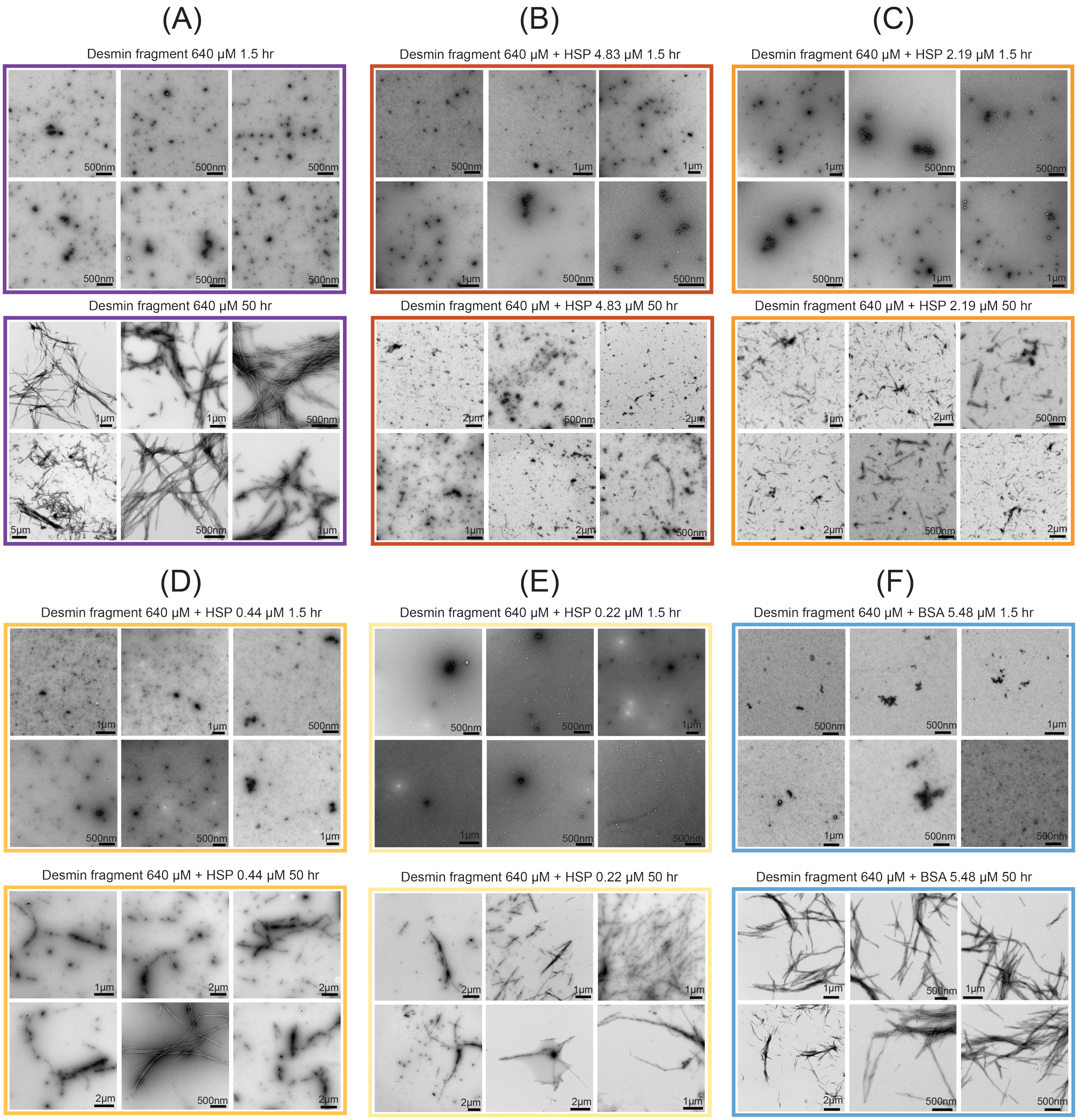


**Figure S6: Additional TEM data supporting main text Figure 2F.** (A-E) TEM images of desmin fragment at 640 $\mu$M and all concentrations of HSP27 (0.22 to 4.83 $\mu$M) at time points 1.5 hours and 50 hours. (F) Control sample of desmin with crowder BSA is also shown at time points 1.5 hours and 50 hours.

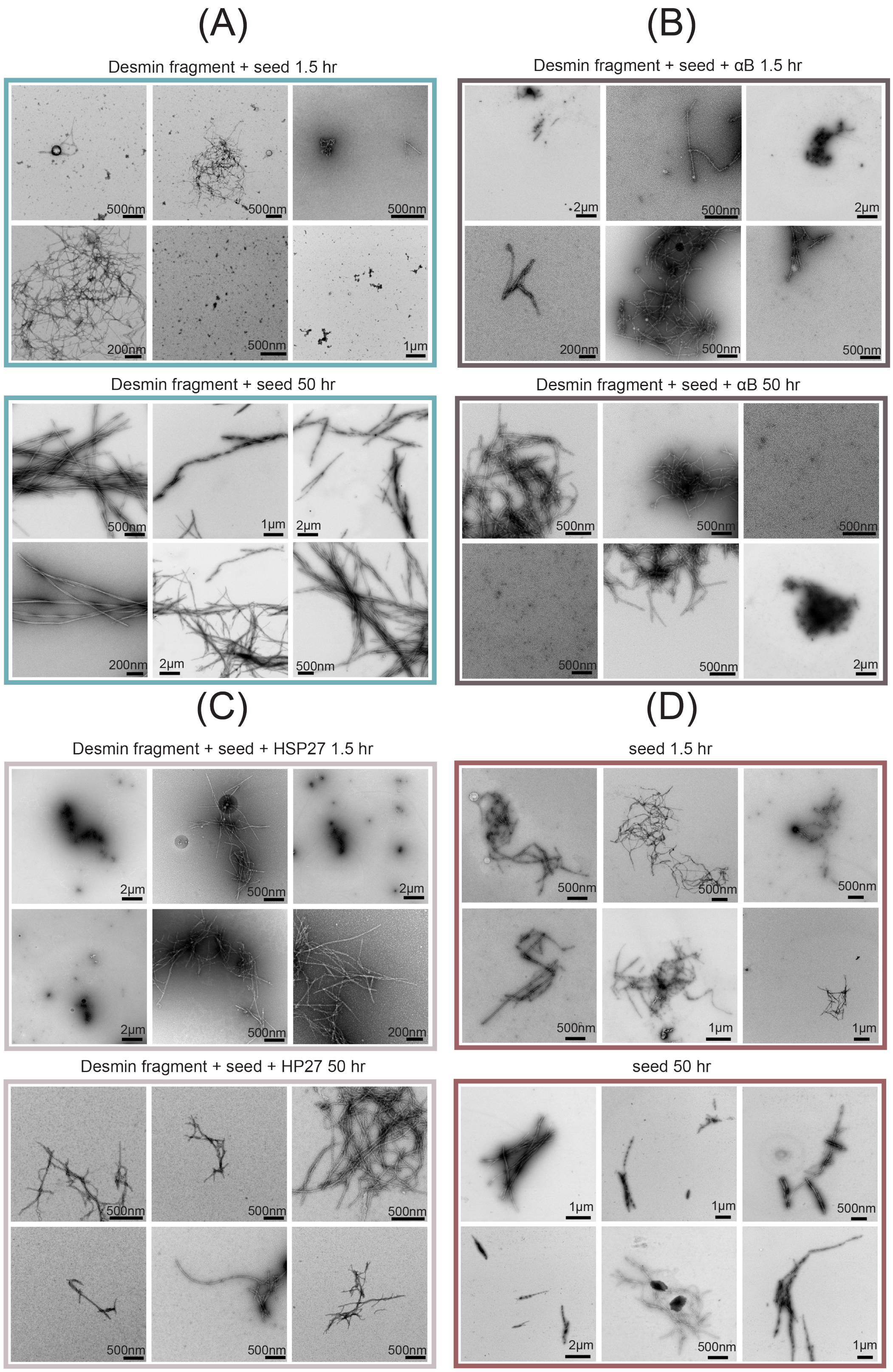


**Figure S7: Additional TEM data supporting main text Figure 3B.** (A-D) TEM images of desmin fragment + seed, desmin fragment + seed + $\alpha$B, desmin fragment + seed + HSP27 and seed alone (control) at time points 1.5 hours and 50 hours. Concentrations were 128 $\mu$M desmin fragment monomer, 64 $\mu$M seed, 5.46 $\mu$M $\alpha$B-crystallin, and 4.83 $\mu$M HSP27.

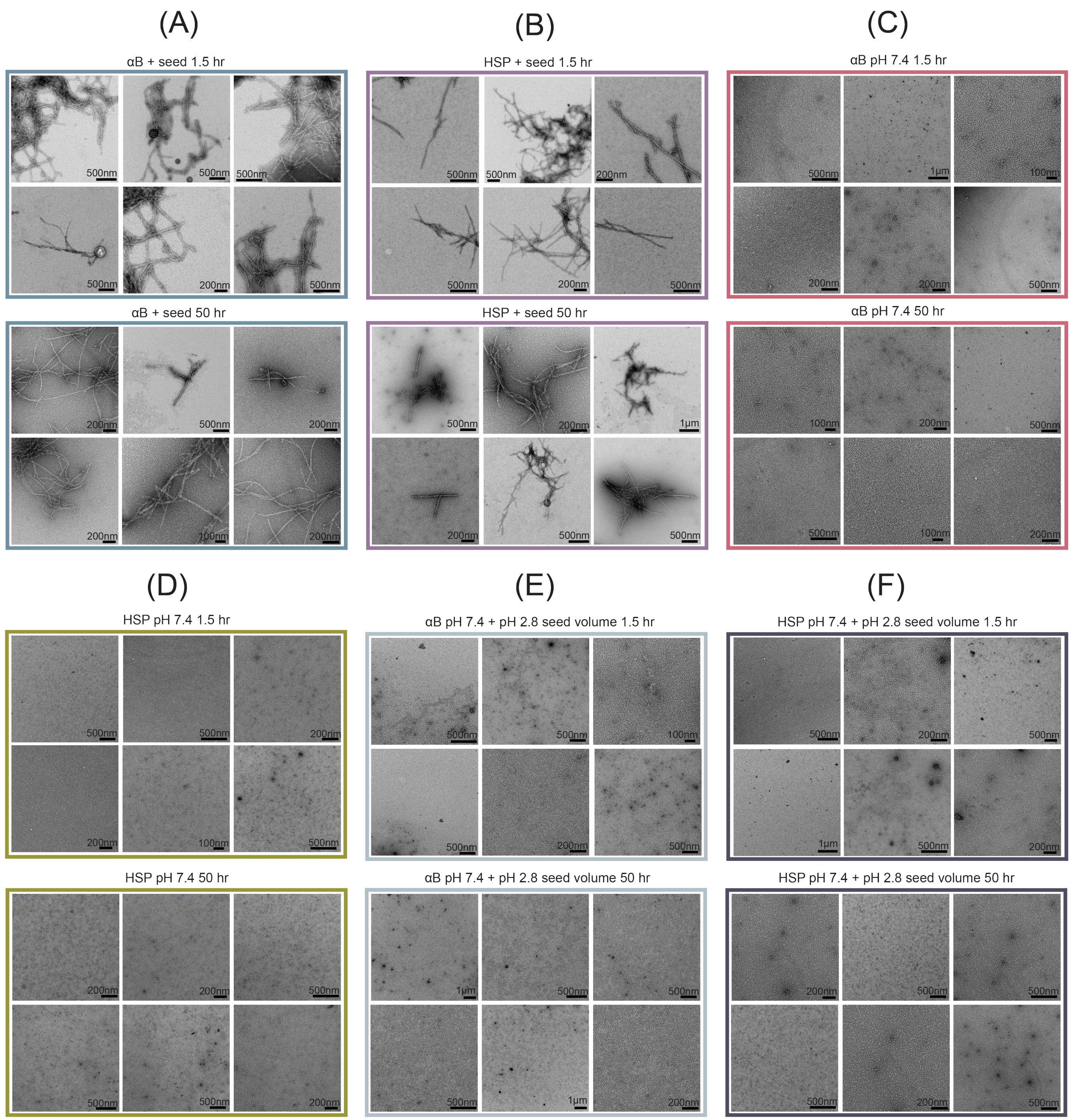


**Figure S8: Additional TEM controls supporting main text Figure 3.** (A-F) TEM images of chaperones + seed, chaperones at pH 7.4, and chaperones + pH 2.8 seed volume at time points 1.5 hours and 50 hours. Concentrations were 64 $\mu$M seed, 5.46 $\mu$M $\alpha$B-crystallin, and 4.83 $\mu$M HSP27.

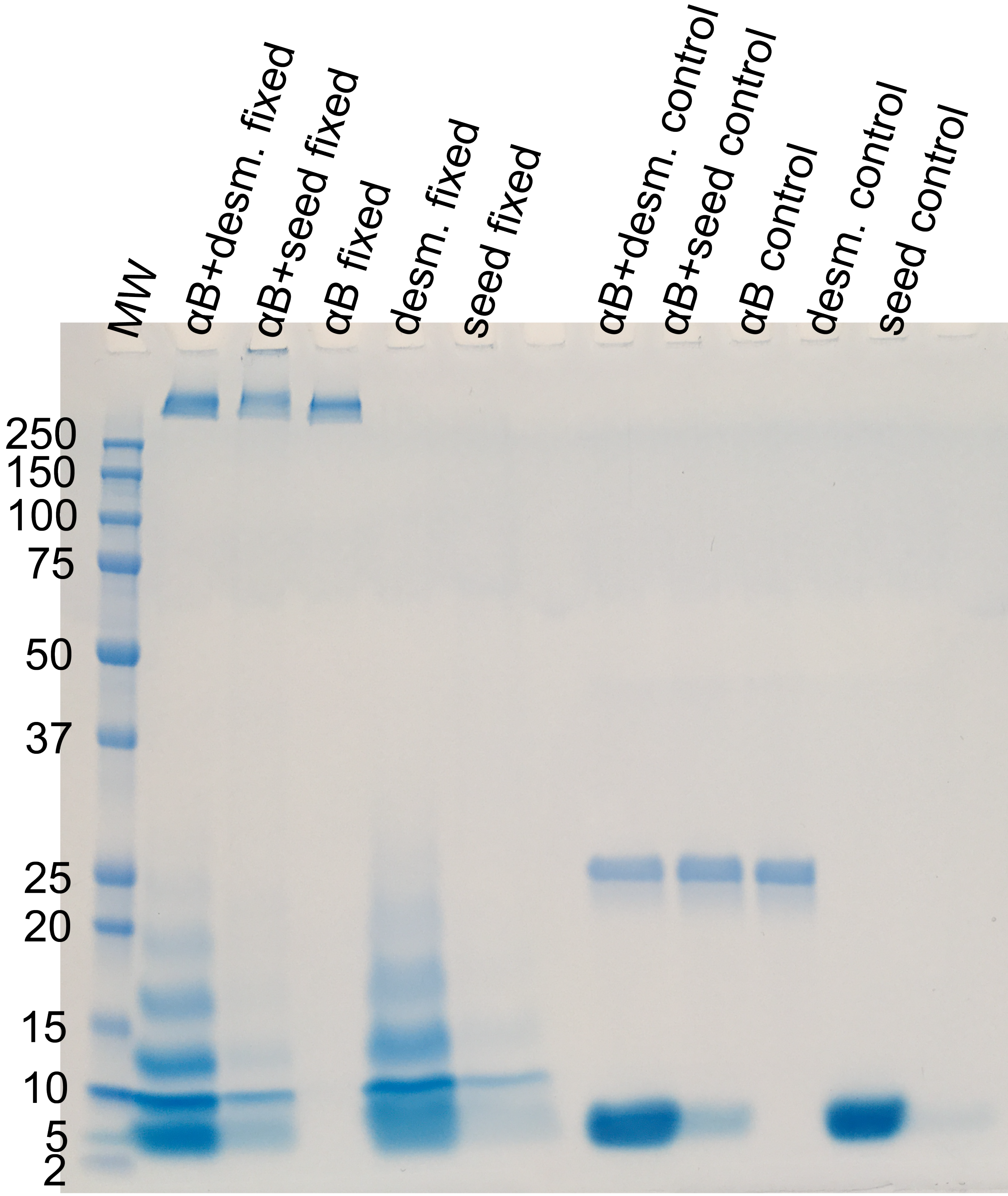


**Figure S9: SDS PAGE gel after crosslinking potentially indicating active chaperones are in ≥12mer form.** The fixed samples show that the chaperone does not show a band between 22 kDa and 250 kDa when combined with desmin fragment. If the chaperone was active as a dimer interacting with between 1 to 52 molecules of desmin fragment, or if active as a hexamer interacting with between 1 to 33 molecules of desmin fragment, or if active as a 12mer interacting with between 1 to 2 molecules of desmin, then we would see a band in this region.

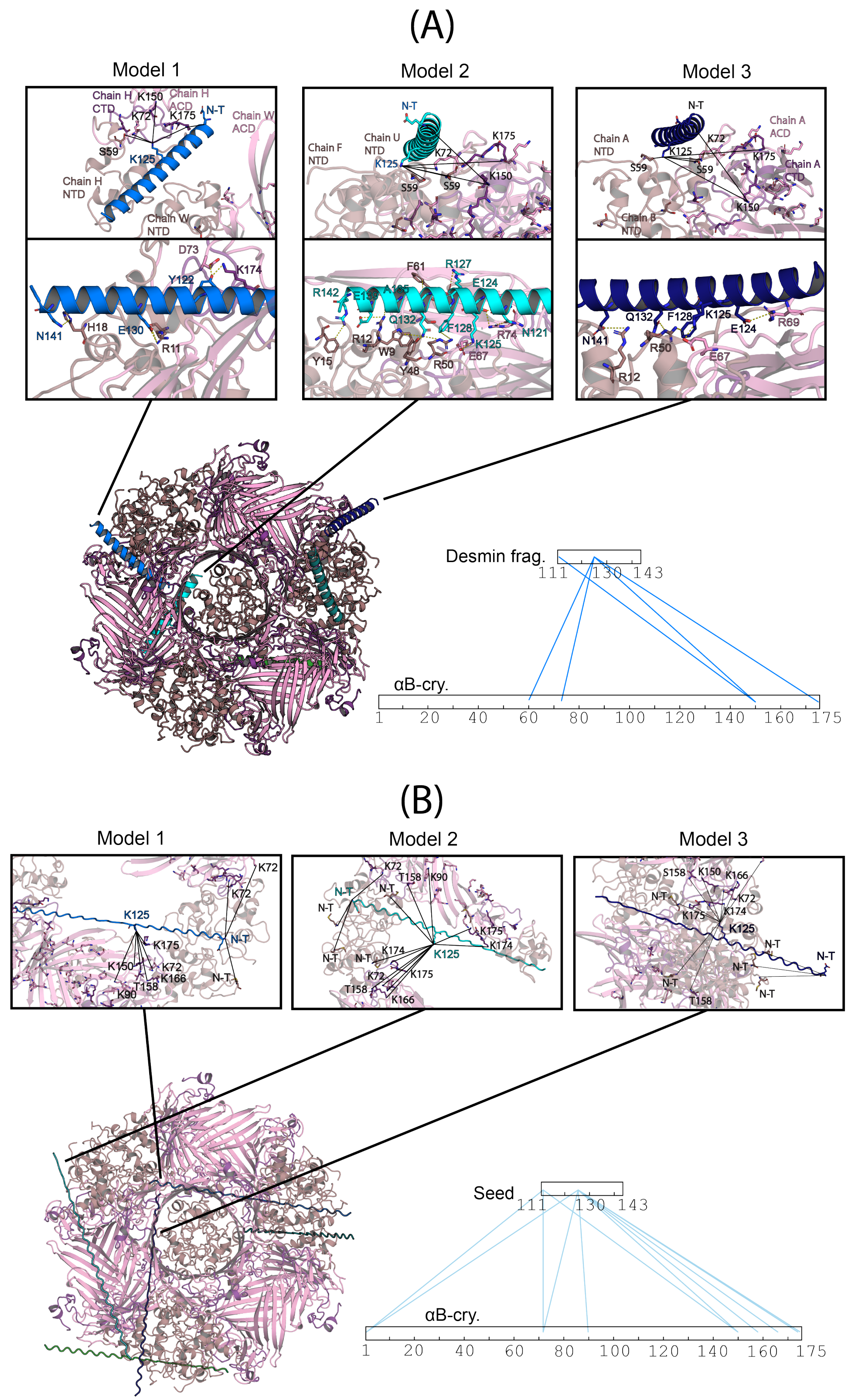


**Figure S10: Top scoring Hdock models of desmin fragment + $\alpha$B-crystallin with overlaid XL-MS observed cross-links.** (A) Top 5 scoring Hdock models of desmin fragment monomer-$\alpha$B-crystallin complex and (B) desmin fragment seed-$\alpha$B-crystallin complex (model 1: blue, model 2: cyan, model 3: navy, model 4:dark teal, model 5: green). Top insets in A and B show the top three scoring HDOCK models with labels and lines indicating cross-linked residues from XL-MS. XL-MS cross-links shown between monomer and $\alpha$B lines (dark blue, A bottom right) and seed and $\alpha$B lines (light blue, B bottom right).

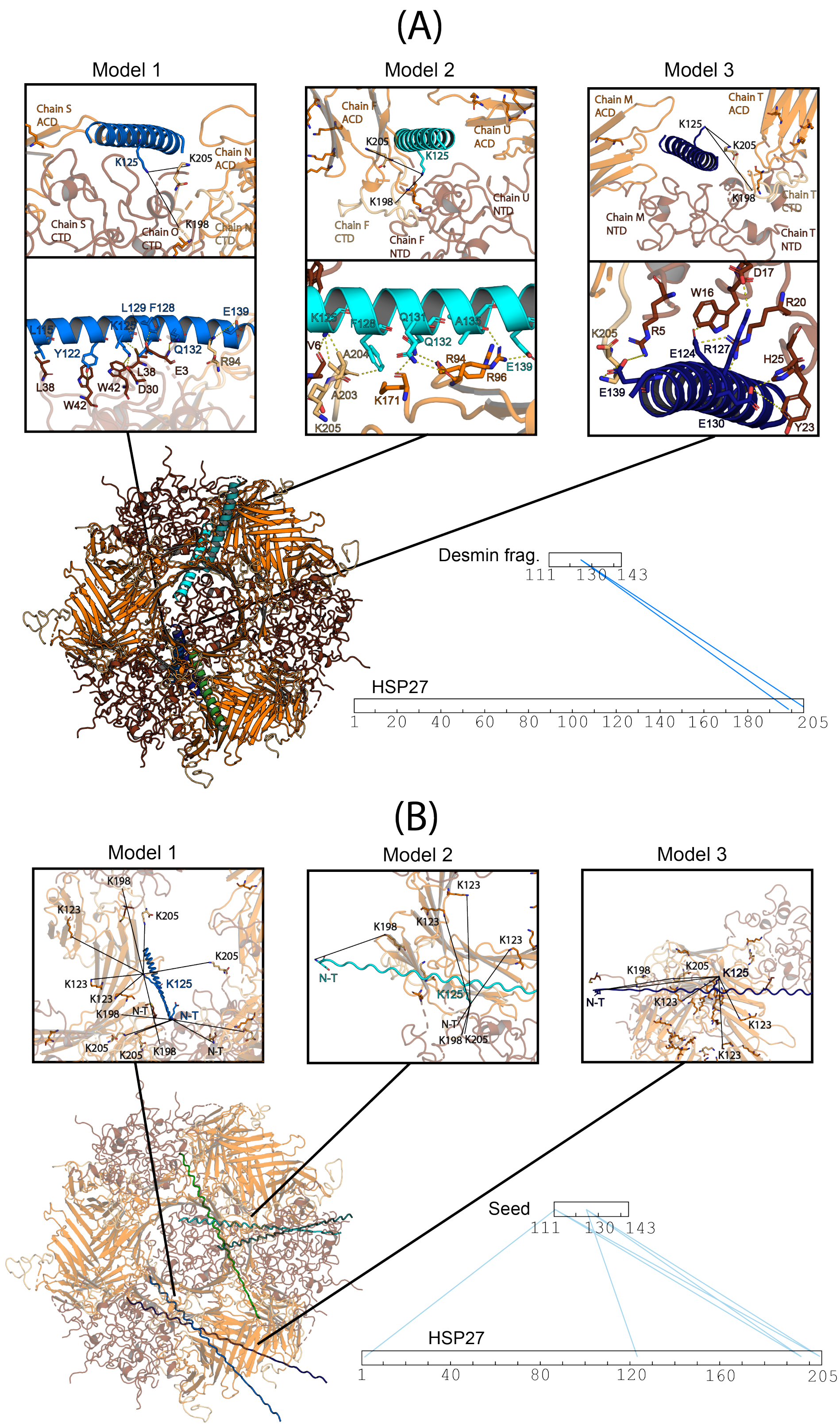


**Figure S11: Top scoring Hdock models of desmin fragment monomer + HSP27 with overlaid XL-MS observed cross-links.** (A) Top 5 scoring Hdock models of desmin fragment monomer-HSP27 complex and (B) desmin fragment seed-HSP27 complex (model 1: blue, model 2: cyan, model 3: navy, model 4:dark teal, model 5: green). Top insets in A and B show the top three scoring HDOCK models with labels and lines indicating cross-linked residues from XL-MS. XL-MS cross-links shown between monomer and HSP27 lines (dark blue, A bottom right) and seed and HSP27 lines (light blue, B bottom right).

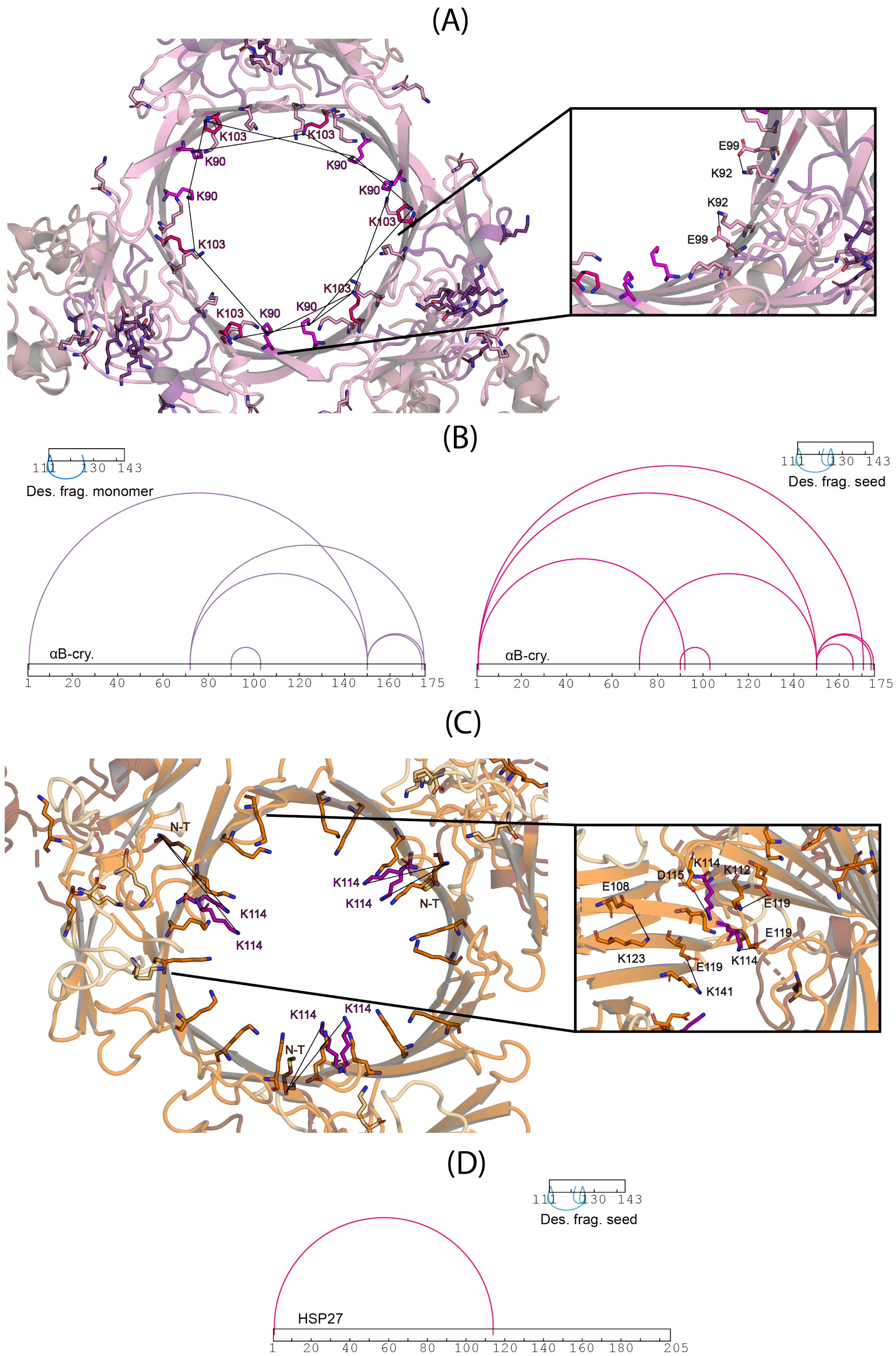


**Figure S12: XL-MS intra-links of chaperone proteins $\alpha$B-crystallin and HSP27.** (A) $\alpha$B-crystallin complex showing most of the lysines in the ACD point inward and are crosslinked to each other. Inset shows other lysines within the ACD form salt bridges to stabilize the dimer-dimer interface and are therefore inaccessible to interact with desmin fragment. (B) Intra-links (self-links) mapped onto $\alpha$B-crystallin when incubated with desmin fragment monomer (left) or desmin fragment seed (right). Intra-links of desmin fragment monomer or seed are also shown. (C) HSP27 complex showing most of the lysines in the ACD point inward and are crosslinked to each other. Inset shows other lysines within the ACD form salt bridges to stabilize the dimer-dimer interface and are therefore inaccessible to interact with desmin fragment. (D) Intra-links (self-links) mapped onto HSP27 when incubated with desmin fragment seed, no intra-links were observed of HSP27 when incubated with desmin fragment monomer.

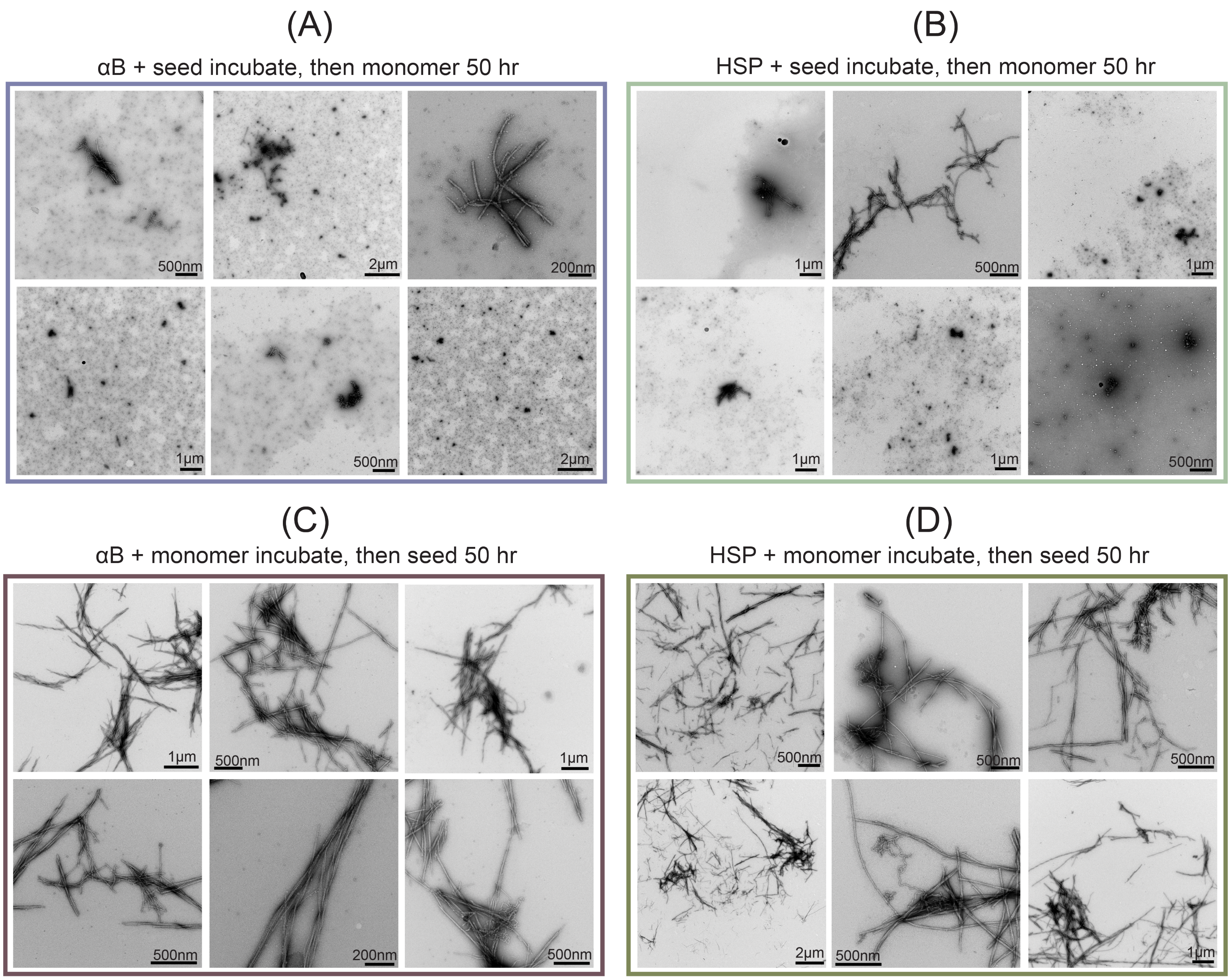


**Figure S13: TEM data supporting main text Figure 5.** (A-B) TEM images of chaperone incubated with seed first, and then monomeric desmin added. (C-D) TEM images of chaperone incubated with monomer first, then seed added. TEMs shown after 50 hours. Six representative images are shown for each condition.

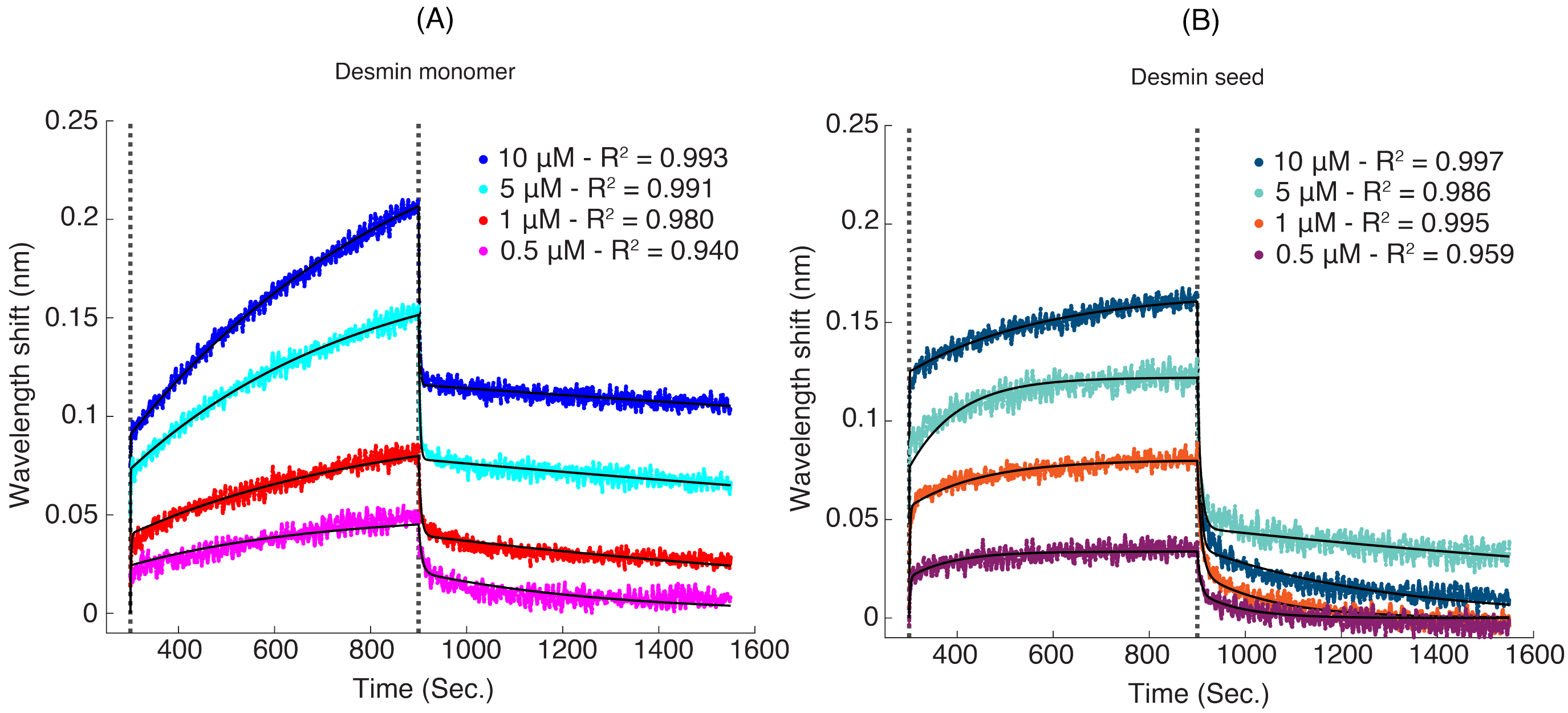


**Figure S14: Bio-layer interferometry showing that chaperone $\alpha$B-crystallin is capable of binding to both (A) desmin monomer and (B) desmin seed.**

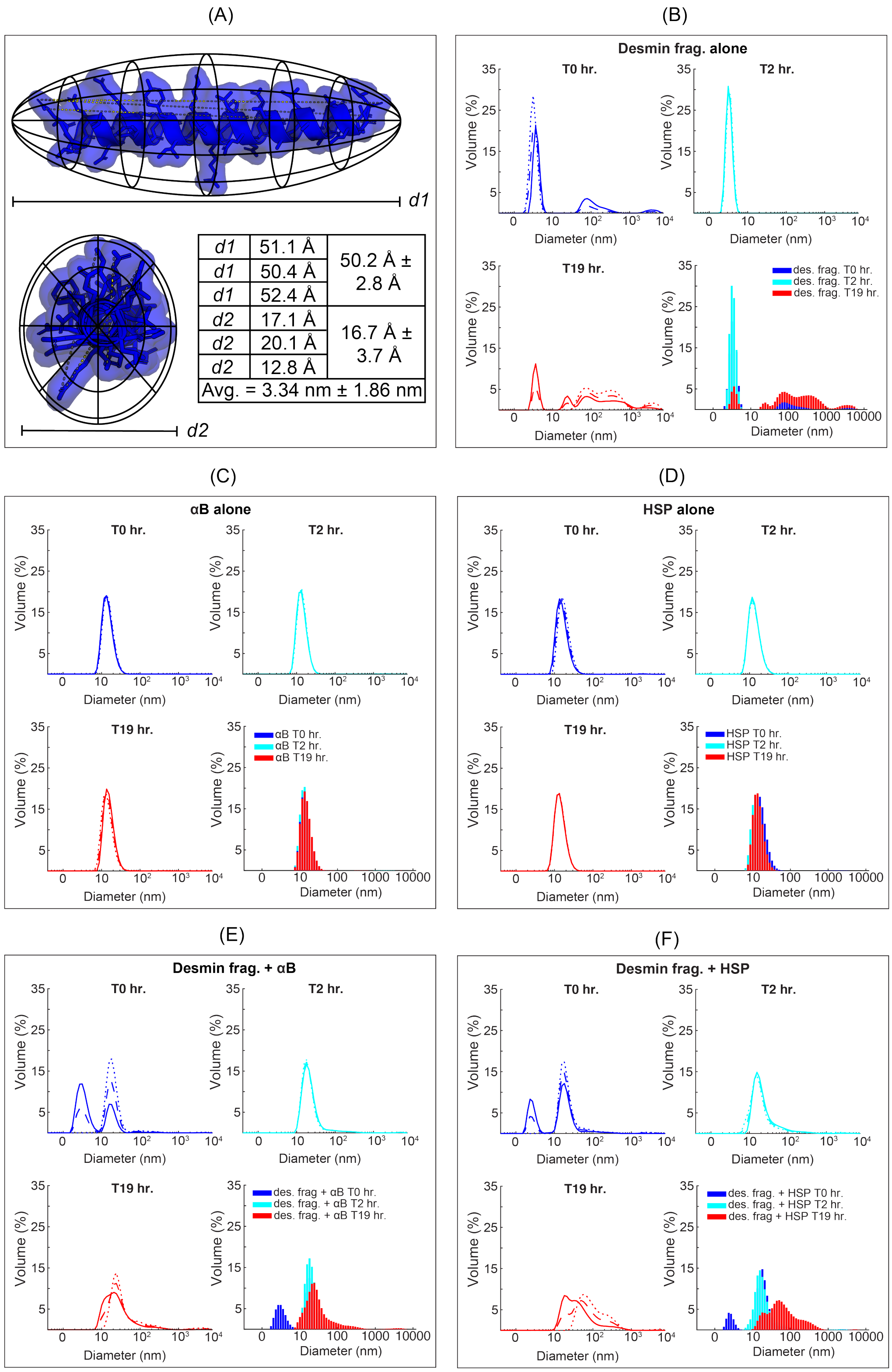


**Figure S15: DLS data supporting main text Figure 5.** (A) Pymol images of monomeric desmin fragment structure as predicted by AlphaFold showing the shape is best described by an ellipsoid with major (d1) and minor (d2) diameters. Averaged diameters are equal to a diameter of 3.34 nm ± 1.86 nm, similar to the observed hydrodynamic diameter of 3.4 nm. (B-F) Averaged intensity by volume plots of desmin fragment alone, $\alpha$B-crystallin alone, HSP27 alone, $\alpha$B-crystallin + desmin fragment, and HSP27 + desmin fragment at three different time points. Two independent preparations with triplicate dependent measurements were averaged. All measurements at each condition were then averaged (N=6 per condition).

**Table S1: XL-MS observed cross-links with desmin fragment and $\alpha$B-crystallin** Observed cross-links between desmin fragment monomer or seed and $\alpha$B-crystallin are shown. *Denotes where cross-links with a maximum XlinkX score below the cutoff threshold (>50) are included, since they were observed across other replicates.

| Sample | Cross-linked fragments | Positions (Des. - Chap.) | Rep. number | Max XlinkX score |
|---|---|---|---|---|
| Des. frag. mono. + $\alpha$B | FANYIE[K]VR-[K]QVSGPER | K125 - K150 | 1 | 88.68 |
| | | | 2 | 95.57 |
| | | | 3 | 70.98 |
| | | | 4 | 70.98 |
| | FANYIE[K]VR-LE[K]DR | K125 - K72 | 1 | 83.00 |
| | | | 2 | 85.35 |
| | | | 3 | N/A |
| | | | 4 | 56.94 |
| | FANYIE[K]VR-AP[S]WFDTGLSEMRLEK | K125 - S59 | 1 | 108.67 |
| | | | 2 | N/A |
| | | | 3 | 83.00 |
| | | | 4 | N/A |
| | [+]ELQELNDR-[K]QVSGPER | N-term - K150 | 1 | 47.05* |
| | | | 2 | N/A |
| | | | 3 | 72.62 |
| | | | 4 | N/A |
| | FANYIE[K]VR-EEKPAVTAAPK[K] | K125 - K175 | 1 | N/A |
| | | | 2 | N/A |
| | | | 3 | 83.00 |
| | | | 4 | 68.55 |
| Des. frag. seed + $\alpha$B | [+]ELQELNDR-LE[K]DR | NTerm - K72 | 1 | 61.30 |
| | | | 2 | N/A |
| | | | 3 | 95.62 |
| | FANYIE[K]VR-LE[K]DR | K125 - K72 | 1 | 87.66 |
| | | | 2 | 112.75 |
| | | | 3 | 107.79 |
| | FANYIE[K]VR-[K]QVSGPER | K125 - K150 | 1 | 126.31 |
| | | | 2 | 125.48 |
| | | | 3 | 125.57 |
| | [+]ELQELNDR-[K]QVSGPER | NTerm - K150 | 1 | 65.33 |
| | | | 2 | 79.03 |
| | | | 3 | N/A |
| | FANYIE[K]VR-TIPITREEKPAVTAAPK[K] | K125 - K175 | 1 | 100.48 |
| | | | 2 | 74.21 |
| | | | 3 | 96.34 |
| | [+]ELQELNDRFANYIEK-[+]MDIAIHHPWIR | NTerm - NTerm | 1 | 65.80 |
| | | | 2 | 32.31* |
| | | | 3 | 20.63* |
| | FANYIE[K]VR-HFSPEEL[K]VK | K125 - K90 | 1 | 142.43 |
| | | | 2 | 163.94 |
| | | | 3 | 105.98 |
| | ELQELNDRFANYIE[K]VR-[+]MDIAIHHPWIR | K125 - NTerm | 1 | 82.38 |
| | | | 2 | 71.74 |
| | | | 3 | 115.98 |
| | FANYIE[K]VR-[T]IPITREEKPAVTAAPK | K125 - T158 | 1 | N/A |
| | | | 2 | 68.55 |
| | | | 3 | 82.38 |
| | FANYIE[K]VR-TIPITREEKPAVTAAP[K]K | K125 - K174 | 1 | 96.46 |
| | | | 2 | 23.75* |
| | | | 3 | 62.84 |

**Table S2: XL-MS observed cross-links with desmin fragment and HSP27** Observed cross-links between desmin fragment monomer or seed and HSP27 are shown. *Denotes where cross-links with a maximum XlinkX score below the cutoff threshold (>50) are included, since they were observed across other replicates.

| Sample | Cross-linked fragments | Positions (Des. - Chap.) | Rep. number | Max XlinkX score |
|---|---|---|---|---|
| Des. frag. mono. + HSP27 | FANYIE[K]VR-AQLGGPEAA[K]SDETAAK | K125 - K198 | 1 | 55.43 |
| | | | 2 | 137.2 |
| | | | 3 | N/A |
| | ELQELNDRFANYIE[K]VR-AQLGGPEAAKSDETAA[K] | K125 - K205 | 1 | N/A |
| | | | 2 | 124.43 |
| | | | 3 | 43.08* |
| Des. frag. seed + HSP27 | FANYIE[K]VR-TKDGVVEITG[K]HEERQDEHGYISR | K125 - K123 | 1 | 136.48 |
| | | | 2 | 89.4 |
| | | | 3 | 118.78 |
| | FANYIE[K]VR-AQLGGPEAA[K]SDETAAK | K125 - K198 | 1 | 127.22 |
| | | | 2 | 115.98 |
| | | | 3 | 147.92 |
| | ELQELNDRFANYIE[K]VR-AQLGGPEAAKSDETAA[K] | K125 - K205 | 1 | 124.43 |
| | | | 2 | 108.67 |
| | | | 3 | 112.75 |
| | [E]LQELNDRFANYIEK-[M]TER | NTerm - NTerm | 1 | 176.33 |
| | | | 2 | N/A |
| | | | 3 | 191.86 |
| | [+]ELQELNDR-AQLGGPEAAKSDETAA[K] | NTerm - K205 | 1 | 139.69 |
| | | | 2 | N/A |
| | | | 3 | 131.72 |
| | [+]ELQELNDR-AQLGGPEAA[K]SDETAAK | NTerm - K198 | 1 | 107.73 |
| | | | 2 | N/A |
| | | | 3 | 35.11* |
